# Supplementary material for: Developing a pathway-independent and full-autonomous global resource allocation strategy to dynamically switching phenotypic states
Source: Nat Commun. 2020 Nov 2;11:5521. doi: 10.1038/s41467-020-19432-2 (PMC7606477; doi:10.1038/s41467-020-19432-2)
Supplement: Supplementary file 1 — Supplementary Information [file 41467_2020_19432_MOESM1_ESM.pdf]

Developing a pathway-independent and full-autonomous global resource allocation strategy to dynamically switch phenotypic states

Junjun Wu[1*], Meijiao Bao[1], Xuguo Duan[2], Peng Zhou[1], Caiwen Chen[1], Jiahua Gao[1], Shiyao Cheng[1], Qianqian Zhuang[3], Zhijun Zhao[4]

[1] College of Food Science and Technology, Nanjing Agricultural University, Nanjing, Jiangsu 210095, China.

[2] Department of Food Science and Technology, College of Light Industry and Food Engineering, Nanjing Forestry University, Nanjing, 210037, China.

[3] State Key Laboratory of Biobased Material and Green Papermaking, School of Bioengineering, Qilu University of Technology, Jinan 250353, China.

[4] Biorefinery Laboratory, Shanghai Advanced Research Institute, Chinese academy of sciences, 99 Haike Road, Shanghai 201210, China.

[*] Corresponding author:

Junjun Wu

Mailing address: College of Food Science and Technology, Nanjing Agricultural University, 1 Weigang Road, Nanjing, Jiangsu, P.R. China

Phone: +86 25 84396989, Fax: +86 25 84399090

E-mail: wujunjun@njau.edu.cn

Supplementary methods

Construction of QS systems in *E. coli*

All constructed plasmids were verified by both colony PCR and Sanger sequencing. Primers used in this section were shown in Supplementary Table 3. Ezup Column Bacteria Genomic DNA Purification Kit (Sangon Biotech, Shanghai, China) was utilized to prepare bacterial chromosomal DNA. For constructing signal plasmids (Fig. 1A), primers Pf_PluxI-luxI(*Eco*NI)/Pr_PluxI-luxI(*Xho*I), Pf_PlasI-lasI(*Eco*NI)/Pr_PlasI-lasI(*Xho*I), Pf_Pbd-agrBD(*Eco*NI)/Pr_Pbd-agrBD(*Xho*I), Pf_Pc-ccfA(*Eco*NI)/Pr_Pc-ccfA(*Xho*I) were used to clone *luxI*, *lasI*, *agrBD* and *ccfA* associating with their native promoters from *V. fischeri*, *P. aeruginosa*, *S. aureus*, and *E. faecalis* genomic DNA into *Eco*NI/*Xho*I sites of pETDuet-1, resulting in plasmids of pETD-PluxI-*luxI*, pETD-PlasI-*lasI*, pETD-Pbd-*agrBD*, pETD-Pc-*ccfA*, respectively. Similarly, for constructing regulator plasmids, primers Pf_PluxR-luxR(*Eco*NI)/Pr_PluxR-luxR(*Xho*I), Pf_PlasR-lasR(*Eco*NI)/Pr_PlasR-lasR(*Xho*I), Pf_Pac-agrAC(*Eco*NI)/Pr_Pac-agrAC(*Xho*I), Pf_Pr-prgX(*Eco*NI)/Pr_Pr-prgX(*Xho*I) were used to clone *luxR*, *lasR*, *agrAC* and *prgX* associating with their native promoters from corresponding genomic DNA into *Eco*NI/*Xho*I sites of pACYCDuet-1, resulting in plasmids of pACYC-PluxR-*luxR*, pACYC-PlasR-*lasR*, pACYC-Pac-*agrAC*, pACYC-Pr-*prgX*, respectively.

For constructing reporter plasmid, primers Pf_PluxI(*pfo*I)/Pr_PluxI(*Xho*I),

Pf_PlasB(*pfo*I)/Pr_PlasB(*Xho*I), Pf_PlasA(*pfo*I)/Pr_PlasA(*Xho*I), Pf_PhcnABC(*pfo*I)/Pr_PhcnABC(*Xho*I), Pf_PprgQ(*pfo*I)/Pr_PprgQ(*Xho*I) were used to clone response promoter PluxI, PlasB, PlasA, PhcnABC, PprgQ from corresponding genomic DNA into *pfo*I/*Xho*I sites of pCOLADuet-1, resulting in plasmids of pCOLA-PluxI, pCOLA-PlasB, pCOLA-PlasA, pCOLA-PhcnABC, pCOLA-PprgQ, repectively. All the reverse primers used for reporter plasmid construction contained *Nco*I/*Bam*HI site, facilitating further insertion of GFP with primers Pf_GFP(*Nco*I) and Pr_GFP(*Bam*HI). This resulted in reporter plasmids of pCOLA-PluxI-GFP, pCOLA-PlasB-GFP, pCOLA-PlasA-GFP, pCOLA-PhcnABC-GFP, pCOLA-PprgQ-GFP, respectively.

To fuse *agrB* tagged with native promoter Pbd (Pbd-AgrBD) to GFP (Fig. 1C), the stop codon of *agrB* was removed and a Gly-Ser-Gly linker was introduced between the open reading frame of *agrB* and GFP through two rounds of PCR [1]. The first round rendered mutations to Pbd-*agrB* and GFP utilizing two sets of primers include Pf_Pbd-agrB-GSG-GFP(*Eco*NI)/Pr_Pbd-agrB-GSG-GFP, Pf_agrB-GSG-GFP/Pr_agrB-GSG-GFP(*Xho*I). Each amplifying product was purified as templates for second round PCR. Next, these two PCR products were connected through overlapping extension PCR by primers Pf_Pbd-agrB-GSG-GFP(*Eco*NI)/Pr_agrB-GSG-GFP(*Xho*I), resulting in approximately 1.4 kb fused gene construct encoding Pbd-*agrB*, the three amino acid linker, and GFP.

Similarly, primer sets of Pf_Pac-agrC-GSG-GFP(*Eco*NI)/Pr_Pac-agrC-GSG-GFP,

Pf_agrC-GSG-GFP/Pr_agrC-GSG-GFP(*Xho*I) were used to fuse *agrC* tagged with native promoter Pac (Pac-*agrC*) to GFP; Primer sets of Pf_Pr-prgX-GSG-GFP(*Eco*NI)/Pr_Pr-prgX-GSG-GFP, Pf_prgX-GSG-GFP/Pr_prgX-GSG-GFP(*Xho*I) were used to fuse *prgX* tagged with native promoter Pr (Pr-*prgX*) to GFP; Primer sets of Pf_Pc-ccfA-GSG-GFP(*Eco*NI)/Pr_Pc-ccfA-GSG-GFP, Pf_ccfA-GSG-GFP/ Pr_ccfA-GSG-GFP(*Xho*I) were used to fuse *ccfA* tagged with native promoter Pc (Pc-*ccfA*) to GFP;

Primer sets of Pf_Ptrc-agrB-GSG-GFP(*Eco*NI)/Pr_Ptrc-agrB-GSG-GFP, Pf_agrB-GSG-GFP/Pr_agrB-GSG-GFP(*Xho*I) were used to fuse *agrB* tagged with Ptrc promoter (Ptrc-*agrB*) to GFP; Primer sets of Pf_Ptrc-agrC-GSG-GFP(*Eco*NI)/Pr_Ptrc-agrC-GSG-GFP, Pf_agrC-GSG-GFP/Pr_agrC-GSG-GFP(*Xho*I) were used to fuse *agrC* tagged with Ptrc promoter (Ptrc-*agrC*) to GFP; Primer sets of Pf_Ptrc-prgX-GSG-GFP(*Eco*NI)/Pr_Ptrc-prgX-GSG-GFP, Pf_prgX-GSG-GFP/Pr_prgX-GSG-GFP(*Xho*I) were used to fuse *prgX* tagged with Ptrc promoter (Ptrc-*prgX*) to GFP; Primer sets of Pf_Ptrc-ccfA-GSG-GFP(*Eco*NI)/Pr_Ptrc-ccfA-GSG-GFP, Pf_ccfA-GSG-GFP/Pr_ccfA-GSG-GFP(*Xho*I) were used to fuse *ccfA* tagged with Ptrc promoter (Ptrc-*ccfA*) to GFP; Primers Pf_Ptrc-GFP(*Eco*NI) and Pr_Ptrc-GFP(*Xho*I) were used to tag GFP with Ptrc promoter.

Finally, all the fusion proteins were inserted into *Eco*NI/*Xho*I sites of

pACYCDuet-1, yielding plasmids of pACYC-Pbd-*agrB*-GSG-GFP, pACYC-Pac-*agrC*-GSG-GFP, pACYC-Pr-*prgX*-GSG-GFP, pACYC-Pc-*ccfA*-GSG-GFP, pACYC-Ptrc-*agrB*-GSG-GFP, pACYC-Ptrc-*agrC*-GSG-GFP, pACYC-Ptrc-*prgX*-GSG-GFP, pACYC-Ptrc-*ccfA*-GSG-GFP, pACYC-Ptrc-GFP, respectively.

Primer sets of Pf_Ptrc-luxI(*Eco*NI)/Pr_Ptrc-luxI(*Xho*I), Pf_Ptrc-lasI(*Eco*NI)/ Pr_Ptrc-lasI(*Xho*I), Pf_Ptrc-agrBD(*Eco*NI)/Pr_Ptrc-agrBD(*Xho*I), Pf_Ptrc-ccfA(*Eco*NI)/Pr_Ptrc-ccfA(*Xho*I) were used to tag *luxI*, *lasI*, *agrBD*, *ccfA* with Ptrc promoter. The resulting PCR products were further inserted into pETDuet-1, yielding signal plasmids under Ptrc promoter of pETD-Ptrc-*luxI*, pETD-Ptrc-*lasI*, pETD-Ptrc-*agrBD*, pETD-Ptrc-*ccfA*, respectively; Similarly, primer sets of Pf_Ptrc-luxR(*Eco*NI)/Pr_Ptrc-luxR(*Xho*I), Pf_Ptrc-lasR(*Eco*NI)/Pr_Ptrc-lasR(*Xho*I), Pf_Ptrc-agrAC(*Eco*NI)/Pr_Ptrc-agrAC(*Xho*I), Pf_Ptrc-prgX(*Eco*NI)/ Pr_Ptrc-prgX(*Xho*I) were used to construct regulator plasmids with Ptrc promoter of pACYC-Ptrc-*luxR*, pACYC-Ptrc-*lasR*, pACYC-Ptrc-*agrAC*, pACYC-Ptrc-*prgX*, respectively (Fig. 1D).

Generation of different variants of PluxI promoters

Primers used in this section were shown in Supplementary Table 4. For generation of promoter I1-I3, site-directed mutagenesis (TaKaRa MutanBEST Kit, TaKaRa Biotechnology, Dalian, China) was utilized associate with primer sets of Pf_I1-I3/Pr_I1, Pf_I1-I3/Pr_I2, Pf_I1-I3/Pr_I3 and template pCOLA-PluxI-GFP, resulting in plasmids of pCOLA-PluxI I1-GFP, pCOLA-PluxI I2-GFP, pCOLA-PluxI

I3-GFP, respectively. For generation of I4-I12, primer sets of Pf_Ii (*pfo*I)/Pr_I4-I12(*Xho*I) (i=4-12) were used to amplify corresponding regions from pCOLA-PluxI-GFP into *pfo*I/*Xho*I sites of pCOLADuet-1, resulting in plasmids of pCOLA-PluxI Ii-GFP (i=4-12). To tag *luxI* with PluxI variants of I9-I11, primer sets of Pf_Ii-luxI(*Eco*NI)/Pr_I9-I11(*Xho*I) (i=9-11) were used to amplify corresponding regions from pETD-PluxI-*luxI* into *Eco*NI/*Xho*I sites of pETDuet-1, resulting in plasmids of pETD-PluxI Ii-*luxI* (i=9-11).

Construction of QS components tagged with different strength promoters

Primers used in this section were shown in Supplementary Table 5. Primer sets of Pf_Pi-prgX-GSG-GFP(*Eco*NI)/Pr_(P1-P6)-prgX-GSG-GFP(*Xho*I) were used to amply corresponding regions including *prgX* tagged with Pi promoter, the three amino acid linker, and GFP, from pACYC-Pr-*prgX*-GSG-GFP into *Eco*NI/*Xho*I sites of pACYCDuet-1, resulting in plasmids of pACYC-Pi-*prgX*-GSG-GFP (i=1-6). Primer set of Pf_P1-prgX-GSG-GFP(*Eco*NI)/Pr_(P1-P6)-prgX-GSG-GFP(*Xho*I) were used to amply corresponding regions from pACYC-Pr-*prgX*-GSG-GFP into *Eco*NI/*Xho*I sites of pCDFDuet-1, resulting in pCDFD-P1-*prgX*-GSG-GFP. Primer sets of Pf_Trc-ccfA(G)(PET)/Pr_Trc-ccfA(G), Pf_Trc-prgZ(G)/Pr_Trc-prgZ(G)(PET) were used to amplify *ccfA* and *prgZ* tagged with Ptrc promoter, respectively, from *E. faecalis* genomic DNA into *Eco*NI/*Xho*I site of pETDuet-1 through Gibson assembly kit (New England Biolabs), resulting in pETD-Ptrc-*ccfA*-Ptrc-*prgZ*.

To examine QVS behaviors independently with signal auto-inducers and regulator proteins placed in the same plasmid, primer sets of

Pf_Pi-luxI(G)(PACYC)/Pr_Pi-luxI(G), Pf_Pi-luxR(G)/Pr_P1-P6-luxR(G)(PACYC) were used to amplify *luxI* and *luxR* with Pi promoters, respectively, from *V. fischeri* genomic DNA into *Eco*NI/*Xho*I site of pACYCDuet-1 through Gibson assembly kit (New England Biolabs), resulting in plasmids of pACYC-Pi-*luxI*-Pi-*luxR* (i=1-6). Similarly, to examine QES behaviors independently, primer sets of Pf_P1-prgX(G)(PACYC)/Pr_P1-prgX(G)-Pi-ccfA, Pf_Pi-ccfA(G)/Pr_ccfA(G), Pf_Ptrc-prgZ(G)/Pr_Ptrc-prgZ(G)(PACYC) were used to amplify *prgX* with P1 promoter, *ccfA* with Pi promoter, *prgZ* with Ptrc promoter through Gibson assembly kit (New England Biolabs), from *E. faecalis* genomic DNA into *Eco*NI/*Xho*I site of pACYCDuet-1, resulting in plasmids of pACYC-P1-*prgX*-Pi-*ccfA*-Ptrc-*prgZ* (i=1-6). Primers Pf_mKate2(*Nco*I)/Pr_mKate2(*Bam*HI) were used to clone mKate2 into *Nco*I/*Bam*HI site of pCOLA-PprgQ, resulted in pCOLA-PprgQ-mKate2.

To assemble QVS and QES systems into a single cell, primer sets of Pf_Pi-luxI-Pi-luxR(G)(PACYC)/Pr_P1-P6-luxI-P1-P6-luxR(G), Pf_P1-prgX-P1-P6-ccfA-Ptrc-prgZ(G)/ Pr_P1-prgX-P1-P6-ccfA-Ptrc-prgZ(PACYC) were used to amplify *luxI* and *luxR* with Pi promoter, *prgX* with P1, *ccfA* with Pj, *prgZ* with Ptrc promoter from pACYC-Pi-*luxI*-Pi-*luxR* and pACYC-P1-*prgX*-Pi-*ccfA*-Ptrc-*prgZ*, respectively, and into *Eco*NI/*Xho*I site of pACYCDuet-1 through Gibson assembly kit (New England Biolabs), resulting in pACYC-Pi-*luxI*-Pi-*luxR*-P1-*prgX*-Pj-*ccfA*-Ptrc-*prgZ* (i and j=1-6, respectively). Primer sets of Pf_PluxI I11-GFP(G)(PCOLA)/Pr_PluxI I11-GFP(G), Pf_PprgQ-mKate2(G)/Pr_PprgQ-mKate2(G)(PCOLA) were used to amplify GFP

tagged with PluxI I11 response promoter and mKate2 tagged with PprgQ response promoter from pCOLA-PluxI I11-GFP and pCOLA-PprqQ-mKate2, respectively, into *pfo*I/*Xho*I sites of pCOLADuet-1 through Gibson assembly kit (New England Biolabs), resulting in pCOLA-PluxI I11-GFP-PprqQ-mKate2.

Construction of global resource allocators

Primers used in this section were shown in Supplementary Table 6. Modified GFP without recognition sites of MazF (GFP(M) was synthesized by GenScript (Nanjing, China) and inserted into *Nco*I/*Bam*HI site of pCOLA-PluxI I11-GFP with restriction enzymes *Nco*I and *Bam*HI, resulting in pCOLA-PluxI I11-GFP(M). Primers Pf_MazF(*Nco*I)/Pr_MazF(*Bam*HI) were used to clone *mazF* into *Nco*I/*Bam*HI site of pCOLA-PprgQ, resulted in pCOLA-PprgQ-*mazF*. Primer sets of Pf_PluxI I11-GFP-mazF(G)(PCOLA)/Pr_PluxI I11-GFP-mazF(G), Pf_GFP-PprgQ-mazF(G)/Pr_GFP-PprgQ-mazF(G)(PCOLA) were used to clone GFP(M) tagged with PluxI I11 response promoter and *mazF* tagged with PprgQ response promoter from pCOLA-PluxI I11-GFP(M) and pCOLA-PprqQ-*mazF*, respectively, into *pfo*I/*Xho*I sites of pCOLADuet-1, resulting in response module of pCOLA-PluxI I11-GFP(M)-PprgQ-*mazF*.

To remove recognition sites of MazF in native QS system components, modified *luxI* (*luxI*(M)), *luxR* (*luxR*(M)), *ccfA* (*ccfA*(M)), *prgX* (*prgX*(M), *prgZ* (*prgZ*(M)) without recognition sites of MazF were synthesized by GenScript (Nanjing, China). Primer sets of Pf_P2-luxI(M)(G)(PACYC)/Pr_P2-luxI(M)(G), Pf_P2-luxR(M)(G)/Pr_P2-luxR(M)(G)(PACYC) were used to amplify *luxI*(M) and

LuxR(M) with P2 promoter from T-*luxI*(M), T-*luxR*(M), respectively, into *Eco*NI/*Xho*I site of pACYCDuet-1 through Gibson assembly kit (New England Biolabs), resulting in pACYC-P2-*luxI*(M)-P2-*luxR*(M). Primer sets of Pf_P1-prgX(M)(G) (PACYC)/Pr_P1-prgX(M)(G), Pf_P3-ccfA(M)(G)/Pr_P3-ccfA(M)(G), Pf_Ptrc-prgZ(M)(G)/Pr_Ptrc-prgZ(M)(G)(PACYC) were used to amplify *prgX*(M) with P1 promoter, *ccfA*(M) with P3 promoter, *prgZ*(M) with Ptrc promoter through Gibson assembly kit (New England Biolabs), from T-*prgX*(M), T-*ccfA*(M), T-*prgZ*(M), respectively, into *Eco*NI/*Xho*I site of pACYCDuet-1, resulting in pACYC-P1-*prgX*(M)-P3-*ccfA*(M)-Ptrc-*prgZ*(M). Primer sets of Pf_ P2-luxI(M)-P2-luxR(M)(G)(PACYC)/ Pr_P2-luxI(M)-P2-luxR(M)(G), Pf_P1-prgX-P3-ccfA-Ptrc-prgZ(G)/Pr_P1-prgX-P3-ccfA-Ptrc-prgZ(G)(PACYC) were used to amplify *luxI*(M) and *luxR*(M) with P2 promoter, *prgX* with P1, *ccfA* with P3, *prgZ* with Ptrc promoter from pACYC-P2-*luxI*(M)-P2-*luxR*(M) and pACYC-P1-*prgX*-P3-*ccfA*-Ptrc-*prgZ*, respectively, and into *Eco*NI/*Xho*I site of pACYCDuet-1, resulting in pACYC-P2-*luxI*(M)-P2-*luxR*(M)-P1-*prgX*-P3-*ccfA*-Ptrc-*prgZ*, which is denoted as LuxI(M), LuxR(M) in Fig. 8D. Primer sets of Pf_ P2-luxI-P2-luxR(G)(PACYC)/ Pr_P2-luxI-P2-luxR(G), Pf_P1-prgX(M)-P3-ccfA(M)-Trc-prgZ(M)(G)/ Pr_P1-prgX(M)-P3-ccfA(M)-Trc-prgZ(M)(G)(PACYC) were used to amplify *luxI* and *luxR* with P2 promoter, *prgX*(M) with P1, *ccfA*(M) with P3, *prgZ*(M) with Ptrc promoter from pACYC-P2-*luxI*-P2-*luxR* and

pACYC-P1-*prgX*(M)-P3-*ccfA*(M)-Trc-*prgZ*(M), respectively, and into *Eco*NI/*Xho*I site of pACYCDuet-1, resulting in pACYC-P2-*luxI*-P2-*luxR*-P1-*prgX*(M)-P3-*ccfA*(M)-Ptrc-*prgZ*(M), which is denoted as CcfA(M), PrgX(M), PrgZ(M) in Fig. 7D. Primer sets of Pf_ P2-luxI(M)-P2-luxR(M)(G)(PACYC)/ Pr_P2-luxI(M)-P2-luxR(M)(G), Pf_P1-prgX(M)-P3-ccfA(M)-Ptrc-prgZ(M)(G)/ Pr_P1-prgX(M)-P3-ccfA(M)-Ptrc-prgZ(M)(G)(PACYC) were used to amplify *luxI*(M) and *luxR*(M) with P2 promoter, *prgX*(M) with P1, *ccfA*(M) with P3, *prgZ*(M) with Ptrc promoter from pACYC-P2-*luxI*(M)-P2-*luxR*(M) and pACYC-P1-*prgX*(M)-P3-*ccfA*(M)-Ptrc-*prgZ*(M), respectively, and into *Eco*NI/*Xho*I site of pACYCDuet-1, resulting in pACYC-P2-*luxI*(M)-P2-*luxR*(M)-P1-*prgX*(M)-P3-*ccfA*(M)-Ptrc-*prgZ*(M), which is denoted as LuxI(M), LuxR(M), CcfA(M), PrgX(M), PrgZ(M).

Protection of host factors including transcriptional and translational machinery

Primers used in this section were shown in Supplementary Table 7. To remove recognition sites of MazF in host factors, modified β' (β'(M)), α2 (α2(M)), β (β(M)), EF-Ts (EF-Ts(M), S9 (S9(M)), S20 (S20(M)), L17 (L17(M)) without recognition sites of MazF were synthesized by GenScript (Nanjing, China). Primer sets of Pf_PI11-GFP(M)-PprgQ-mazF(PCOLA)/Pr_PI11-GFP(M)-PprgQ-mazF, Pf_PprgQ-β(M)/Pr_PprgQ-β(M)(PCOLA) were used to amplify GFP(M) with PluxI I11, *mazF* with PprgQ, and β(M) with PprgQ from pCOLA-PluxI I11-GFP(M)-PprqQ-*mazF*, T-β(M) (GenScript), respectively, and into *pfo*I/*Xho*I site

of pCOLADuet-1 through Gibson assembly kit (New England Biolabs), resulting in pCOLA-PluxI I11-GFP(M)-PprqQ-*mazF*-PprgQ-β(M). Primer sets of Pf_PI11-GFP(M)-PprgQ-mazF(PCOLA)/Pr_PI11-GFP(M)-PprgQ-mazF, Pf_PprgQ-β'(M)/Pr_PprgQ-β'(M)(PCOLA) were used to amplify GFP(M) with PluxI I11, *mazF* with PprgQ, and β'(M) with PprgQ from pCOLA-PluxI I11-GFP(M)-PprqQ-*mazF*, T-β'(M) (GenScript), respectively, and into *pfo*I/*Xho*I site of pCOLADuet-1 through Gibson assembly kit (New England Biolabs), resulting in pCOLA-PluxI I11-GFP(M)-PprqQ-*mazF*-PprgQ-β'(M). Primer sets of Pf_PI11-GFP(M)-PprgQ-mazF(PCOLA)/Pr_PI11-GFP(M)-PprgQ-mazF, Pf_PprgQ-α2(M)/Pr_PprgQ-α2(M)(PCOLA) were used to amplify GFP(M) with PluxI I11, *mazF* with PprgQ, and α2(M) with PprgQ from pCOLA-PluxI I11-GFP(M)-PprqQ-*mazF*, T-α2(M) (GenScript), respectively, and into *pfo*I/*Xho*I site of pCOLADuet-1 through Gibson assembly kit (New England Biolabs), resulting in pCOLA-PluxI I11-GFP(M)-PprqQ-*mazF*-PprgQ-α2(M). Primer sets of Pf_PI11-GFP(M)-PprgQ-mazF(PCOLA)/Pr_PI11-GFP(M)-PprgQ-mazF-β(M)-β'(M), Pf_PprgQ-β'(M)/Pr_PprgQ-β'(M)(PCOLA) were used to amplify GFP(M) with PluxI I11, *mazF* with PprgQ, β(M) with PprgQ, and β'(M) with PprgQ from pCOLA-PluxI I11-GFP(M)-PprqQ-*mazF*-PprgQ-β(M), T-β'(M) (GenScript), respectively, and into *pfo*I/*Xho*I site of pCOLADuet-1 through Gibson assembly kit (New England Biolabs), resulting in pCOLA-PluxI I11-GFP(M)-PprqQ-*mazF*-PprgQ-β(M)-PprgQ-β'(M). Primer sets of Pf_PI11-GFP(M)-PprgQ-mazF(PCOLA)/Pr_PI11-GFP(M)-PprgQ-mazF-EF-Ts(M),

Pf_ PprgQ-EF-Ts(M)/Pr_PprgQ-EF-Ts(M)(PCOLA) were used to amplify GFP(M) with PluxI I11, *mazF* with PprgQ, and EF-Ts(M) with PprgQ from pCOLA-PluxI I11-GFP(M)-PprqQ-*mazF*, T-EF-Ts(M) (GenScript), respectively, and into *pfo*I/*Xho*I site of pCOLADuet-1 through Gibson assembly kit (New England Biolabs), resulting in pCOLA-PluxI I11-GFP(M)-PprqQ-*mazF*-PprgQ-EF-Ts(M). Primer sets of Pf_PI11-GFP(M)-PprgQ-mazF(PCOLA) /Pr_PI11-GFP(M)-PprgQ-mazF-S9(M), Pf_ PprgQ-S9(M)/Pr_PprgQ-S9(M)(PCOLA) were used to amplify GFP(M) with PluxI I11, *mazF* with PprgQ, and S9(M) with PprgQ from pCOLA-PluxI I11-GFP(M)-PprqQ-*mazF*, T-S9(M) (GenScript), respectively, and into *pfo*I/*Xho*I site of pCOLADuet-1 through Gibson assembly kit (New England Biolabs), resulting in pCOLA-PluxI I11-GFP(M)-PprgQ-*mazF*-PprgQ-S9(M). Primer sets of Pf_PI11-GFP(M)-PprgQ-*mazF*(PCOLA) /Pr_PI11-GFP(M)-PprgQ-*mazF*-S20(M), Pf_ PprgQ-S20(M)/Pr_PprgQ-S20(M) were used to amplify GFP(M) with PluxI I11, *mazF* with PprgQ, and S20(M) with PprgQ from pCOLA-PluxI I11-GFP(M)-PprqQ-*mazF*, T-S20(M) (GenScript), respectively, and into *pfo*I/*Xho*I site of pCOLADuet-1 through Gibson assembly kit (New England Biolabs), resulting in pCOLA-PluxI I11-GFP(M)-PprqQ-*mazF*-PprgQ-S20(M). Primer sets of Pf_PI11-GFP(M)-PprgQ-mazF(PCOLA)/Pr_PI11-GFP(M)-PprgQ-mazF, Pf_ PprgQ-L17(M)/Pr_PprgQ-L17(M)(PCOLA) were used to amplify GFP(M) with PluxI I11, *mazF* with PprgQ, and L17(M) with PprgQ from pCOLA-PluxI I11-GFP(M)-PprqQ-*mazF*, T-L17(M) (GenScript), respectively, and into *pfo*I/*Xho*I site of pCOLADuet-1 through Gibson assembly kit (New England Biolabs), resulting

in pCOLA-PluxI I11-GFP(M)-PprqQ-*mazF*-PprgQ-L17(M). Primer sets of Pf_PI11-GFP(M)-PprgQ-mazF(PCOLA)/Pr_PI11-GFP(M)-PprgQ-mazF-PprgQ-EF-Ts- S9(M), Pf_PprgQ-S9(M)/Pr_PprgQ-S9(M)(PCOLA) were used to amplify GFP(M) with PluxI I11, *mazF* with PprgQ, EF-Ts(M) with PprgQ, and S9(M) with PprgQ from pCOLA-PluxI I11-GFP(M)-PprqQ-*mazF*-PprgQ-EF-Ts(M), T-S9(M) (GenScript), respectively, and into *pfo*I/*Xho*I site of pCOLADuet-1 through Gibson assembly kit (New England Biolabs), resulting in pCOLA-PluxI I11-GFP(M)-PprgQ-*mazF*-PprgQ-EF-Ts(M)-PprgQ-S9(M). Primer sets of Pf_PI11-GFP(M)-PprgQ-mazF(PCOLA)/ Pr_PI11-GFP(M)-PprgQ-MazF-PprgQ-EF-Ts(M)-S20(M), Pf_PprgQ-S20(M)/Pr_PprgQ-S20(M)(PCOLA) were used to amplify GFP(M) with PluxI I11, *mazF* with PprgQ, EF-Ts(M) with PprgQ, and S20(M) with PprgQ from pCOLA-PluxI I11-GFP(M)-PprqQ-*mazF*-PprgQ-EF-Ts(M), T-S20(M) (GenScript), respectively, and into *pfo*I/*Xho*I site of pCOLADuet-1 through Gibson assembly kit (New England Biolabs), resulting in pCOLA-PluxI I11-GFP(M)-PprqQ-mazF-PprgQ-EF-Ts(M)-PprgQ-S20(M). Primer sets of Pf_PI11-GFP(M)-PprgQ-mazF(PCOLA)/ Pr_PI11-GFP(M)-PprgQ-mazF-PprgQ-EF-Ts(M)-L17(M), Pf_PprgQ-L17(M)/Pr_ PprgQ-L17(M)(PCOLA) were used to amplify GFP(M) with PluxI I11, *mazF* with PprgQ, EF-Ts(M) with PprgQ, and L17(M) with PprgQ from pCOLA-PluxI I11-GFP(M)-PprqQ-*mazF*-PprgQ-EF-Ts(M), T-L17(M) (GenScript), respectively, and into *pfo*I/*Xho*I site of pCOLADuet-1 through Gibson assembly kit (New England

Biolabs), resulting in pCOLA-PluxI I11-GFP(M)-PprqQ-*mazF*-PprgQ-EF-Ts(M)-PprgQ-L17(M). Primer sets of Pf_PI11-GFP(M)-PprgQ-mazF(PCOLA)/ Pr_PI11-GFP(M)-PprgQ-mazF-PprgQ-EF-Ts(M)-β(M)-β'(M), Pf_ PprgQ-β(M) /Pr_ PprgQ-β'(M)(PCOLA) were used to amplify GFP(M) with PluxI I11, *mazF* with PprgQ, EF-Ts(M) with PprgQ, and β(M) with PprgQ, β'(M) with PprgQ from pCOLA-PluxI I11-GFP(M)-PprqQ-*mazF*-PprgQ-EF-Ts(M), pCOLA-PluxI I11-GFP(M)-PprqQ-*mazF*-PprgQ-β(M)-PprgQ-β'(M) (GenScript), respectively, and into *pfo*I/*Xho*I site of pCOLADuet-1 through Gibson assembly kit (New England Biolabs), resulting in pCOLA-PluxI I11-GFP(M)-PprqQ-*mazF*-PprgQ-EF-Ts(M)-PprgQ-β(M)- PprgQ-β'(M).

Construction of resource allocator circuits to enhancing MCFA production yields

Primers used in this section were shown in Supplementary Table 8. To drive r-BOX cycle by PluxI I11 circuits with different triggering times, primer sets of Pf_PluxI I11-bktB(PCOLA)/Pr_PluxI I11-bktB, Pf_PluxI I11-fadB/Pr_PluxI I11-fadB, Pf_PluxI I11-ter/Pr_PluxI I11-ter, Pf_PluxI I11-ydiI/Pr_PluxI I11-ydiI(PCOLA) were used to amplify *bktB*, *fadB*, *ter*, *ydiI* with PluxI I11, respectively, from pACYC-T7-*bktB*-T7-*fadB*-T7-*ter*-T7-*ydiI* [2] into *pfo*I/*Xho*I site of pCOLADuet-1 through Gibson assembly kit (New England Biolabs), resulting in pCOLA-PluxI I11-*bktB*-PluxI I11-*fadB*-PluxI I11-*ter*- PluxI I11-*ydiI*.

To place PluxI I11 response promoters fused with each pathway enzyme on

pETDuet-1, primer sets of Pf_PluxI I11-bktB(PETD)/Pr_PluxI I11-bktB, Pf_PluxI I11-fadB/Pr_PluxI I11-fadB, Pf_PluxI I11-ter/Pr_PluxI I11-ter, Pf_PluxI I11-ydiI/Pr_PluxI I11-ydiI(PETD) were used to amplify *bktB*, *fadB*, *ter*, *ydiI* with PluxI I11, respectively, from pACYC-T7-*bktB*-T7-*fadB*-T7-*ter*-T7-*ydiI* [2] into *Eco*NI/*Xho*I site of pETDuet-1 through Gibson assembly kit (New England Biolabs), resulting in pETD-PluxI I11-*bktB*-PluxI I11-*fadB*-PluxI I11-*ter*-PluxI I11-*ydiI*.

To replace PluxI I11 with native PluxI promoter, primer sets of Pf_PluxI-bktB(PETD)/Pr_PluxI-bktB, Pf_PluxI-fadB/Pr_PluxI-fadB, Pf_PluxI-ter/Pr_PluxI -ter, Pf_PluxI-ydiI/Pr_PluxI-ydiI(PETD) were used to amplify *bktB*, *fadB*, *ter*, *ydiI* with PluxI, respectively, from pACYC-T7-*bktB*-T7-*fadB*-T7-*ter*-T7-*ydiI* [2] into *Eco*NI/*Xho*I site of pETDuet-1 through Gibson assembly kit (New England Biolabs), resulting in pETD-PluxI-*bktB*-PluxI-*fadB*-PluxI -*ter*-PluxI-*ydiI*.

To remove recognition sites of MazF in MCFA pathway enzymes, modified BktB (BktB(M)), FadB (FadB(M)), Ter (Ter(M)), YdiI (YdiI(M)) without recognition sites of MazF were synthesized by GenScript (Nanjing, China). Primer sets of Pf_PluxI I11-bktB(M)(PETD)/Pr_PluxI I11-bktB(M), Pf_PluxI I11-fadB(M)/Pr_PluxI I11-fadB(M), Pf_PluxI I11-ter(M)/Pr_PluxI I11-ter(M), Pf_PluxI I11-ydiI(M)/Pr_PluxI I11-ydiI(M)(PETD) were used to amplify *bktB*(M), *fadB*(M), *ter*(M), *ydiI*(M) with PluxI I11 from T-*bktB*(M), T-*fadB*(M), T-*ter*(M), T-*ydiI*(M) (GenScript), respectively, and into *Eco*NI/*Xho*I site of pETDuet-1, resulting in pETD-PluxI I11-*bktB*(M)-PluxI I11-*fadB*(M)-PluxI I11-*ter*(M)-PluxI I11-*ydiI*(M).

Primers Pf_PprgQ-mazF(*pfo*I) and Pr_PprgQ-mazF(*Xho*I) were used to amplify *mazF* with PprgQ promoter from *E. coli* genomic DNA into *pfo*I/*Xho*I site of pCOLADuet-1, resulting in pCOLA-PprgQ-*mazF*.

To investigate the effect of protecting host factors from MazF on MCFA titers, primers Pf_PprgQ(MCFA)/Pr_GFP(M)(MCFA) were used to delete GFP with PluxI I11 from pCOLA-PluxI I11-GFP(M)-PprqQ-*mazF*-PprgQ-β(M), pCOLA-PluxI I11-GFP(M)-PprqQ-*mazF*-PprgQ-β'(M), pCOLA-PluxI I11-GFP(M)-PprqQ-*mazF*-PprgQ-β(M)-PprgQ-β'(M), pCOLA-PluxI I11-GFP(M)-PprqQ-*mazF*-PprgQ-EF-Ts(M), pCOLA-PluxI I11-GFP(M)-PprqQ-*mazF*-PprgQ-S9(M), pCOLA-PluxI I11-GFP(M)-PprqQ-*mazF*-PprgQ-S20(M), pCOLA-PluxI I11-GFP(M)-PprqQ-*mazF*-PprgQ-L17(M), pCOLA-PluxI I11-GFP(M)-PprqQ-*mazF*-PprgQ-EF-Ts(M)-PprgQ-S9(M), pCOLA-PluxI I11-GFP(M)-PprqQ-*mazF*-PprgQ-EF-Ts(M)-PprgQ-S20(M), pCOLA-PluxI I11-GFP(M)-PprqQ-*mazF*-PprgQ-EF-Ts(M)-PprgQ-L17(M), pCOLA-PluxI I11-GFP(M)-PprqQ-*mazF*-PprgQ-EF-Ts(M)- PprgQ-β(M)- PprgQ-β'(M), respectively, using PCR-based site-directed mutagenesis (TaKaRa MutanBEST Kit, Takara Biotechnology, Dalian, China), which resulted in pCOLA-PprqQ-*mazF*-PprgQ-β(M), pCOLA-PprqQ-*mazF*-PprgQ-β'(M), pCOLA-PprqQ-*mazF*-PprgQ-β(M)-PprgQ-β'(M), pCOLA-PprqQ-*mazF*-PprgQ-EF-Ts(M), pCOLA-PprqQ-*mazF*-PprgQ-S9(M), pCOLA- PprqQ-*mazF*-PprgQ-S20(M), pCOLA-PprqQ-*mazF*-PprgQ-L17(M), pCOLA- PprqQ-*mazF*-PprgQ-EF-Ts(M)-PprgQ-S9(M), pCOLA-

PprqQ-*mazF*-PprgQ-EF-Ts(M)-PprgQ-S20(M),

pCOLA-PprqQ-*mazF*-PprgQ-EF-Ts(M)-PprgQ-L17(M),

pCOLA-PprqQ-*mazF*-PprgQ-EF-Ts(M)- PprgQ-β(M)- PprgQ-β'(M).

Supplementary results

Identifying orthogonal QS systems functional in a single cell

As engineered QVX circuit from *V. fischeri* and QEX circuit from *E. faecalis* exhibited high dynamic range, low leakiness and completely orthogonal characteristics, these two candidate QS systems were tested to explore whether they could be functional in a single cell. Different reporters such as GFP and mKate2 were placed downstream of PluxI I11 (Supplementary Fig. 3A) and PprgQ (Supplementary Fig. 3B) response promoters, respectively, to examine their behaviors independently. Furthermore, the signal auto-inducers and regulator proteins of QVX and QEX systems were driven by different strength promoters (P1-P6) to trigger these two QS systems at varied times, which would facilitate examining their response functions.

To characterizing QEX system behaviors, PrgX and PrgZ were placed under the control of a constitutive Trc and P1 promoter, respectively, while CcfA was subsequently inserted into a library of different strength promoters (Supplementary Fig. 3B). Generally, as promoter strength driving QS components increased, an increase in peak reporter fluorescence and a decrease in triggering times were observed (Supplementary Fig. 3A and 3B). The relative lower peak GFP fluorescence of QVX P1 compared to QVX P2 (Supplementary Fig. 3A) may be due to the excessively expressed QS components and the resulted unexpected metabolic burden to the cell, as the hampered cellular growth of engineered strains harboring QVX P1 circuit was observed (Supplementary Fig. 2).

These two types of QS systems driven by different strength promoters for QS

component expression were subsequently assembled into a single cell to test their individual functions for the first time (Supplementary Fig. 3C). A predicted rank of triggering times for each QS system was observed and each QS system was only activated at certain times consistent with their corresponding places of QS component expression strengths, demonstrating no crosstalk between these two QS systems in a single cell. The rank of triggering times measured from co-expressed QS systems (Supplementary Fig. 3C) correlated well with previous ranks from singular QS systems (Supplementary Fig. 3A and 3B), only exhibiting a weak delay. This may be due to the extra metabolic burden causing by co-expressing two types of QS systems. Ultimately, two candidate sets of engineered QS circuits with tunable and orthogonal capabilities were identified, allowing implementation of different pathway-specific triggering times to regulate endogenous and engineered pathways, respectively. Representative microscopy images of engineered strains harboring two QS systems in a single cell were shown in Supplementary Fig. 5, further confirmed the proper function of these two QS systems without crosstalk.

Enhancing resource redistribution activity by protecting transcriptional and translational machinery

Regular function of synthetic circuits depends on a dense network of transcriptional and translational machinery and MazF-mediated decay of these factors would disrupt circuit functions. Hence, we sought to examine essential factors that could improve the resource allocator performance after protection from MazF. As PluxI response promoter is a RNA polymerase-dependent promoter [3], we firstly tested

whether protection of RNA polymerase could enhance resource redistribution activity. Since *E. coli* only possesses one RNA polymerase consisting of three different subunits $\alpha_2\beta\beta'$ [4], these three protected subunits α_2(M), β(M), β'(M) were introduced through the same QEX circuit driving MazF expression (Supplementary Fig. 10). The natural subunits $\alpha_2\beta\beta'$ contains 10, 58, 49 MazF sites, respectively (Gene sequences in Supplementary materials). Introduction of α_2(M) had no observable effect on fold change of resource allocator, while introduction of β(M) and β'(M) achieved 14-and 15.4-fold change, respectively, and the combination of β(M) and β'(M) supported a 18.5-fold change (Supplementary Fig. 10).

E. coli basic translation machinery are involved with 78 factors comprising ribosomal proteins and aminoacyl-tRNA synthases, and previous proteome analysis found that three ribosomal protein subunits S9, S20, L17 and elongation factor EF-Ts decreased by approximately 20% and 80%, respectively. Based on these facts, the above-mentioned proteins were protected from MazF to examine resource redistribution activity. Among these candidates, modified EF-Ts (EF-Ts(M)) driven by QEX circuit exhibited maximum fold change of 16.3, while S9(M), S20(M), L17(M) yielded fold change of 13.6, 12.7, 14.6, respectively. However, a combination of EF-Ts(M) and other protected factors produced no observable stacked effect on resource redistribution activity, whereas an additional enhancement of 23.6-fold change was achieved by co-expression of EF-Ts(M) and β(M), β'(M) (Supplementary Fig. 10). Quantitative real-time PCR was conducted to measure mRNA level of protected genes for confirming the protection (Supplementary Fig. 11).

Figures

Supplementary Fig. 1 Schematic of a pathway-independent and full-autonomous global resource allocation strategy in *E. coli*. A pathway-independent dynamic resource allocation strategy, which allows us to control two different cellular states independently, was developed to globally redistribute cellular resources toward synthetic circuits. State 1 was responsible for target compound production controlled by QVX system, while state 2 was responsible for globally degrading competing pathways. QVX system was engineered quorum sensing system from *V. fischeri*, and QEX system was from *E. faecalis*.

433

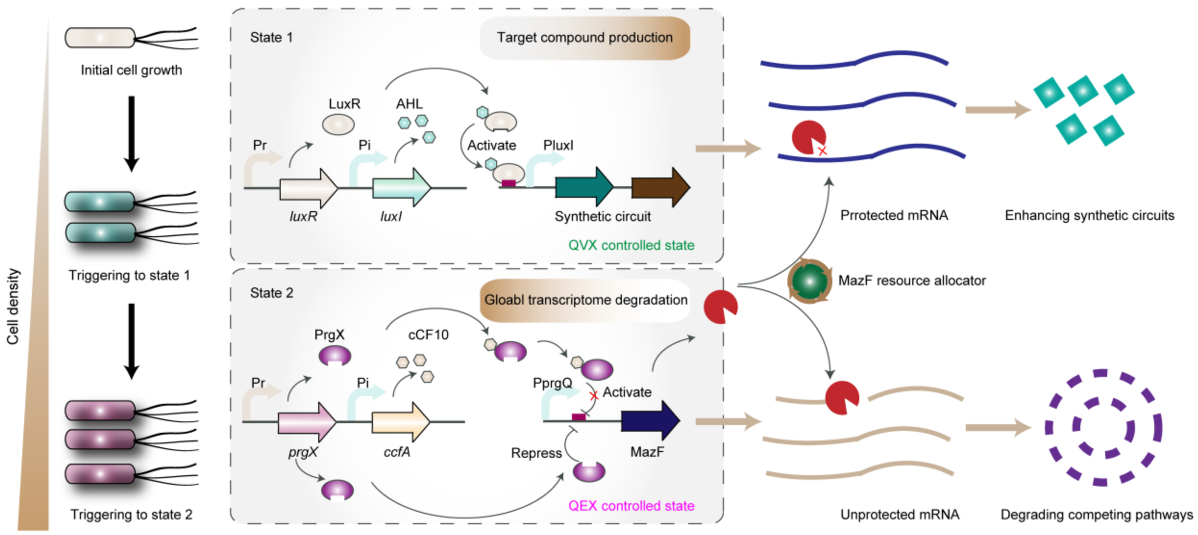

Initial cell growth
Triggering to state 1
Triggering to state 2
Cell density
State 1
Target compound production
LuxR
AHL
Pr
Pi
Activate
PluxI
luxR
luxI
Synthetic circuit
QVX controlled state
State 2
Gloabl transcriptome degradation
PrgX
cCF10
Pr
Pi
PprgQ
Activate
prgX
ccfA
Repress
MazF
QEX controlled state
Prrotected mRNA
Enhancing synthetic circuits
MazF resource allocator
Unprotected mRNA
Degrading competing pathways

Supplementary Fig. 2 Cell growth (final OD_{600}) of engineered strains contained QS components from *Staphylococcus aureus*. This figure showed final OD_{600} of different engineered strains containing part of QS components from *S. aureus*. (-) indicated the wild type strain *E. coli* MG1655 harboring three corresponding empty plasmids as the negative control. The detailed information of modular plasmid systems was presented in Fig. 1A. The recombinant strains were grown in 25 mL of MOPS medium at 30 °C with 220 rpm orbital shaking. Cell density (OD_{600}) was measured after 30 h of culture on a Cytation 3 imaging reader system (BioTek, Winooski, USA). Three biological replicates were performed and averaged for each sample. Values are shown as mean ± SD (n = 3 biological replicates).

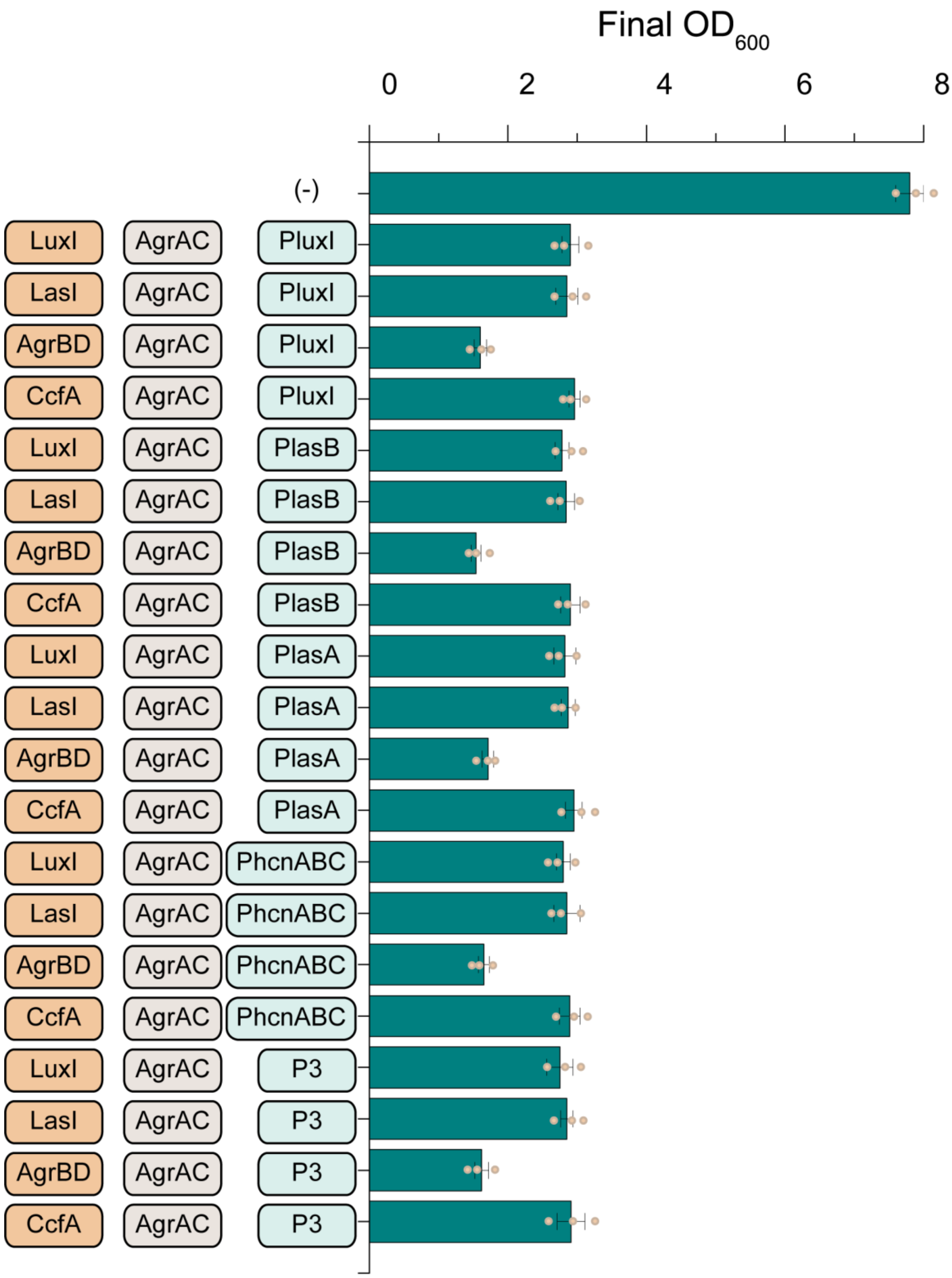
Final OD600
0
2
4
6
8
(-)
LuxI AgrAC PluxI
LasI AgrAC PluxI
AgrBD AgrAC PluxI
CcfA AgrAC PluxI
LuxI AgrAC PlasB
LasI AgrAC PlasB
AgrBD AgrAC PlasB
CcfA AgrAC PlasB
LuxI AgrAC PlasA
LasI AgrAC PlasA
AgrBD AgrAC PlasA
CcfA AgrAC PlasA
LuxI AgrAC PhcnABC
LasI AgrAC PhcnABC
AgrBD AgrAC PhcnABC
CcfA AgrAC PhcnABC
LuxI AgrAC P3
LasI AgrAC P3
AgrBD AgrAC P3
CcfA AgrAC P3

Supplementary Fig. 3 Cell growth (final OD_{600}) of engineered strains used different strength promoters to drive QVX component expression. This figure showed final OD_{600} of different engineered strains using P1-P6 promoters to express LuxR and LuxI. The recombinant strains were grown in 25 mL of MOPS medium at 30 °C with 220 rpm orbital shaking. Cell density (OD_{600}) was measured after 30 h of culture on a Cytation 3 imaging reader system (BioTek, Winooski, USA). Values are shown as mean ± SD (n = 3 biological replicates).

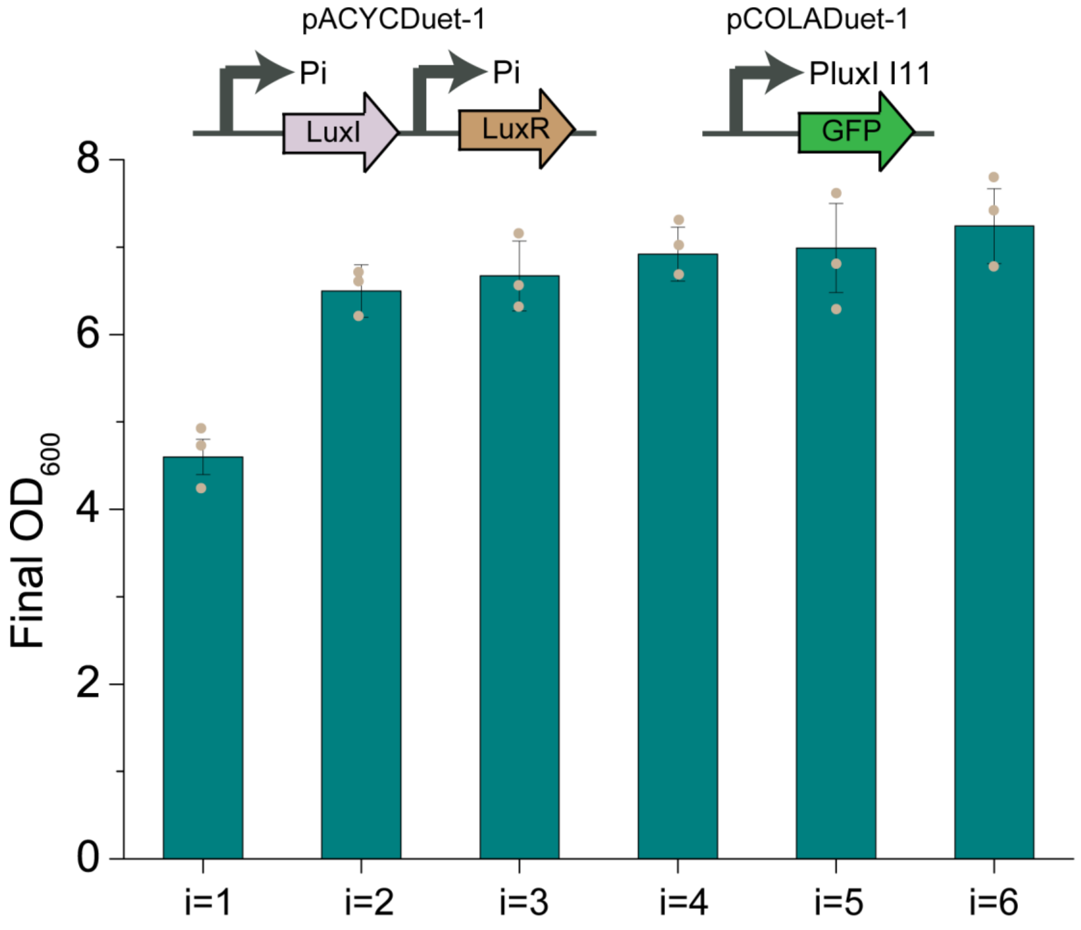
pACYCDuet-1
Pi
LuxI
Pi
LuxR
pCOLADuet-1
PluxI I11
GFP
Final OD600
0
2
4
6
8
i=1
i=2
i=3
i=4
i=5
i=6

Supplementary Fig. 4 Identifying orthogonal QS systems functional in a single cell. (A) Examining individual QVX response behavior with different strength promoters for QS component expression. Response promoter PluxI tagged with GFP was placed on pCOLADuet-1. LuxR and LuxI were expressed under different strength promoters (P1-P6) on pACYCDuet-1. (B) Examining individual QEX response behavior with different strength promoters for CcfA expression. Response promoter PprgQ tagged with mKate2 was placed on pCOLADuet-1. PrgX and PrgZ were placed under P1 and Ptrc constitutive promoter, respectively, on pACYCDuet-1, while CcfA was placed under Pi (i=1-6) promoter on the same plasmid. (C) Assembling QVX and QEX systems into a single cell to test their response functions. For each graph, left coordinates indicated normalized GFP fluorescence, while right one indicated normalized mKate2 fluorescence. All the QS components were placed on pACYCDuet-1, while response promoter PluxI and PprgQ tagged with GFP and mKate2 were placed on pCOLADuet-1. QVX P1 indicated LuxR and LuxI were expressed under P1 promoter, while QEX P1 indicated CcfA was expressed under P1 promoter. Assemble QVX indicated QVX in assemble systems, while single QVX indicated single QS system. Pre-cultured recombinant cells were diluted to OD_{600} of 0.01 in 96-well plate in 200 μL of MOPS medium on an Infinite M1000 PRO (Tecan, Switzerland) plate reader at 30 °C. Cell density and fluorescence were measured every 1 h for 30 h. Values are shown as mean ± SD (n = 3 biological replicates).

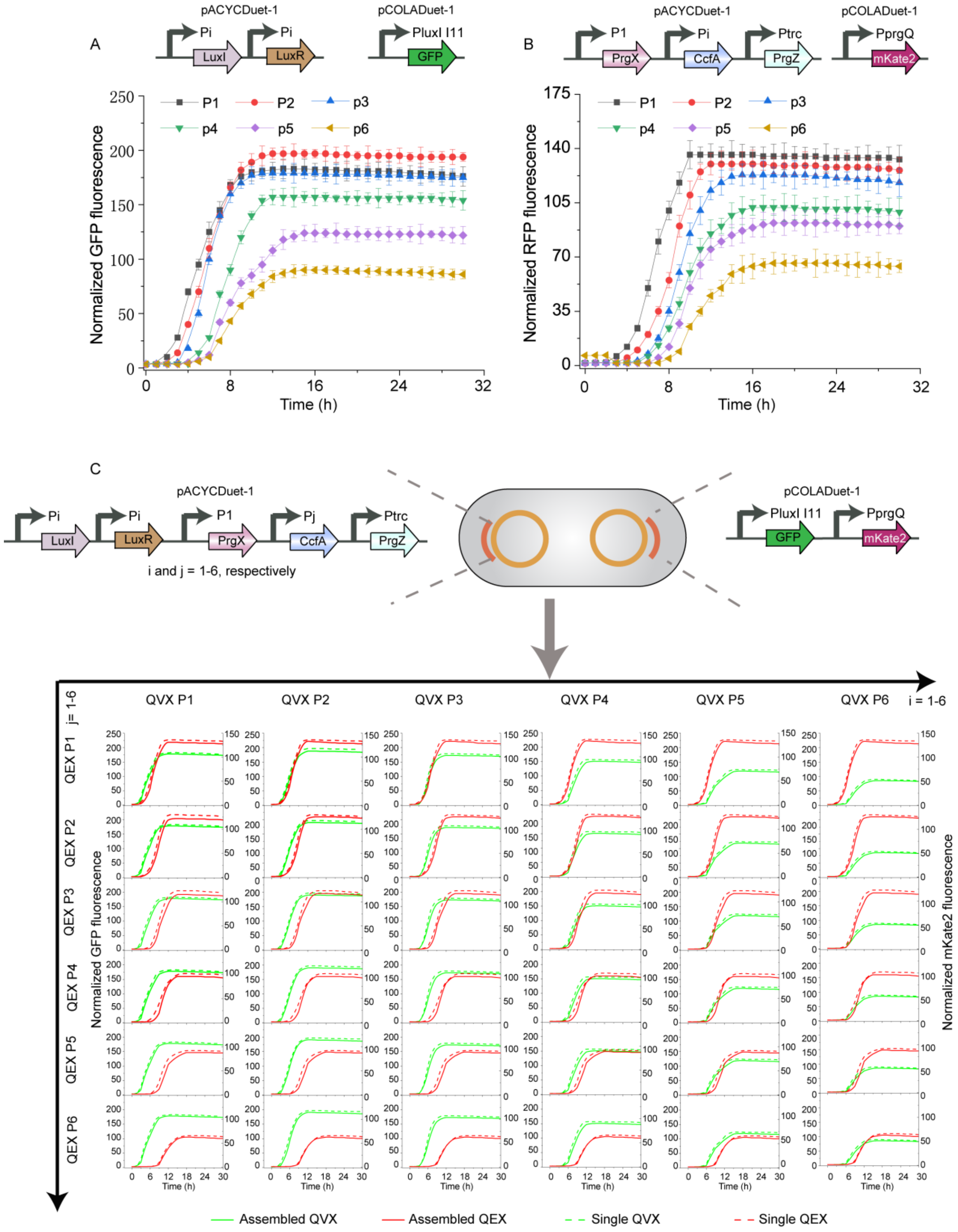
A
pACYCDuet-1
Pi
LuxI
Pi
LuxR
pCOLADuet-1
PluxI I11
GFP
Normalized GFP fluorescence
Time (h)
P1
P2
p3
p4
p5
p6
B
pACYCDuet-1
P1
PrgX
Pi
CcfA
Ptrc
PrgZ
pCOLADuet-1
PprgQ
mKate2
Normalized RFP fluorescence
Time (h)
C
pACYCDuet-1
Pi
LuxI
Pi
LuxR
P1
PrgX
Pj
CcfA
Ptrc
PrgZ
i and j = 1-6, respectively
pCOLADuet-1
PluxI I11
GFP
PprgQ
mKate2
QVX P1
QVX P2
QVX P3
QVX P4
QVX P5
QVX P6
i = 1-6
j= 1-6
QEX P1
QEX P2
QEX P3
QEX P4
QEX P5
QEX P6
Normalized GFP fluorescence
Normalized mKate2 fluorescence
Time (h)
Assembled QVX
Assembled QEX
Single QVX
Single QEX

Supplementary Fig. 5 Representative microscopy images of engineered strains harboring two QS systems in a single cell. (A) Microscopy images of engineered cells harboring QVX P1 and QEX Pi (i=1-6), respectively. QVX P1 indicated using QVX system for driving GFP expression with LuxR and LuxI under P1 promoter, while QEX Pi indicated using QEX system for driving mKate2 expression with CcfA under different strength promoters (P1-P6). Cells after 8 h of culture were photographed using a Nikon Eclipse 80i microscope (Nikon, Tokyo, Japan), as the triggering time for QEX P6 circuit was approximately after 8 h of culture (Fig. 6C). (A) Microscopy images of engineered cells harboring QEX P1 and QVX Pi (i=1-6), respectively. QEX P1 indicated using QEX system for driving mKate2 expression with CcfA under P1 promoter, while QVX Pi indicated using QVX system for driving GFP expression with LuxR and LuxI under different strength promoters (P1-P6). Cells after 6 h of culture were photographed, as the triggering time for QVX P6 circuit was approximately after 6 h of culture (Fig. S4C). For each experiment, three biological replicates were conducted independently with similar results.

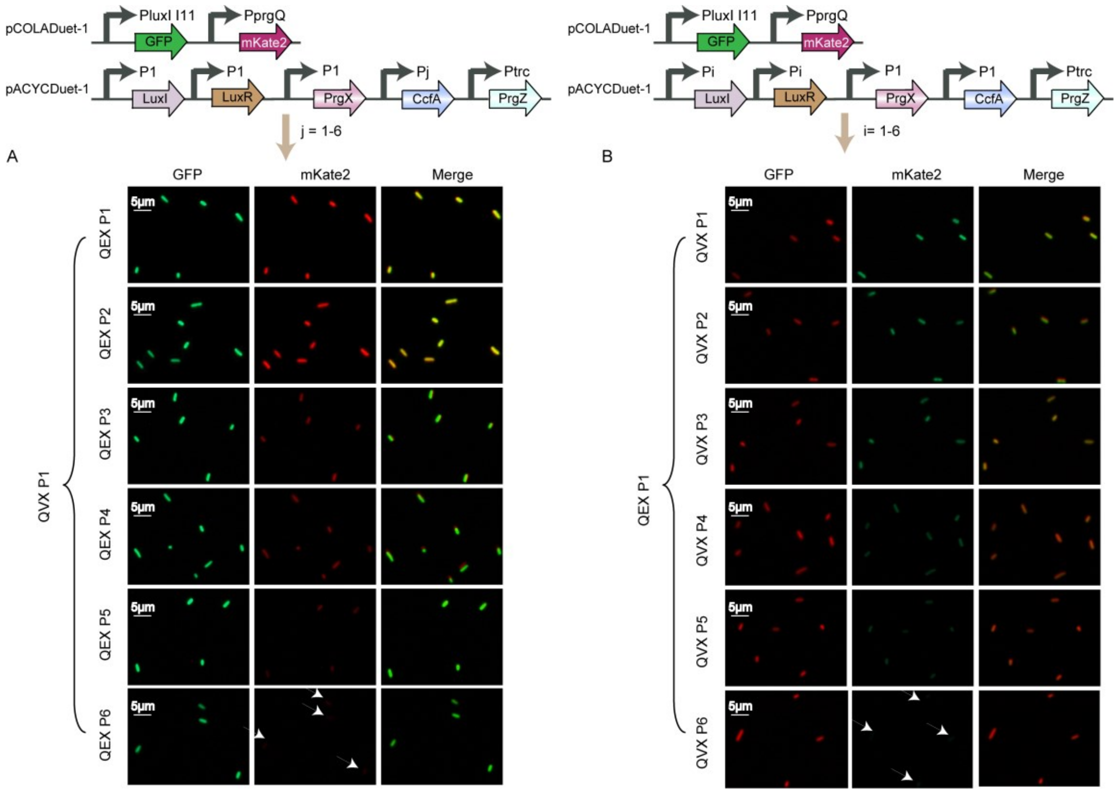
pCOLADuet-1
PluxI I11
GFP
PprgQ
mKate2
pACYCDuet-1
P1
LuxI
P1
LuxR
P1
PrgX
Pj
CcfA
Ptrc
PrgZ
j = 1-6
A
GFP
mKate2
Merge
5μm
QEX P1
QEX P2
QEX P3
QEX P4
QEX P5
QEX P6
QVX P1
pCOLADuet-1
PluxI I11
GFP
PprgQ
mKate2
pACYCDuet-1
Pi
LuxI
Pi
LuxR
P1
PrgX
P1
CcfA
Ptrc
PrgZ
i= 1-6
B
GFP
mKate2
Merge
5μm
QVX P1
QVX P2
QVX P3
QVX P4
QVX P5
QVX P6
QEX P1

Supplementary Fig. 6 The impact of different triggering times of MazF expression on cell growths. This figure presented final OD_{600} of engineered strains with different triggering times of MazF expression. The engineered strains harbored QVX P2 GFP(M) and QEX Pj MazF circuits (j=1-6). QVX P2 GFP(M) indicated GFP(M) driven by QVX circuit using P2 promoter for LuxR and LuxI expression. QEX Pj MazF indicated MazF driven by QEX circuit using Pj promoter for CcfA expression. The recombinant strains were grown in 25 mL of MOPS medium at 30 °C with 220 rpm orbital shaking. Cell density (OD_{600}) was measured after 30 h of culture on a Cytation 3 imaging reader system (BioTek, Winooski, USA). Values are shown as mean ± SD (n = 3 biological replicates).

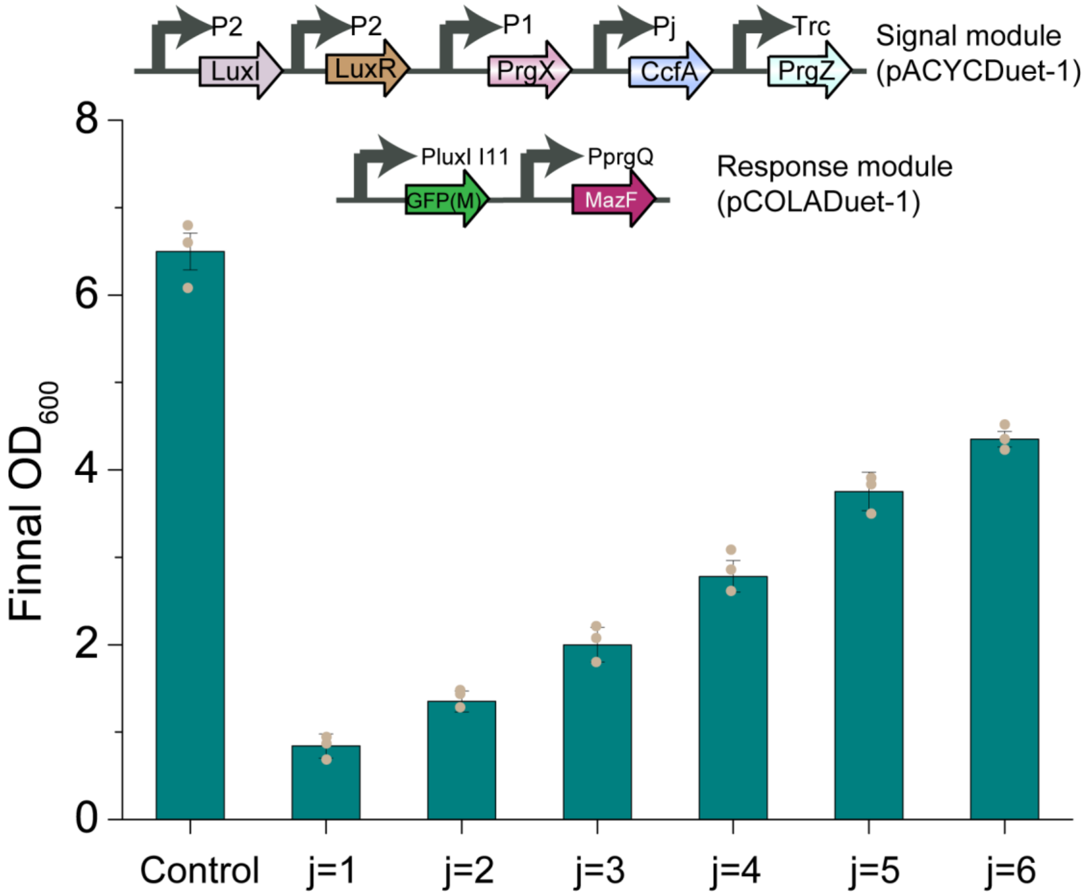
P2
LuxI
P2
LuxR
P1
PrgX
Pj
CcfA
Trc
PrgZ
Signal module
(pACYCDuet-1)
PluxI I11
GFP(M)
PprgQ
MazF
Response module
(pCOLADuet-1)
Finnal OD600
8
6
4
2
0
Control
j=1
j=2
j=3
j=4
j=5
j=6

Supplementary Fig. 7 The impact of MazF expression on fluorescence fold change without protecting GFP. This figure indicated the fluorescence fold change when introduced MazF driven by QEX circuit using Pj promoter for CcfA expression (j=1-6) into control strains harboring QVX Pi GFP(N) circuits (i=1-6). N indicated native GFP without protection from MazF activities. Pre-cultured recombinant cells were diluted to OD_{600} of 0.01 in 96-well plate in 200 μL of MOPS medium on an Infinite M1000 PRO (Tecan, Switzerland) plate reader at 30 °C. The fold change was defined as the ratio of normalized GFP fluorescence in the presence to absence of MazF after 30 h at 30 °C. Values are shown as mean ± SD (n = 3 biological replicates).

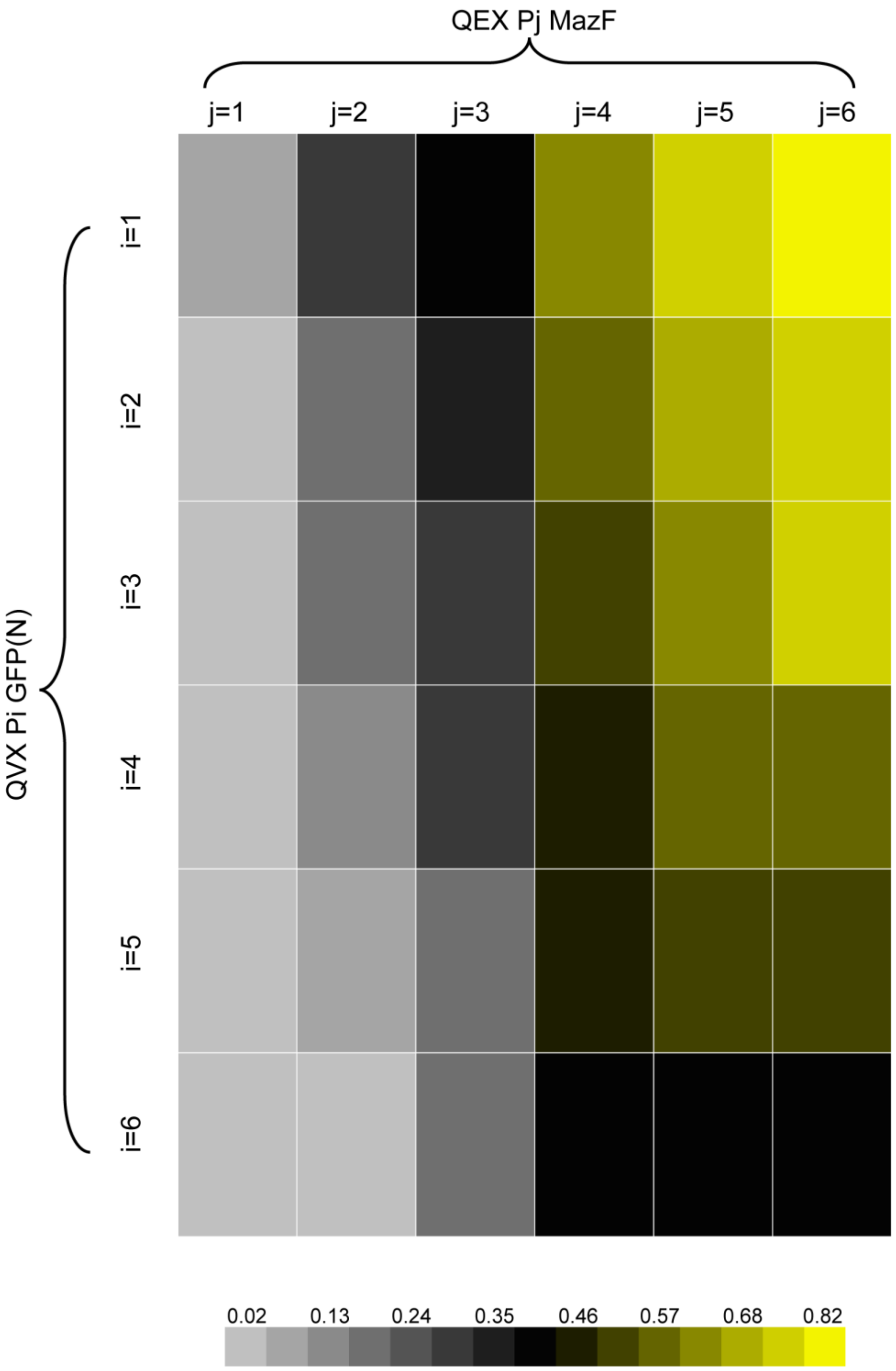
QEX Pj MazF
j=1
j=2
j=3
j=4
j=5
j=6
QVX Pi GFP(N)
i=1
i=2
i=3
i=4
i=5
i=6
0.02
0.13
0.24
0.35
0.46
0.57
0.68
0.82

Supplementary Fig. 8 The impact of protecting MazF on resource redistribution activity. N indicated native MazF, whereas M indicated modified MazF without MazF recognition sites. The recombinant strains were grown in 25 mL of MOPS medium at 30 °C with 220 rpm orbital shaking. Cell density (OD_{600}) and fluorescence were measured after 30 h of culture on a Cytation 3 imaging reader system (BioTek, Winooski, USA). The relative expression levels of *mazF*(N) and *mazF*(M) were confirmed by real-time PCR measurements. Values are shown as mean ± SD (n = 3 biological replicates).

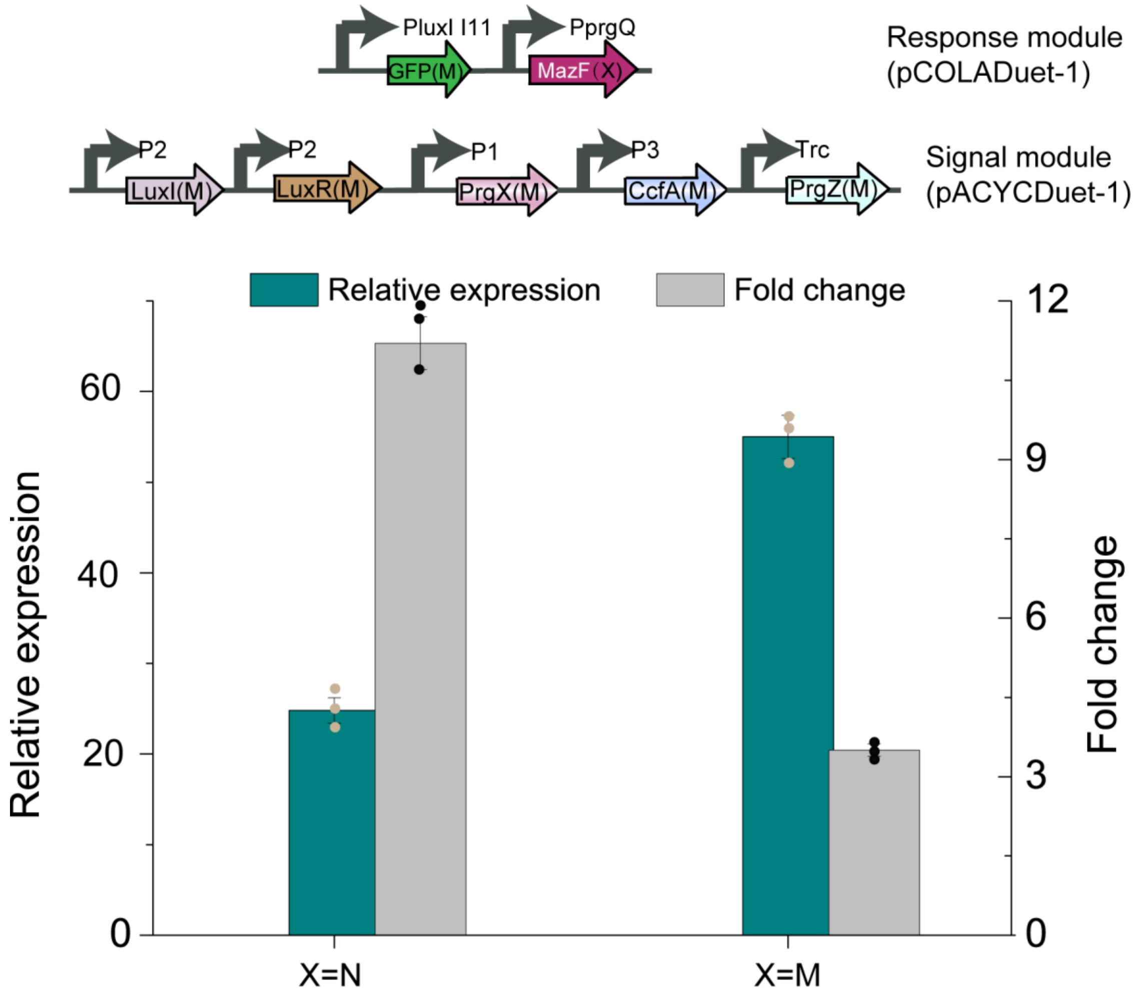
PluxI I11
PprgQ
GFP(M)
MazF (X)
Response module
(pCOLADuet-1)
P2
LuxI(M)
P2
LuxR(M)
P1
PrgX(M)
P3
CcfA(M)
Trc
PrgZ(M)
Signal module
(pACYCDuet-1)
Relative expression
Fold change
Relative expression
Fold change
0
20
40
60
0
3
6
9
12
X=N
X=M

Supplementary Fig. 9 The impact of deleting *mazE* from host genome on resource redistribution activity. 'With *mazE*' indicated wild-type MG1655 harboring QS circuits, whereas 'Without *mazE*' indicated deleting *mazE* from the above strain. The recombinant strains were grown in 25 mL of MOPS medium at 30 °C with 220 rpm orbital shaking. Cell density (OD_{600}) and fluorescence were measured after 30 h of culture on a Cytation 3 imaging reader system (BioTek, Winooski, USA). Values are shown as mean ± SD (n = 3 biological replicates).

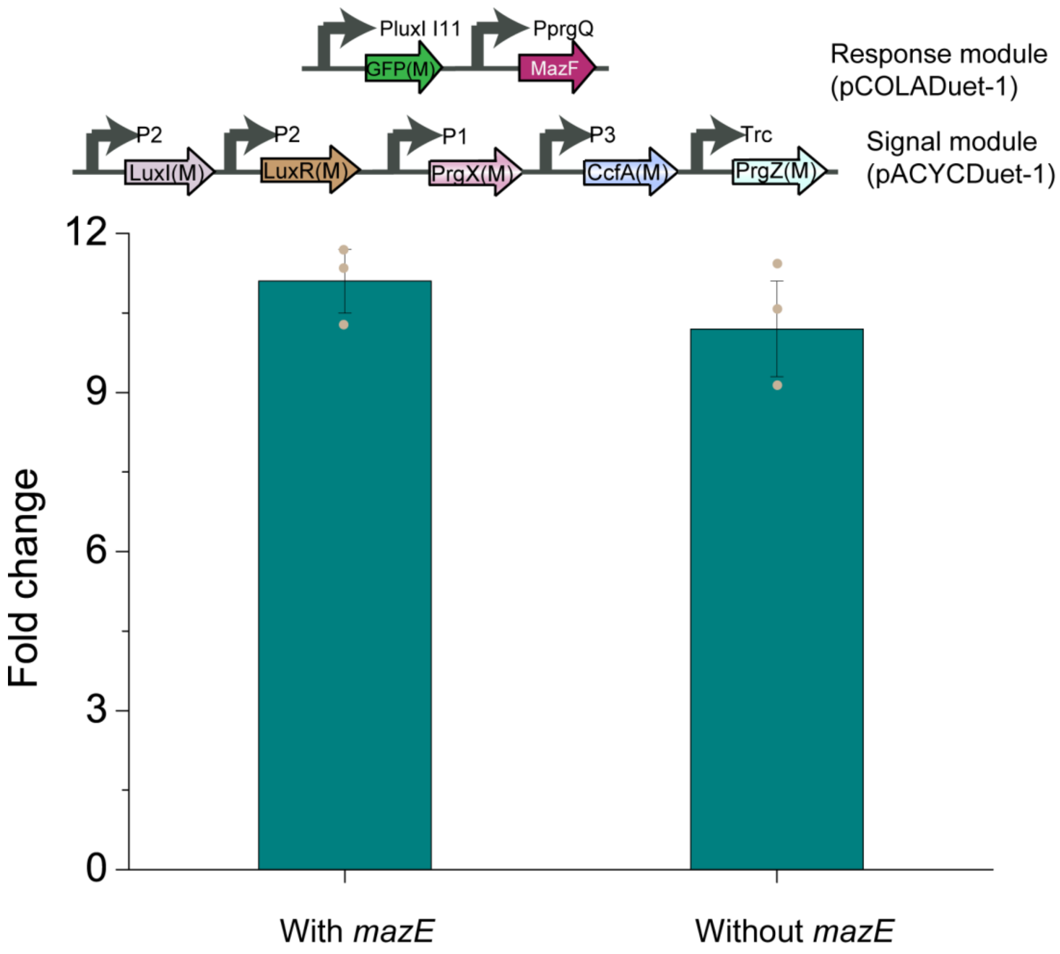
PluxI I11
PprgQ
GFP(M)
MazF
Response module
(pCOLADuet-1)
P2
P2
P1
P3
Trc
LuxI(M)
LuxR(M)
PrgX(M)
CcfA(M)
PrgZ(M)
Signal module
(pACYCDuet-1)
Fold change
12
9
6
3
0
With *mazE*
Without *mazE*

Supplementary Fig. 10 Protecting transcriptional and translational machinery to enhance resource redistribution activity. All the QS components were placed on pACYCDuet-1 as signal module, while the protecting host factors associating with response promoters were placed on pCOLADuet-1 as response module. Pre-cultured recombinant cells were diluted to OD_{600} of 0.01 in 96-well plate in 200 μL of MOPS medium on an Infinite M1000 PRO (Tecan, Switzerland) plate reader at 30 °C. Cellular resource redistribution activity was quantified by fold change defined as the ratio of total GFP fluorescence in the presence to absence of MazF after 30 h at 30 °C. Values are shown as mean ± SD (n = 3 biological replicates).

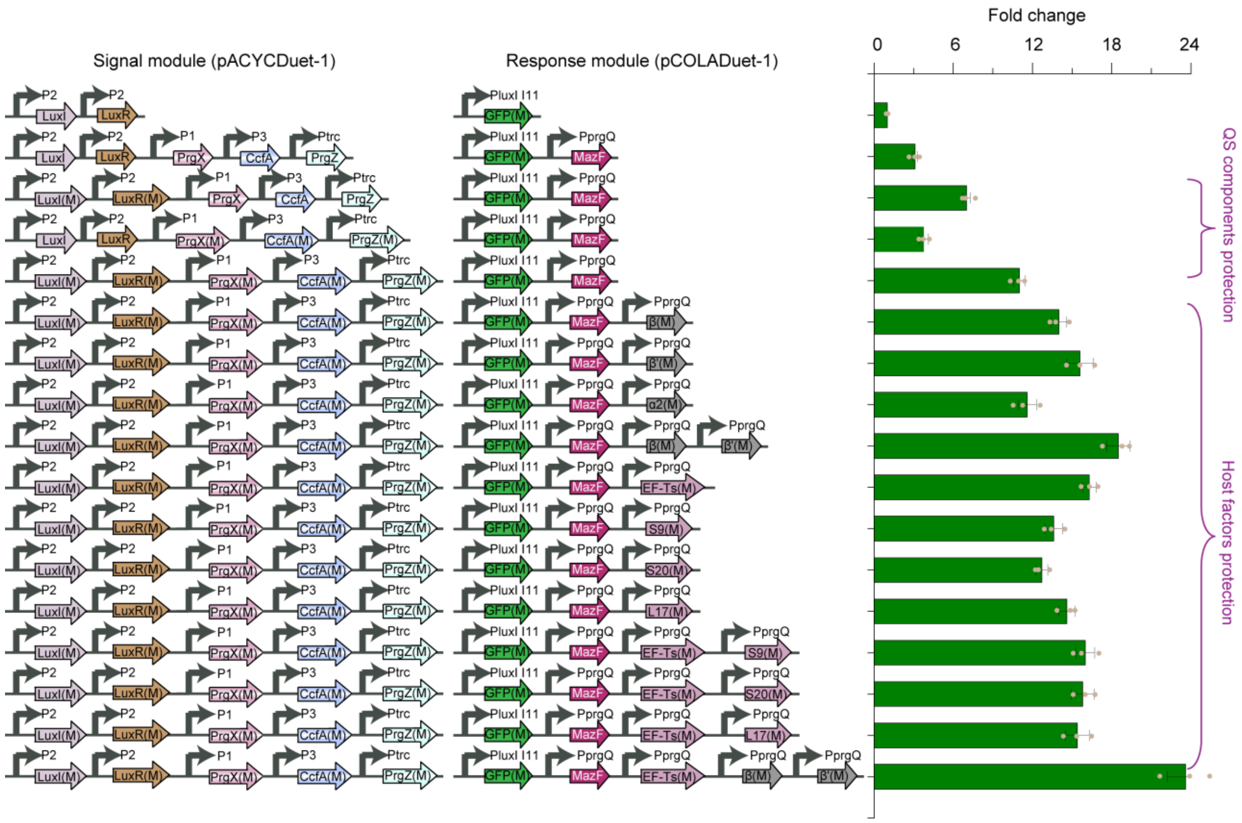
Signal module (pACYCDuet-1)
Response module (pCOLADuet-1)
Fold change
0
6
12
18
24
QS components protection
Host factors protection
P2
LuxI
LuxR
LuxI(M)
LuxR(M)
P1
PrgX
PrgX(M)
P3
CcfA
CcfA(M)
Ptrc
PrgZ
PrgZ(M)
PluxI I11
GFP(M)
PprgQ
MazF
β(M)
β'(M)
α2(M)
EF-Ts(M)
S9(M)
S20(M)
L17(M)

Supplementary Fig. 11 qPCR measurements for transcriptional level analysis to confirm the protection of host factors. qPCR was conducted to measure mRNA level of protected genes for confirming the protection. The left of the figure presented the engineered strains used for qPCR analysis. The recombinant strains were grown in 25 mL of MOPS medium at 30 °C with 220 rpm orbital shaking, and cells were collected at the stationary phase. Values are shown as mean ± SD (n = 3 biological replicates).

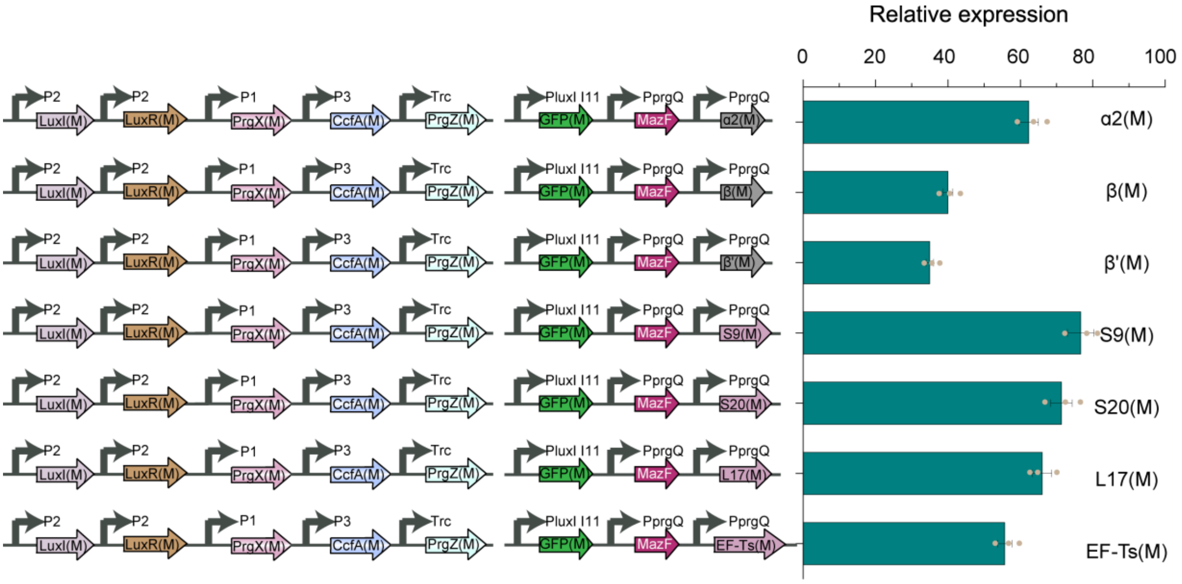
Relative expression
0
20
40
60
80
100
P2
LuxI(M)
P2
LuxR(M)
P1
PrgX(M)
P3
CcfA(M)
Trc
PrgZ(M)
PluxI I11
GFP(M)
PprgQ
MazF
PprgQ
α2(M)
β(M)
β'(M)
S9(M)
S20(M)
L17(M)
EF-Ts(M)

Supplementary Fig. 12 qPCR measurements to confirm pathway enzyme expression levels when using different plasmids of gene copy numbers. qPCR was conducted to measure mRNA levels of pathway enzymes when using pCOLADuet-1 and pETDuet-1 for expression, respectively. The left of the figure presented the engineered strains used for qPCR analysis. The recombinant strains were grown in 50 mL of MOPS medium at 30 °C with 220 rpm orbital shaking, and cells were collected at the stationary phase. Values are shown as mean ± SD (n = 3 biological replicates).

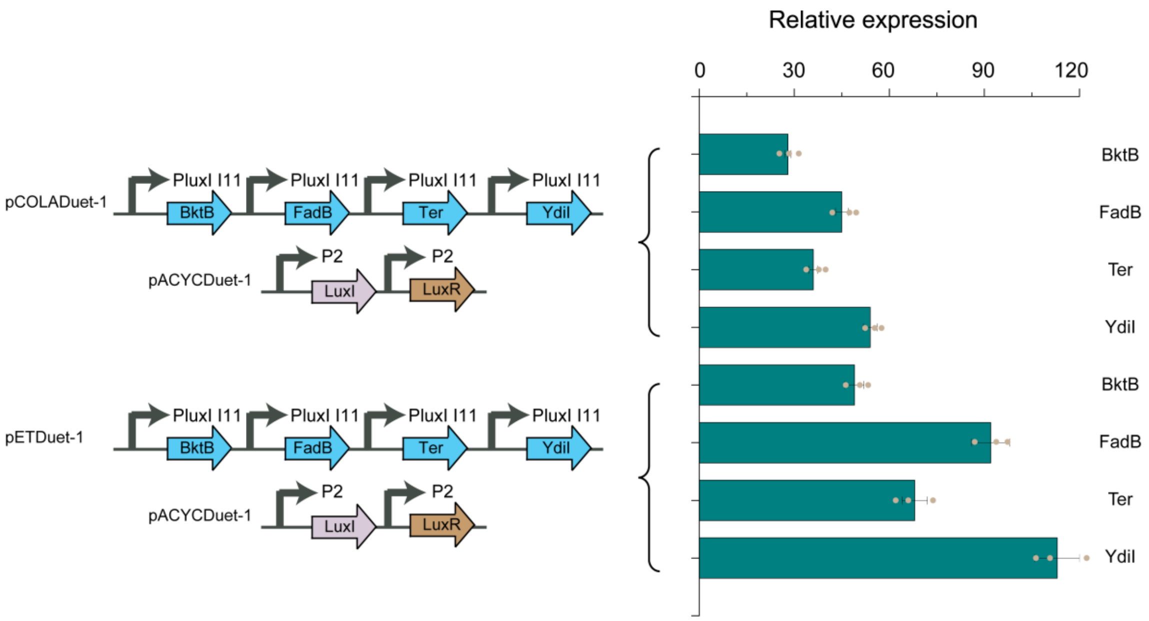
Relative expression
0
30
60
90
120
pCOLADuet-1
PluxI I11
PluxI I11
PluxI I11
PluxI I11
BktB
FadB
Ter
YdiI
pACYCDuet-1
P2
P2
LuxI
LuxR
pETDuet-1
PluxI I11
PluxI I11
PluxI I11
PluxI I11
BktB
FadB
Ter
YdiI
pACYCDuet-1
P2
P2
LuxI
LuxR
BktB
FadB
Ter
YdiI
BktB
FadB
Ter
YdiI

Supplementary Fig. 13 Evaluating resource allocator performance in a 5-L fermenter. (A) The compositions of S_B1, S_B2 and S_B3. S_B1 consisted of pathway module and signal module as control. S_B2 and S_B3 consisted of three modules, while previous identified host factors were protected from MazF on S_B3. (B) Time course of total MCFA production (B1), cell growth (B2), and total glucose consumption (B3) of S_B1, S_B2 and S_B3 in a 5-L fermenter. (C) Time course of four main byproducts accumulation of S_B1 (C1), S_B2 (C2) and S_B3 (C3) in a 5-L fermenter. (D) The comparison of MCFA production among S_B1, S_B2 and S_B3. Values are shown as mean ± SD (n = 3 biological replicates).

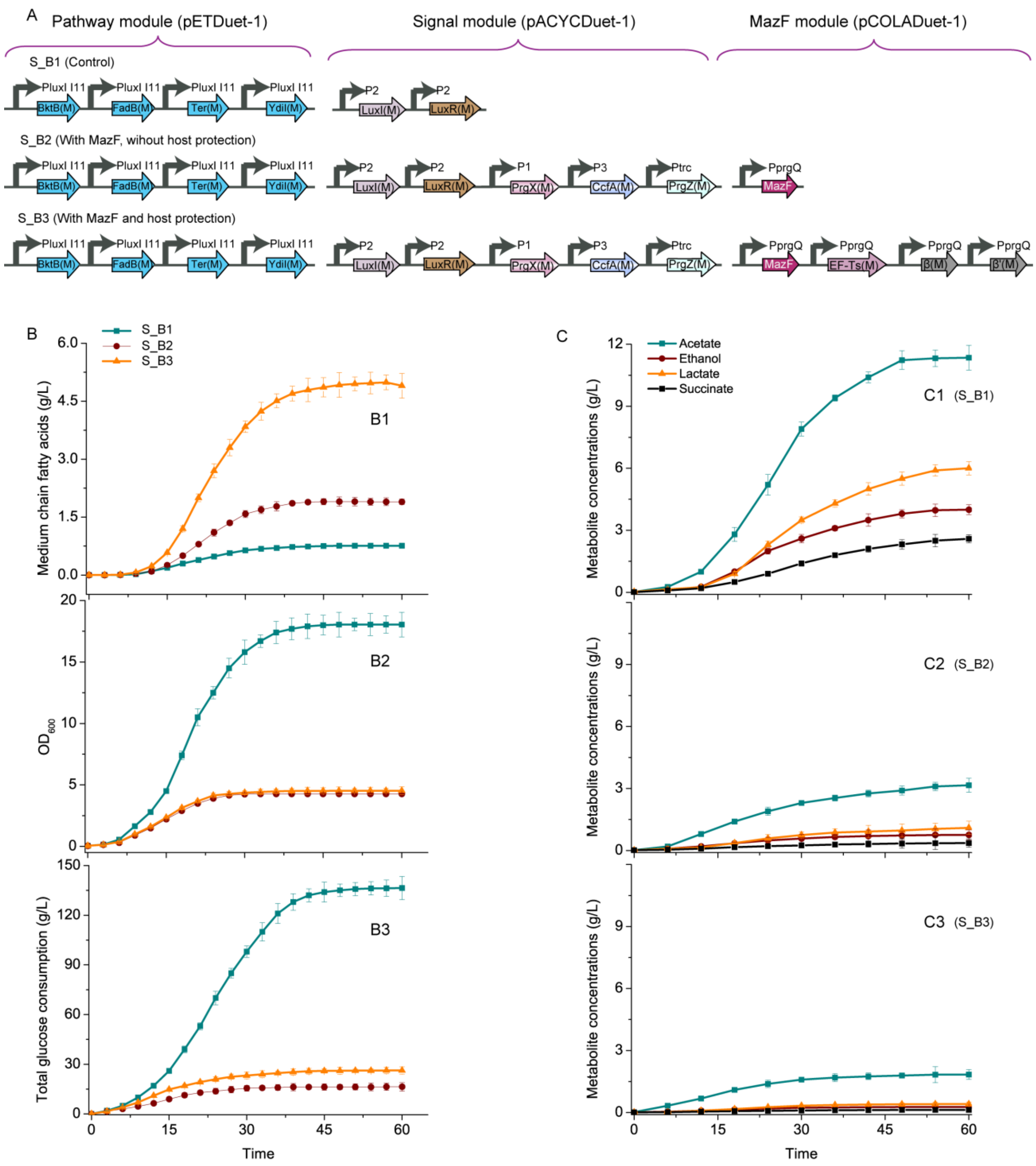

D

Strain	MCFA titer (g/L)	Final OD_{600}	Specific titer (g/L/OD_{600})	Yield (g MCFA/g glucose)
S_B1	0.76	18.06	0.042	0.006
S_B2	1.9	4.31	0.441	0.116
S_B3	4.9	4.5	1.089	0.187

Tables

Supplementary Table 1 Quantitative analysis of microscopic image

	Fluorescence values (a.u.)*									
	Control	I1	I4	I5	I6	I7	I8	I9	I10	I11
Cell 1	21.71	6.97	17.91	18.08	15.58	11.05	5.58	4.56	4.33	3.66
Cell 2	16.21	7.78	17.05	16.27	15.34	10.82	5.46	5.58	4.01	3.84
Cell 3	18.66	8.44	20.63	15.75	20.72	11.46	5.87	5.37	4.28	3.63
Mean	18.86	7.73	18.53	16.71	17.21	11.11	5.64	5.17	4.21	3.71
Error bar	2.76	0.74	1.87	1.22	3.04	0.32	0.21	0.54	0.17	0.11

*a.u. means arbitrary unit.

Supplementary Table 2 Statistical analysis of fluorescence values in different microscopic images. Two sided test was used for data analysis and no adjustments were made for multiple comparisons.

<table>
<tr><th></th><th>Control</th><th>I4</th><th>I5</th><th>I6</th><th>I1</th><th>I7</th><th>I8</th><th>I9</th><th>I10</th><th>I11</th></tr>
<tr><td>P value in different images</td><td colspan="10">0.00006</td></tr>
<tr><td>P value in groups</td><td colspan="4">0.326</td><td colspan="2"></td><td colspan="4">0.423</td></tr>
</table>

Supplementary Table 3 Nucleotide sequences of primers for constructing QS systems

Oligonucleotides	Sequences, 5'-3'[a]
Pf_PluxI-luxI(*Eco*NI)	CCG**CCTGCATTAGG**TTTTTGTTCACCTAGCTTATTGTTAT GTTTTTTGCGTGTTAT
Pr_PluxI-luxI(*Xho*I)	CCG**CTCGAG**TTAATTTGATACAGCTTTTCTAAACT
Pf_PlasI-lasI(*Eco*NI)	CCG**CCTGCATTAGG**TGCTCTGATCTTTTCGGACGTTTCTT CGAGCCTAGCAAGGGT
Pr_PlasI-lasI(*Xho*I)	CCG**CTCGAG**TCATGAAACCGCCAGTCGCTGTTCC
Pf_Pbd-agrBD(*Eco*NI)	CCG**CCTGCATTAGG**CTCATCAACTATTTTCCATCACATCT CTGTGATCTAGTTATATTAAAACA
Pr_Pbd-agrBD(*Xho*I)	CCG**CTCGAG**TTATTCGTGTAATTGAGTTAATTC
Pf_Pc-ccfA(*Eco*NI)	CCG**CCTGCATTAGG**AACGCGGTCACAAGGAATGCTGTGA AGCGGAAAATCCGCGCGAGTTTATT
Pr_Pc-ccfA(*Xho*I)	CCG**CTCGAG**TTATTTTTTCTTCCCTTTTTTC
Pf_PluxR-luxR(*Eco*NI)	CCG**CCTGCATTAGG**TACAGCCATGCAACCTCTCTTATTTT ACATGATCATAACAAACTGATG
Pr_PluxR-luxR(*Xho*I)	CCG**CTCGAG**TTAATTTTTAAGGTATGGACAATTA
Pf_PlasR-lasR(*Eco*NI)	CCG**CCTGCATTAGG**GTGGGACTGAAATGTGCCTTTCCGG CACAACGCCAACTCTATAGAGT
Pr_PlasR-lasR(*Xho*I)	CCG**CTCGAG**TCAGAGAGTAATAAGACCCAAATT
Pf_Pac-agrAC(*Eco*NI)	CCG**CCTGCATTAGG**CTCATCAACTATTTTCCATCACATCT CTGTGATCTAGTTATATTAAAACATGCTAAAAGCATTTAT

	TTTCCAATTTTTCTTAACTAGTCGTTTTTATTCTTAACTGT AAATTTTTTTATGTTAAAATATTAAATACAAATTACATTT AACAGTTAAGTATTTATTTCCTACAGTTAGGCAATATAGT GGAAGCATTAAATGATTATAA
Pr_Pac-agrAC(*Xho*I)	CCG**<u>CTCGAG</u>**TTATATTTTTTTAACGTTTCTCACC
Pf_Pr-prgX(*Eco*NI)	CCG**<u>CCTGCATTAGG</u>**TTACACCCCTCCTATAAAAACATCT TAACATCTAAGTATTATATTTTCAACT
Pr_Pr-prgX(*Xho*I)	CCG**<u>CTCGAG</u>**TCATGACTGCTCTTTTATTTCAGG
Pf_PluxI(*pfo*I)	CATG**<u>TCCGGGA</u>**TTTTTGTTCACCTAGCTTATTGTT
Pr_PluxI(*Xho*I)	CCG**<u>CTCGAG</u>**GGATCCCCATGGTACAGCCATGCAACCTCT CTTATTT
Pf_PlasB(*pfo*I)	CATG**<u>TCCGGGA</u>**GCTGGGGGCAACCTAGCTGCCACCT
Pr_PlasB(*Xho*I)	CCG**<u>CTCGAG</u>**GGATCCCCATGGCTTGTTCAGTTCTCCTGG
Pf_PlasA(*pfo*I)	CATG**<u>TCCGGGA</u>**GCGCGCAGACTGTATCGAAGTATTTC
Pr_PlasA(*Xho*I)	CCG**<u>CTCGAG</u>**GGGTAGCTCCTGGTCATCGAAG
Pf_PhcnABC(*pfo*I)	CATG**<u>TCCGGGA</u>**GTCGGACATGACGGAACGACGGGTG
Pr_PhcnABC(*Xho*I)	CCG**<u>CTCGAG</u>**GGATCCCCATGGTGCCCTTTCATCCGTGAG AGAG
Pf_PprgQ(*pfo*I)	CATG**<u>TCCGGGA</u>**TTTCTTCCTCCTAATATGTAAAGT
Pr_PprgQ(*Xho*I)	CCG**<u>CTCGAG</u>**GGATCCCCATGGTTACACCCCTCCTATAAA AAC
Pf_Pbd-agrB-GSG-GFP	CCG**<u>CCTGCATTAGG</u>**CTATTTTCCATCACATCTCTGTGATC

(*Eco*NI)	T
Pr_Pbd-agrB-GSG-GFP	GTGAAAAGTTCTTCTCCCTTACCCATGCCACTGCCGTCCTCCTTTGAATAGTATATTGGT
Pf_agrB-GSG-GFP	ACCAATATACTATTCAAAGGAGGACGGCAGTGGCATGGGTAAGGGAGAAGAACTTTTCAC
Pr_agrB-GSG-GFP (*Xho*I)	CCG**CTCGAG**TTATTTGTATAGTTCATCCATGCCATG
Pf_Pac-agrC-GSG-GFP (*Eco*NI)	CCG**CCTGCATTAGG**TATTTTCCAATTTTTCTTAACT
Pr_Pac-agrC-GSG-GFP	GTGAAAAGTTCTTCTCCCTTACCCATGCCACTGCCGTTGTTAATAATTTCAACTTTTTG
Pf_agrC-GSG-GFP	CAAAAAGTTGAAATTATTAACAACGGCAGTGGCATGGGTAAGGGAGAAGAACTTTTCAC
Pr_agrC-GSG-GFP (*Xho*I)	CCG**CTCGAG**TTATTTGTATAGTTCATCCATGCCATG
Pf_Pr-prgX-GSG-GFP (*Eco*NI)	CCG**CCTGCATTAGG**TTACACCCCTCCTATAAAAACATCTT
Pr_Pr-prgX-GSG-GFP	GTGAAAAGTTCTTCTCCCTTACCCATGCCACTGCCTGACTGCTCTTTTATTTCAGGAATA
Pf_prgX-GSG-GFP	TATTCCTGAAATAAAAGAGCAGTCAGGCAGTGGCATGGGTAAGGGAGAAGAACTTTTCAC
Pr_prgX-GSG-GFP	CCG**CTCGAG**TTATTTGTATAGTTCATCCATGCCATG

(*Xho*I)	
Pf_Pc-ccfA-GSG-GFP (*Eco*NI)	CCG**CCTGCATTAGG**AACGCGGTCACAAGGAATGCTGTG
Pr_Pc-ccfA-GSG-GFP	GTGAAAAGTTCTTCTCCCTTACCCATGCCACTGCCTTTTTCTTCCCTTTTTTCTTAGGA
Pf_ccfA-GSG-GFP	TCCTAAGAAAAAAGGGAAGAAAAAAGGCAGTGGCATGGGTAAGGGAGAAGAACTTTTCAC
Pr_ccfA-GSG-GFP (*Xho*I)	CCG**CTCGAG**TTATTTGTATAGTTCATCCATGCCATG
Pf_Ptrc-agrB-GSG-GFP (*Eco*NI)	CCG**CCTGCATTAGG***TTGACAATTAATCATCCGGCTCGTATAATGTGTGGTCACACAGG*ATGATAAAAGATTGTACTAAATCGT[j]
Pr_Ptrc-agrB-GSG-GFP	GTGAAAAGTTCTTCTCCCTTACCCATGCCACTGCCGTCCTCCTTTGAATAGTATATTGGT
Pf_Ptrc-agrC-GSG-GFP (*Eco*NI)	CCG**CCTGCATTAGG**TTGACAATTAATCATCCGGCTCGTATAATGTGTGGTCACACAGGGTGGAAGCATTAAATGATTATAAT
Pr_Ptrc-agrC-GSG-GFP	GTGAAAAGTTCTTCTCCCTTACCCATGCCACTGCCGTTGTTAATAATTTCAACTTTTTG
Pf_Ptrc-prgX-GSG-GFP(*Eco*NI)	CCG**CCTGCATTAGG**TTGACAATTAATCATCCGGCTCGTATAATGTGTGGTCACACAGGATGTTTAAGATAGGTTCTGTCCTG

Pr_Ptrc-prgX-GSG-GFP	GTGAAAAGTTCTTCTCCCTTACCCATGCCACTGCCTGACTGCTCTTTTATTTCAGGAATA
Pf_Ptrc-ccfA-GSG-GFP(*Eco*NI)	CCG**CCTGCATTAGG**TTGACAATTAATCATCCGGCTCGTATAATGTGTGGTCACACAGGGTGAAGAAGTATAAGCGCTTATTAT
Pr_Ptrc-ccfA-GSG-GFP	GTGAAAAGTTCTTCTCCCTTACCCATGCCACTGCCTTTTTTCTTCCCTTTTTTCTTAGGA
Pf_Ptrc-luxI(*Eco*NI)	CCG**CCTGCATTAGG**TTGACAATTAATCATCCGGCTCGTATAATGTGTGGTCACACAGGATGATAAAAAAATCGGACTTTTTGG
Pr_Ptrc-luxI(*Xho*I)	CCG**CTCGAG**TTAATTTGATACAGCTTTTC
Pf_Ptrc-lasI(*Eco*NI)	CCG**CCTGCATTAGG**TTGACAATTAATCATCCGGCTCGTATAATGTGTGGTCACACAGGATGATCGTACAAATTGGTCGGCGCG
Pr_Ptrc-lasI(*Xho*I)	CCG**CTCGAG**TCATGAAACCGCCAGTCGCTGTTCCA
Pf_Ptrc-agrBD(*Eco*NI)	CCG**CCTGCATTAGG**TTGACAATTAATCATCCGGCTCGTATAATGTGTGGTCACACAGGATGATAAAAGATTGTACTAAATCG
Pr_Ptrc-agrBD(*Xho*I)	CCG**CTCGAG**TTATTCGTGTAATTGAGTTAATTC
Pf_Ptrc-ccfA(*Eco*NI)	CCG**CCTGCATTAGG**TTGACAATTAATCATCCGGCTCGTATAATGTGTGGTCACACAGGGTGAAGAAGTATAAGCGCTTATTAT

Pr_Ptrc-ccfA(*Xho*I)	CCG**CTCGAG**TTATTTTTTCTTCCCTTTTTTC
Pf_Ptrc-luxR(*Eco*NI)	CCG**CCTGCATTAGG**TTGACAATTAATCATCCGGCTCGTATAATGTGTGGTCACACAGGATGAACATTAAAAATATAAATGCT
Pr_Ptrc-luxR(*Xho*I)	CCG**CTCGAG**TTAATTTTTAAGGTATGGACAATT
Pf_Ptrc-lasR(*Eco*NI)	CCG**CCTGCATTAGG**TTGACAATTAATCATCCGGCTCGTATAATGTGTGGTCACACAGGATGGCCTTGGTTGACGGTTTTCTTG
Pr_Ptrc-lasR(*Xho*I)	CCG**CTCGAG**TCAGAGAGTAATAAGACCCAAATT
Pf_Ptrc-agrAC(*Eco*NI)	CCG**CCTGCATTAGG**TTGACAATTAATCATCCGGCTCGTATAATGTGTGGTCACACAGGGTGGAAGCATTAAATGATTATAATT
Pr_Ptrc-agrAC(*Xho*I)	CCG**CTCGAG**TTATATTTTTTAACGTTTCTCACCG
Pf_Ptrc-prgX(*Eco*NI)	CCG**CCTGCATTAGG**TTGACAATTAATCATCCGGCTCGTATAATGTGTGGTCACACAGGATGTTTAAGATAGGTTCTGTCCTG
Pr_Ptrc-prgX(*Xho*I)	CCG**CTCGAG**TCATGACTGCTCTTTTATTTCAGG

[a]: Bold and underlined letters are restriction enzyme cut sites.

Supplementary Table 4 Nucleotide sequences of primers for generating different variants of PluxI promoters

**Oligonucleotides**	**Sequences, 5'-3'[a]**
Pf_I1-I3	ATGGGTAAGGGAGAAGAACTTTTCACTGGAG
Pr_I1	TCTCTTATTTTACATGATCATAACAAACTG
Pr_I2	TCATAACAAACTGATGCATTACGGAAACCTG
Pr_I3	GAAACCTGTACCATCCTACAGCTGCAA
Pf_I4 (*pfo*I)	CATG**TCCGGGA**ATTGTTATGTTTTTGCGTGTTATAT
Pf_I5 (*pfo*I)	CATG**TCCGGGA**GTGTTATATAACACCAATTTGGAGGTTT
Pf_I6 (*pfo*I)	CATG**TCCGGGA**GGAGGTTTGGTGATATCGCTTCCAATT
Pf_I7 (*pfo*I)	CATG**TCCGGGA**ATCGCTTCCAATTAATTCGATCTGGG
Pf_I8 (*pfo*I)	CATG**TCCGGGA**GGGTCACATTTATGCATCTTGGTGG
Pf_I9 (*pfo*I)	CATG**TCCGGGA**CTTGGTGGAAACGTGGTGTTAACATT
Pf_I10 (*pfo*I)	CATG**TCCGGGA**GTTAACATTGCAGCTGTAGGATGGTACAG
Pf_I11 (*pfo*I)	CATG**TCCGGGA**AGCTGTAGGATGGTACAGGTTTC
Pf_I12 (*pfo*I)	CATG**TCCGGGA**GGTTTCCGTAATGCATCAGTTTGTT
Pr_I4-I12(*Xho*I)	CCG**CTCGAG**TTATTTGTATAGTTCATCCATGCCA
Pf_I9-luxI(*Eco*NI)	CCG**CCTGCATTAGG**CTTGGTGGAAACGTGGTGTTAACATT
Pf_I10-luxI(*Eco*NI)	CCG**CCTGCATTAGG**GTTAACATTGCAGCTGTAGGATGGTACAG

Pf_I11-luxI(*Eco*NI)	CCG**<u>CCTGCATTAGG</u>**AGCTGTAGGATGGTACAGGTTTC
Pr_I9-I11(*Xho*I)	CCG**<u>CTCGAG</u>**TTAATTTGATACAGCTTTTCTAAAC

[a]: Bold and underlined letters are restriction enzyme cut sites.

Supplementary Table 5 Nucleotide sequences of primers for constructing QS components tagged with different strength promoters

**Oligonucleotides**	**Sequences, 5'-3'[a]**
Pf_P1-prgX-GSG-GFP (*Eco*NI)	CCG**CCTGCATTAGG**AAAAAGAGTATTGACTTCGCATCTTTTTGTACCTATAATGTGTGGAATGTTTAAGATAGGTTCTGTCCTG
Pf_P2-prgX-GSG-GFP (*Eco*NI)	CCG**CCTGCATTAGG**AAAAAATTTATTTGCTTATTAATTCATCCGGCTCGTATAATGTGTGGAATGTTTAAGATAGGTTCTGTCCTG
Pf_P3-prgX-GSG-GFP (*Eco*NI)	CCG**CCTGCATTAGG**TTGACAATTAATCATCCGGCTCGTAATTTATGTGGAATGTTTAAGATAGGTTCTGTCCTG
Pf_P4-prgX-GSG-GFP (*Eco*NI)	CCG**CCTGCATTAGG**AAAAAATTTATTTGCTTTCGCATCTTTTTGTACCTATAATGTGTGGAATGTTTAAGATAGGTTCTGTCCTG
Pf_P5-prgX-GSG-GFP (*Eco*NI)	CCG**CCTGCATTAGG**TTGCCTCTTAATCATCGGCTCGTATAATGTGTGGAATGTTTAAGATAGGTTCTGTCCTG
Pf_P6-prgX-GSG-GFP (*Eco*NI)	CCG**CCTGCATTAGG**TTGACAATTAATCATCCGGCTCTTAGTGTTTGTGGAATGTTTAAGATAGGTTCTGTCCTG
Pr_(P1-P6)-prgX-GSG-GFP(*Xho*I)	CCG**CTCGAG**TTATTTGTATAGTTCATCCATGCCATG
Pf_Ptrc-ccfA(G)(pETD)	GACGCTCTCCCTTATGCGACTCCTGCATTAGGGTGAAGAAGTATAAGCGCTTATTAT

Pr_Ptrc-ccfA(G)	ATTATACGAGCCGGATGATTAATTGTCAATTATTTTTCTTCCCTTTTTTCTTAG
Pf_Ptrc-prgZ(G)	GAAAAAAGGGAAGAAAAAATAATTGACAATTAATCATCCGGCTCGTATAATGTGTGGTCACACAGGATGAAGAAGTACAAGAAGTTTTG
Pr_Ptrc-prgZ(G)(PETD)	CGCAGCAGCGGTTTCTTTACCAGACTCGAGTTACTTCTCTATTCTCATTTTTTG
Pf_P1-luxI(G)(PACYC)	ATCTCGACGCTCTCCCTTATGCGACTCCTGCATTAGGAAAAAGAGTATTGACTTCGCATCTTTTTGTACCTATAATGTGTGGAATGATAAAAAAATCGGACTTTTTG
Pr_P1-luxI(G)	TCCACACATTATAGGTACAAAAAGATGCGAAGTCAATACTCTTTTTTTAATTTGATACAGCTTTTCTAA
Pf_P1-luxR(G)	TTAGAAAAGCTGTATCAAATTAAAAAAAGAGTATTGACTTCGCATCTTTTTGTACCTATAATGTGTGGAATGAACATTAAAAATATAAATGCT
Pr_P1-P6-luxR(G)(PACYC)	CGCAGCAGCGGTTTCTTTACCAGACTCGAGTTAATTTTTAAGGTATGGACAATTAAT
Pf_P2-luxI(G)(PACYC)	ATCTCGACGCTCTCCCTTATGCGACTCCTGCATTAGGAAAAAATTTATTTGCTTATTAATTCATCCGGCTCGTATAATGTGTGGAATGATAAAAAAATCGGACTTTTTG
Pr_P2-luxI(G)	TCCACACATTATACGAGCCGGATGAATTAATAAGCAAATAAATTTTTTTTAATTTGATACAGCTTTTCTAA

Pf_P2-luxR(G)	AAAAAATTTATTTGCTTATTAATTCATCCGGCTCGTATAA TGTGTGGAATGAACATTAAAAATATAAATGCT
Pf_P3-luxI(G) (PACYC)	ATCTCGACGCTCTCCCTTATGCGACTCCTGCATTAGGTTG ACAATTAATCATCCGGCTCGTAATTTATGTGGAATGATAA AAAAATCGGACTTTTTG
Pr_P3-luxI(G)	TCCACATAAATTACGAGCCGGATGATTAATTGTCAATTAA TTTGATACAGCTTTTCTAA
Pf_P3-luxR(G)	TTGACAATTAATCATCCGGCTCGTAATTTATGTGGAATGA ACATTAAAAATATAAATGCT
Pf_P4-luxI(G) (PACYC)	ATCTCGACGCTCTCCCTTATGCGACTCCTGCATTAGGAAA AAATTTATTTGCTTTCGCATCTTTTTGTACCTATAATGTGT GGAATGATAAAAAAATCGGACTTTTTG
Pr_P4-luxI(G)	TCCACACATTATAGGTACAAAAAGATGCGAAAGCAAATA AATTTTTTTAATTTGATACAGCTTTTCTAA
Pf_P4-luxR(G)	AAAAAATTTATTTGCTTTCGCATCTTTTTGTACCTATAATG TGTGGAATGAACATTAAAAATATAAATGCT
Pf_P5-luxI(G) (PACYC)	ATCTCGACGCTCTCCCTTATGCGACTCCTGCATTAGGTTG CCTCTTAATCATCGGCTCGTATAATGTGTGGAATGATAAA AAAATCGGACTTTTTG
Pr_P5-luxI(G)	TCCACACATTATACGAGCCGATGATTAAGAGGCAATTAAT TTGATACAGCTTTTCTAA
Pf_P5-luxR(G)	TTGCCTCTTAATCATCGGCTCGTATAATGTGTGGAATGAA

	CATTAAAAATATAAATGCT
Pf_P6-luxI(G)<br>(PACYC)	ATCTCGACGCTCTCCCTTATGCGACTCCTGCATTAGGTTG<br>ACAATTAATCATCCGGCTCTTAGTGTTTGTGGAATGATAA<br>AAAAATCGGACTTTTTG
Pr_P6-luxI(G)	TCCACAAACACTAAGAGCCGGATGATTAATTGTCAATTAA<br>TTTGATACAGCTTTTCTAA
Pf_P6-luxR(G)	TTGACAATTAATCATCCGGCTCTTAGTGTTTGTGGAATGA<br>ACATTAAAAATATAAATGCT
Pf_P1-prgX(G)<br>(PACYC)	ATCTCGACGCTCTCCCTTATGCGACTCCTGCATTAGGAAA<br>AAGAGTATTGACTTCGCATCTTTTTGTACCTATAATGTGT<br>GGAATGTTTAAGATAGGTTCTGTCCTG
Pr_P1-prgX(G)-P1-ccf<br>A	TCCACACATTATAGGTACAAAAAGATGCGAAGTCAATAC<br>TCTTTTTTCATGACTGCTCTTTTATTTCAGG
Pf_P1-ccfA(G)	AAAAAGAGTATTGACTTCGCATCTTTTTGTACCTATAATG<br>TGTGGAGTGAAGAAGTATAAGCGCTTATTAT
Pr_ccfA(G)	CCTGTGTGACCACACATTATACGAGCCGGATGATTAATTG<br>TCAATTATTTTTTCTTCCCTTTTTTCTTAG
Pf_Ptrc-prgZ(G)	CTAAGAAAAAAGGGAAGAAAAAATAATTGACAATTAATC<br>ATCCGGCTCGTATAATGTGTGGTCACACAGGATGAAGAA<br>GTACAAGAAGTTTTG
Pr_Ptrc-prgZ(G)<br>(PACYC)	CGCAGCAGCGGTTTCTTTACCAGACTCGAGTTACTTCTCT<br>ATTCTCATTTTTTTG

Pr_P1-prgX(G)-P2-ccfA	TCCACACATTATACGAGCCGGATGAATTAATAAGCAAATAAATTTTTTTCATGACTGCTCTTTTATTTCAGG
Pf_P2-ccfA(G)	AAAAAATTTATTTGCTTATTAATTCATCCGGCTCGTATAATGTGTGGAGTGAAGAAGTATAAGCGCTTATTAT
Pr_P1-prgX(G)-P3-ccfA	TCCACATAAATTACGAGCCGGATGATTAATTGTCAATCATGACTGCTCTTTTATTTCAGG
Pf_P3-ccfA(G)	TTGACAATTAATCATCCGGCTCGTAATTTATGTGGAGTGAAGAAGTATAAGCGCTTATTAT
Pr_P1-prgX(G)-P4-ccfA	TCCACACATTATAGGTACAAAAAGATGCGAAAGCAAATAAATTTTTTTCATGACTGCTCTTTTATTTCAGG
Pf_P4-ccfA(G)	AAAAAATTTATTTGCTTTCGCATCTTTTTGTACCTATAATGTGTGGAGTGAAGAAGTATAAGCGCTTATTAT
Pr_P1-prgX(G)-P5-ccfA	TCCACACATTATACGAGCCGATGATTAAGAGGCAATCATGACTGCTCTTTTATTTCAGG
Pf_P5-ccfA(G)	TTGCCTCTTAATCATCGGCTCGTATAATGTGTGGAGTGAAGAAGTATAAGCGCTTATTAT
Pr_P6-prgX(G)-P5-ccfA	TCCACAAACACTAAGAGCCGGATGATTAATTGTCAATCATGACTGCTCTTTTATTTCAGG
Pf_P6-ccfA(G)	TTGACAATTAATCATCCGGCTCTTAGTGTTTGTGGAGTGAAGAAGTATAAGCGCTTATTAT
Pf_mKate2(*Nco*I)	CATG**CCATGG**TGAGCGAGCTGATTAAGGAG
Pr_mKate2(*Bam*HI)	CGC**GGATCC**TCATCTGTGCCCCAGTTTGCTAGGGAG

Pf_P1-luxI-P1-luxR(G)(PACYC)	ATCTCGACGCTCTCCCTTATGCGACTCCTGCATTAGGAAAAAGAGTATTGACTTCGCATCTTTTTGTACCTATAATGTGTGGAATGATAAAAAAATCGGACTTTTTG
Pr_P1-P6-luxI-P1-P6-luxR(G)	TCCACACATTATAGGTACAAAAAGATGCGAAGTCAATACTCTTTTTTTAATTTTTAAGGTATGGACAAT
Pf_P1-prgX-P1-P6-ccfA-Ptrc-prgZ(G)	AAAAAGAGTATTGACTTCGCATCTTTTTGTACCTATAATGTGTGGAATGTTTAAGATAGGTTCTGTCCTG
Pr_P1-prgX-P1-P6-ccfA-Ptrc-PrgZ(G)(PACYC)	CGCAGCAGCGGTTTCTTTACCAGACTCGAGTTACTTCTCTATTCTCATTTTTTG
Pf_P2-luxI-P2-luxR(G)(PACYC)	ATCTCGACGCTCTCCCTTATGCGACTCCTGCATTAGGAAAAAATTTATTTGCTTATTAATTCATCCGGCTCGTATAATGTGTGGAATGATAAAAAAATCGGACTTTTTG
Pf_P3-luxI-P3-luxR(G)(PACYC)	ATCTCGACGCTCTCCCTTATGCGACTCCTGCATTAGGTTGACAATTAATCATCCGGCTCGTAATTTATGTGGAATGATAAAAAAATCGGACTTTTTG
Pf_P4-luxI-P4-luxR(G)(PACYC)	ATCTCGACGCTCTCCCTTATGCGACTCCTGCATTAGGAAAAAATTTATTTGCTTTCGCATCTTTTTGTACCTATAATGTGTGGAATGATAAAAAAATCGGACTTTTTG
Pf_P5-luxI-P5-luxR(G)(PACYC)	ATCTCGACGCTCTCCCTTATGCGACTCCTGCATTAGGTTGCCTCTTAATCATCGGCTCGTATAATGTGTGGAATGATAAAAAAATCGGACTTTTTG

Pf_P6-luxI-P6-luxR(G) (PACYC)	ATCTCGACGCTCTCCCTTATGCGACTCCTGCATTAGGTTG ACAATTAATCATCCGGCTCTTAGTGTTTGTGGAATGATAA AAAAATCGGACTTTTTG
Pf_PluxI I11-GFP(G) (PCOLA)	TCCGGGATCTCGACGCTCTCCCTTATGCGACTCCAGCTGT AGGATGGTACAGGTTTC
Pr_PluxI I11-GFP(G)	ATACTTTACATATTAGGAGGAAGAAATTATTTGTATAGTT CATCCATGCCA
Pf_PprgQ-mKate2(G)	TTTCTTCCTCCTAATATGTAAAGTATC
Pr_PprgQ-mKate2(G) (PCOLA)	CGCAGCAGCGGTTTCTTTACCAGACTCGAGTCATCTGTGC CCCAGTTTGCTAGGG

[a]: Bold and underlined letters are restriction enzyme cut sites.

Supplementary Table 6 Nucleotide sequences of primers for constructing global resource allocators

Oligonucleotides	Sequences, 5'-3'[a]
Pf_MazF(*Nco*I)	CATG**CCATGG**TAAGCCGATACGTACCCGATA
Pr_MazF(*Bam*HI)	CGC**GGATCC**CTACCCAATCAGTACGTTAATTTTGG
Pf_PluxII11-GFP-mazF(G)(PCOLA)	TCCGGGATCTCGACGCTCTCCCTTATGCGACTCCAGCTGTAGGATGGTACAGGTTTC
Pr_PluxII11-GFP-mazF	TGATACTTTACATATTAGGAGGAAGAAATTATTTATATAGTTCATCCATGCCATG
Pf_GFP-PprgQ-mazF	TTTCTTCCTCCTAATATGTAAAGTATCAAAAAG
Pr_GFP-PprgQ-MazF(G)(PCOLA)	CGCAGCAGCGGTTTCTTTACCAGACTCGAGCTACCCAATCAGTACGTTAATTTTGGCTTTA
Pf_P2-luxI(M)(G)(PACYC)	ATCTCGACGCTCTCCCTTATGCGACTCCTGCATTAGGAAAAAATTTATTTGCTTATTAATTCATCCGGCTCGTATAATGTGTGGAATGATAAAAAAATCGGACTTTTTGGG
Pr_P2-luxI(M)(G)	TCCACACATTATACGAGCCGGATGAATTAATAAGCAAATAAATTTTTTTAATTTGATACAGCTTTTCTAAAC
Pf_P2-luxR(M)(G)	AGAAAAGCTGTATCAAATTAAAAAAATTTATTTGCTTATTAATTCATCCGGCTCGTATAATGTGTGGAATGAATATTAAAAATATAAATGC
Pr_P2-luxR(M)(G)	CGCAGCAGCGGTTTCTTTACCAGACTCGAGGCAGCAGCG

(PACYC)	GTTTCTTTACCAGA TTAATTTTTAAGGTATGGACAATT
Pf_P1-prgX(M)(G) (PACYC)	ATCTCGACGCTCTCCCTTATGCGACTCCTGCATTAGGAAA AAGAGTATTGACTTCGCATCTTTTTGTACCTATAATGTGT GGAATGTTTAAGATAGGTTCTGTCCTG
Pr_P1-prgX(M)(G)	TCCACATAAATTACGAGCCGGATGATTAATTGTCAATCAT GACTGCTCTTTTATTTCAGG
Pf_P3-ccfA(M)(G)	TTGACAATTAATCATCCGGCTCGTAATTTATGTGGAATGG TTGTGAAGAAGTATAAGCGC
Pr_P3-ccfA(M)(G)	CCTGTGTGACCACACATTATACGAGCCGGATGATTAATTG TCAATAAGAAAAAAGGGAAGAAAAAATAA
Pf_Ptrc-prgZ(M)(G)	TTGACAATTAATCATCCGGCTCGTATAATGTGTGGTCACA CAGG ATGAAGAAGTATAAGAAGTTTTGTTT
Pr_Ptrc-prgZ(M)(G) (PACYC)	CGCAGCAGCGGTTTCTTTACCAGACTCGAGTTACTTCTCT ATTCTCATTTTT
Pf_P2-luxI(M)-P2-luxR (M)(G)(PACYC)	ATCTCGACGCTCTCCCTTATGCGACTCCTGCATTAGGAAA AAATTTATTTGCTTATTAATTCATCCGGCTCGTATAATGTG TGGAATGATAAAAAAATCGGACTTTTTGGG
Pr_P2-luxI(M)-P2-luxR (M)(G)	TCCACACATTATAGGTACAAAAAGATGCGAAGTCAATAC TCTTTTTTTAATTTTTAAGGTATGGACAATT
Pf_P1-prgX-P3-ccfA-Ptrc-prgZ(G)	AAAAAGAGTATTGACTTCGCATCTTTTTGTACCTATAATG TGTGGAATGTTTAAGATAGGTTCTGTCCTG
Pr_P1-prgX-P3-ccfA-	CGCAGCAGCGGTTTCTTTACCAGACTCGAGTTACTTCTCT

Ptrc-prgZ(G)(PACYC)	ATTCTCATTTTTTT
Pf_P2-luxI-P2-luxR(G) (PACYC)	ATCTCGACGCTCTCCCTTATGCGACTCCTGCATTAGGAAA AAATTTATTTGCTTATTAATTCATCCGGCTCGTATAATGTG TGGAATGATAAAAAAATCGGACTTTTTGG
Pr_P2-luxI-P2-luxR(G)	TCCACACATTATAGGTACAAAAAGATGCGAAGTCAATAC TCTTTTTTTAATTTTTAAGGTATGGACAATTA
Pf_P1-prgX(M)-P3-ccf A(M)-Ptrc-prgZ(M)(G)	AAAAAGAGTATTGACTTCGCATCTTTTTGTACCTATAATG TGTGGAATGTTTAAGATAGGTTCTGTCCTGA
Pr_P1-prgX(M)-P3-ccf A(M)-Ptrc-prgZ(M)(G) (PACYC)	CGCAGCAGCGGTTTCTTTACCAGACTCGAGTTACTTCTCT ATTCTCATTTTTT

[a]: Bold and underlined letters are restriction enzyme cut sites.

Supplementary Table 7 Nucleotide sequences of primers for protecting host factors including transcriptional and translational machinery

**Oligonucleotides**	**Sequences, 5'-3'**
Pf_PI11-GFP(M)-PprgQ-mazF(PCOLA)	TCCGGGATCTCGACGCTCTCCCTTATGCGACTCCAGCTGTAGGATGGTACAGGTTTC
Pr_PI11-GFP(M)-PprgQ-mazF	AAAATTAAAATATTAACAAATTTTTGTAGTTTATCGAATTTTAGGTCTACTTTTTGATACTTTACATATTAGGAGGAAGAAA CTACCCAATCAGTACGTTAATTTTGG
Pf_PprgQ-β(M)	TTTCTTCCTCCTAATATGTAAAGTATCAAAAAGTAGACCTAAAATTCGATAAACTACAAAAATTTGTTAATATTTTAATTTTAGGTATTGAATACGACACTCGAAGATGTGTTTATTAAGCTATATCCCTTTTTTTTAAAAAAAAATACATATTTTAGTTGAAAATATAATACTTAGATGTTAAGATGTTTTTATAGGAGGGGTGTAAATGGTTTACTCCTATACCGAGAAAAAAC
Pr_PprgQ-β(M)(PCOLA)	CGCAGCAGCGGTTTCTTTACCAGACTCGAGTTACTCGTCTTCCAGTTCGATATTGAT
Pf_PprgQ-β'(M)	TTTCTTCCTCCTAATATGTAAAGTATCAAAAAGTAGACCTAAAATTCGATAAACTACAAAAATTTGTTAATATTTTAATTTTAGGTATTGAATACGACACTCGAAGATGTGTTTATTAAGCTATATCCCTTTTTTTTAAAAAAAAATACATATTTTAGTTGAAAATATAATACTTAGATGTTAAGATGTTTTTATAGGAGGGGTGTAAGTGAAAGATTTATTAAAGTTTCTG

Pr_PprgQ-β'(M) (PCOLA)	CGCAGCAGCGGTTTCTTTACCAGACTCGAGTTACTCGTTATCAGAACCGCCCAG
Pf_PprgQ-α2(M)	TTTCTTCCTCCTAATATGTAAAGTATCAAAAAGTAGACCTAAAATTCGATAAACTACAAAAATTTGTTAATATTTTAATTTTAGGTATTGAATACGACACTCGAAGATGTGTTTATTAAGCTATATCCCTTTTTTTTAAAAAAAAATACATATTTTAGTTGAAAATATAATACTTAGATGTTAAGATGTTTTTATAGGAGGGGTGTAAATGCAGGGTTCTGTGACCGAGTTT
Pr_PprgQ-α2(M) (PCOLA)	CGCAGCAGCGGTTTCTTTACCAGACTCGAGTTACTCGTCAGCGATGCTTGCCGGTGGCC
Pr_PI11-GFP(M)-PprgQ-mazF-β(M)-β'(M)	AAAATTAAAATATTAACAAATTTTTGTAGTTTATCGAATTTTAGGTCTACTTTTTGATACTTTACATATTAGGAGGAAGAAA TTACTCGTCTTCCAGTTCGATATTGAT
Pr_PI11-GFP(M)-PprgQ-mazF-EF-Ts(M)	AAAATTAAAATATTAACAAATTTTTGTAGTTTATCGAATTTTAGGTCTACTTTTTGATACTTTACATATTAGGAGGAAGAAA CTACCCAATCAGTACGTTAATTTTGG
Pf_ PprgQ-EF-Ts(M)	TTTCTTCCTCCTAATATGTAAAGTATCAAAAAGTAGACCTAAAATTCGATAAACTACAAAAATTTGTTAATATTTTAATTTTAGGTATTGAATACGACACTCGAAGATGTGTTTATTAAGCTATATCCCTTTTTTTTAAAAAAAAATACATATTTTAGTTGAAAATATAATACTTAGATGTTAAGATGTTTTTATAGGAGGGGTGTAAATGGCTGAAATTACCGCATCCCTGG

Pr_PprgQ-EF-Ts(M) (PCOLA)	CGCAGCAGCGGTTTCTTTACCAGACTCGAGTTAAGACTGC TTGGACATCGCAGC
Pr_PI11-GFP(M)-Pprg Q-mazF-S9(M)	AAAATTAAAATATTAACAAATTTTTGTAGTTTATCGAATT TTAGGTCTACTTTTTGATACTTTACATATTAGGAGGAAGA AA CTACCCAATCAGTACGTTAATTTTGG
Pf_ PprgQ-S9(M)	TTTCTTCCTCCTAATATGTAAAGTATCAAAAAGTAGACCT AAAATTCGATAAACTACAAAAATTTGTTAATATTTTAATT TTAGGTATTGAATACGACACTCGAAGATGTGTTTATTAAG CTATATCCCTTTTTTTTAAAAAAAAATACATATTTTAGTTG AAAATATAATACTTAGATGTTAAGATGTTTTTATAGGAGG GGTGTAAATGGCTGAAAATCAATACTACGGCAC
Pr_PprgQ-S9(M) (PCOLA)	CGCAGCAGCGGTTTCTTTACCAGACTCGAGTTAACGTTTG GAGAACTGCGGAC
Pr_PI11-GFP(M)-Pprg Q-mazF-S20(M)	AAAATTAAAATATTAACAAATTTTTGTAGTTTATCGAATT TTAGGTCTACTTTTTGATACTTTACATATTAGGAGGAAGA AA CTACCCAATCAGTACGTTAATTTTGG
Pf_ PprgQ-S20(M)	TTTCTTCCTCCTAATATGTAAAGTATCAAAAAGTAGACCT AAAATTCGATAAACTACAAAAATTTGTTAATATTTTAATT TTAGGTATTGAATACGACACTCGAAGATGTGTTTATTAAG CTATATCCCTTTTTTTTAAAAAAAAATACATATTTTAGTTG AAAATATAATACTTAGATGTTAAGATGTTTTTATAGGAGG GGTGTAAATGGCTAATATCAAATCAGCTAAG

Pr_PprgQ-S20(M)	CGCAGCAGCGGTTTCTTTACCAGACTCGAGTTAAGCCAGT TTATTGATCTGCGC
Pr_PI11-GFP(M)-Pprg Q-mazF-L17(M)	AAAATTAAAATATTAACAAATTTTTGTAGTTTATCGAATT TTAGGTCTACTTTTTGATACTTTACATATTAGGAGGAAGA AA CTACCCAATCAGTACGTTAATTTTGG
Pf_ PprgQ-L17(M)	TTTCTTCCTCCTAATATGTAAAGTATCAAAAAGTAGACCT AAAATTCGATAAACTACAAAAATTTGTTAATATTTTAATT TTAGGTATTGAATACGACACTCGAAGATGTGTTTATTAAG CTATATCCCTTTTTTTTAAAAAAAAATACATATTTTAGTTG AAAATATAATACTTAGATGTTAAGATGTTTTTATAGGAGG GGTGTAAATGCGCCATCGTAAGAGTGGTCGTC
Pr_PprgQ-L17(M) (PCOLA)	CGCAGCAGCGGTTTCTTTACCAGACTCGAGTTACTCTGCA GCAGCTTCTGCTTT
Pr_PI11-GFP(M)-Pprg Q-mazF-PprgQ-EF-Ts(M)-S9(M)	AAAATTAAAATATTAACAAATTTTTGTAGTTTATCGAATT TTAGGTCTACTTTTTGATACTTTACATATTAGGAGGAAGA AA TTAAGACTGCTTGGACATCGCAGC
Pr_PI11-GFP(M)-Pprg Q-mazF-PprgQ-EF-Ts(M)-S20(M)	AAAATTAAAATATTAACAAATTTTTGTAGTTTATCGAATT TTAGGTCTACTTTTTGATACTTTACATATTAGGAGGAAGA AA TTAAGACTGCTTGGACATCGCAGC
Pr_PI11-GFP(M)-Pprg Q-mazF-PprgQ-EF-Ts(M)-L17(M)	AAAATTAAAATATTAACAAATTTTTGTAGTTTATCGAATT TTAGGTCTACTTTTTGATACTTTACATATTAGGAGGAAGA AA TTAAGACTGCTTGGACATCGCAGC

Pr_PI11-GFP(M)-PprgQ-mazF-PprgQ-EF-Ts(M)-β(M)-β'(M)	AAAATTAAAATATTAACAAATTTTTGTAGTTTATCGAATTTTAGGTCTACTTTTTGATACTTTACATATTAGGAGGAAGAAA TTAAGACTGCTTGGACATCGCAGC

Supplementary Table 8 Nucleotide sequences of primers for constructing resource allocator circuits to enhancing MCFA production yields

**Oligonucleotides**	**Sequences, 5'-3'[a]**
Pf_PluxI	TCCGGGATCTCGACGCTCTCCCTTATGCGACTCCAGCTGT
I11-bktB(PCOLA)	AGGATGGTACAGGTTTCCGTAATGCATCAGTTTGTTATGATCATGTAAAATAAGAGAGGTTGCATGGCTGTAATGACCCGTGAAGTTGTTGTTGTTT
Pr_PluxI I11-bktB	CATTACGGAAACCTGTACCATCCTACAGCTTTAGATACGTTCGAAGATAGCAGCG
Pf_PluxI I11-fadB	TTCGAACGTATCTAAAGCTGTAGGATGGTACAGGTTTCCGTAATGCATCAGTTTGTTATGATCATGTAAAATAAGAGAGGTTGCATGGCTGTAATGCTTTACAAAGGCGACACCCTGT
Pr_PluxI I11-fadB	CATTACGGAAACCTGTACCATCCTACAGCTTTAAGCCGTTTTCAGGTCGCCAACCGG
Pf_PluxI I11-ter	GCGACCTGAAAACGGCTTAAAGCTGTAGGATGGTACAGGTTTCCGTAATGCATCAGTTTGTTATGATCATGTAAAATAAGAGAGGTTGCATGGCTGTAATGTTCACCACCACCGCTAAAGTT
Pr_PluxI I11-ter	CATTACGGAAACCTGTACCATCCTACAGCTTTACTGCTGAGCAGCAGACGGC
Pf_PluxI I11-ydiI	GCCGTCTGCTGCTCAGCAGTAAAGCTGTAGGATGGTACAGGTTTCCGTAATGCATCAGTTTGTTATGATCATGTAAAAT

	AAGAGAGGTTGCATGGCTGTAATGATATGGAAACGGAAA
	ATCACCC
Pr_PluxI	CGCAGCAGCGGTTTCTTTACCAGACTCGAGTCACAAAATG
I11-ydiI(PCOLA)	GCGGTCGTCAATC
Pf_PluxI	GACGCTCTCCCTTATGCGACTCCTGCATTAGGAGCTGTAG
I11-bktB(PETD)	GATGGTACAGGTTTCCGTAATGCATCAGTTTGTTATGATC
	ATGTAAAATAAGAGAGGTTGCATGGCTGTAATGACCCGT
	GAAGTTGTTGTTGTTT
Pr_PluxI	CGCAGCAGCGGTTTCTTTACCAGACTCGAGTCACAAAATG
I11-ydiI(PETD)	GCGGTCGTCAATC
Pf_PluxI-bktB(PETD)	GACGCTCTCCCTTATGCGACTCCTGCATTAGGTTTTTGTTC
	ACCTAGCTTATTGTTATGTTTTTGCGTGTTATATAACACC
	AATTTGGAGGTTTGGTGATATCGCTTCCAATTAATTCGAT
	CTGGGTCACATTTATGCATCTTGGTGGAAACGTGGTGTTA
	ACATTGCAGCTGTAGGATGGTACAGGTTTCCGTAATGCAT
	CAGTTTGTTATGATCATGTAAAATAAGAGAGGTTGCATGG
	CTGTAATGACCCGTGAAGTTGTTGTTGTTT
Pr_PluxI-bktB	ATAACAATAAGCTAGGTGAACAAAAATTAGATACGTTCG
	AAGATAGCAGCG
Pf_PluxI-fadB	TTCGAACGTATCTAATTTTTGTTCACCTAGCTTATTGTTAT
	GTTTTTTGCGTGTTATATAACACCAATTTGGAGGTTTGGT
	GATATCGCTTCCAATTAATTCGATCTGGGTCACATTTATG

	CATCTTGGTGGAAACGTGGTGTTAACATTGCAGCTGTAGG ATGGTACAGGTTTCCGTAATGCATCAGTTTGTTATGATCA TGTAAAATAAGAGAGGTTGCATGGCTGTAATGCTTTACAA AGGCGACACCCTGT
Pr_PluxI-fadB	ATAACAATAAGCTAGGTGAACAAAAATTAAGCCGTTTTC AGGTCGCCAACC
Pf_PluxI-ter	GCGACCTGAAAACGGCTTAATTTTTGTTCACCTAGCTTAT TGTTATGTTTTTTGCGTGTTATATAACACCAATTTGGAGGT TTGGTGATATCGCTTCCAATTAATTCGATCTGGGTCACAT TTATGCATCTTGGTGGAAACGTGGTGTTAACATTGCAGCT GTAGGATGGTACAGGTTTCCGTAATGCATCAGTTTGTTAT GATCATGTAAAATAAGAGAGGTTGCATGGCTGTAATGTTC ACCACCACCGCTAAAGTT
Pr_PluxI -ter	ATAACAATAAGCTAGGTGAACAAAAATTACTGCTGAGCA GCAGACGGC
Pf_PluxI-ydiI	GCCGTCTGCTGCTCAGCAGTAATTTTTGTTCACCTAGCTTA TTGTTATGTTTTTTGCGTGTTATATAACACCAATTTGGAGG TTTGGTGATATCGCTTCCAATTAATTCGATCTGGGTCACA TTTATGCATCTTGGTGGAAACGTGGTGTTAACATTGCAGC TGTAGGATGGTACAGGTTTCCGTAATGCATCAGTTTGTTA TGATCATGTAAAATAAGAGAGGTTGCATGGCTGTAATGA TATGGAAACGGAAAATCACCC

Pr_PluxI-ydiI(PETD)	CGCAGCAGCGGTTTCTTTACCAGACTCGAGTCACAAAATG GCGGTCGTCAATC
Pf_PluxI I11-bktB(M)(PETD)	GACGCTCTCCCTTATGCGACTCCTGCATTAGGAGCTGTAG GATGGTACAGGTTTCCGTAATGCATCAGTTTGTTATGATC ATGTAAAATAAGAGAGGTTGCATGGCTGTAATGACCCGT GAAGTTGTTGTTGTTT
Pr_PluxI I11-bktB(M)	CATTACGGAAACCTGTACCATCCTACAGCTTTAGATACGT TCGAAGATAGCAGCG
Pf_PluxI I11-fadB(M)	TGCTATCTTCGAACGTATCTAAAGCTGTAGGATGGTACAG GTTTCCGTAATGCATCAGTTTGTTATGATCATGTAAAATA AGAGAGGTTGCATGGCTGTAATGCTTTATAAAGGCGATA CCCTG
Pr_PluxI I11-fadB(M)	CATTACGGAAACCTGTACCATCCTACAGCTTTAAGCCGTT TTCAGGTCGCCAACC
Pf_PluxI I11-ter(M)	TTCGAACGTATCTAAAGCTGTAGGATGGTACAGGTTTCCG TAATGCATCAGTTTGTTATGATCATGTAAAATAAGAGAGG TTGCATGGCTGTAATGTTCACCACCACCGCTAAAGTTAT
Pr_PluxI I11-ter(M)	CATTACGGAAACCTGTACCATCCTACAGCTTTACTGCTGA GCAGCAGACGGC
Pf_PluxI I11-ydiI(M)	GCCGTCTGCTGCTCAGCAGTAAAGCTGTAGGATGGTACA GGTTTCCGTAATGCATCAGTTTGTTATGATCATGTAAAAT AAGAGAGGTTGCATGGCTGTAATGATATGGAAACGGAAA

	ATC
Pr_PluxI I11-ydiI(M)(PETD)	CGCAGCAGCGGTTTCTTTACCAGACTCGAGTCACAAAATGGCGGTCGTCAA
Pf_PprgQ-mazF(*Eco*NI)	CATG**TCCGGGA**TTTCTTCCTCCTAATATGTAAAGTATCAAAAAGTAGACCTAAAATTCGATAAACTACAAAAATTTGTTAATATTTTAATTTTAGGTATTGAATACGACACTCGAAGATGTGTTTATTAAGCTATATCCCTTTTTTTTAAAAAAAAATACATATTTTAGTTGAAAATATAATACTTAGATGTTAAGATGTTTTTATAGGAGGGGTGTAAATGGTAAGCCGATACGTACCCGATAT
Pr_PprgQ-mazF(*Xho*I)	CCG**CTCGAG**CTACCCAATCAGTACGTTAATTTTGG
Pf_PprgQ(MCFA)	TTTCTTCCTCCTAATATGTAAAGTATCAAAAAGTAGACCTAAAATTCGATAAACTACAAAAATTTGTTAATATTTTAATT
Pr_GFP(M)(MCFA)	TCCCGGACACCATCGAATGGCGCAAAACCTTTCGCGGTATGGCATGA

[a]: Bold and underlined letters are restriction enzyme cut sites.

Supplementary Table 9 Nucleotide sequences of primers for real-time PCR measurements and gene deletions

**Oligonucleotides**	**Sequences, 5'-3'**
Pf_qα2(M)	CAGGGTTCTGTGACCGAGTT
Pr_qα2(M)	CTTCCTGAACGCCTTCTTTG
Pf_qβ(M)	GTATCCGGGTGAAGCAGGTA
Pr_qβ(M)	TCGGATACGAGGATGGAGTC
Pf_qβ'(M)	GCGTAAAAAGCTGACCAAGC
Pr_qβ'(M)	CAGATCCAGCAGACGTTTCA
Pf_qEF-Ts(M)	TACCGCATCCCTGGTAAAAG
Pr_qEF-Ts(M)	CGTCTTTTGCAACGAAGTCA
Pf_qS9(M)	GGGCAACGGTAAAATCGTAA
Pr_qS9(M)	CAGTTCAGAACGCAGGGACT
Pf_qS20(M)	GCTCGTAAGCATAACGCAAG
Pr_qS20(M)	AGCCTTATGACGTGCAGCTT
Pf_qL17(M)	GAGCCGCTGATTACTCTTGC
Pr_qL17(M)	CGGAAGCCACACTTCAGAAT
Pf_qbktB	TGGTTGTTCGTGAAGCTCTG
Pr_qbktB	GAGCAGCAGAAACGATAGCC
Pf_qfadB	GCATTGCCGAACTGGTATTT
Pr_qfadB	ATCAGCACCGACGATAAAGG
Pf_qter	ACCGTTAACACCGACAAAGC

Pr_qter	TCCAGTATACCGGCCAAGTC
Pf_qydiI	GACACCCTTGAAGCGACAAT
Pr_qydiI	AATCTGCCAGACCTGGTGAC
Pf_cysG	GTGGTCTACGACCGTCTGGT
Pr_cysG	TTGCACAGTGTTTCCAGCTC
Pf_mazF-Kan^{FRT}	GAACTGGTCAACGACATCACGCCGGAAAACCTCCACGAG AATATCGACTGGGGAGAGCCGAAAGATAAGGAAGTCTGG TAGAAGTTCCTATACTTTCTAGAGAATAGGAACTTCGGAA TAGGAACTTC
Pr_mazF-Kan^{FRT}	TTTGATTCATTGAATTGTCCTGAAAATTGCGGGTCTGTCA GGTGGAAACCTGTGACCAGAATAGAAGTGAGTTAGTAAC ACGAAGTTCCTATTCTCTAGAAAGTATAGGAACTTC
Pf_mazE-Kan^{FRT}	TAGGCCGAAATTTGCTCGTATCTACAATGTAGATTGATAT ATACTGTATCTACATATGATAGCGGTTTGAGGAAAGGGTT GAAGTTCCTATACTTTCTAGAGAATAGGAACTTCGGAATA GGAACTTC
Pr_mazE-Kan^{FRT}	AATAGTGAGCAAACGGTCGATTTGATTCATTGAATTGTCC TGAAAATTGCGGGTCTGTCAGGTGGAAACCTGTGACCAG AATAGAAGTGAGTTAGTAACACGAAGTTCCTATTCTCTAG AAAGTATAGGAACTTC

Supplementary Table 10 Plasmids used in this study

Plasmids	Description	Source or reference
pETDuet-1	Double T7 promoters, pBR322 ori, Amp^{R}	Novagen
pCDFDuet-1	Double T7 promoters, CDF ori, Sm^{R}	Novagen
pACYCDuet-1	Double T7 promoters, p15A ori, Cm^{R}	Novagen
pRSFDuet-1	Double T7 promoters, RSF ori, Kn^{R}	Novagen
pCOLADuet-1	Double T7 promoters, COLA ori, Kn^{R}	Novagen
pACYC-T7-*bktB*-T7-*fadB*-T7-*ter*-T7-*ydiI*	pACYCDuet-1 carrying *bktB*, *fadB*, *ter* and *ydiI* under T7 promoter	[2]
pETD-PluxI-*luxI*	pETDuet-1 carrying *luxI* under native PluxI promoter	This study
pETD-PlasI-*lasI*	pETDuet-1 carrying *lasI* under native PlasI promoter	This study
pETD-Pbd-*agrBD*	pETDuet-1 carrying *agrBD* under native Pbd promoter	This study
pETD-Pc-*ccfA*	pETDuet-1 carrying *ccfA* under native Pc promoter	This study
pACYC-PluxR-*luxR*	pACYCDuet-1 carrying *luxR* under native PluxR promoter	This study
pACYC-PlasR-*lasR*	pACYCDuet-1 carrying *lasR* under	This study

	native PlasR promoter	
pACYC-Pac-*agrAC*	pACYCDuet-1 carrying *agrAC* under native Pac promoter	This study
pACYC-Pr-*prgX*	pACYCDuet-1 carrying *prgX* under native Pr promoter	This study
pCOLA-PluxI	pCOLADuet-1 carrying response promoter PluxI	This study
pCOLA-PlasB	pCOLADuet-1 carrying response promoter PlasB	[2]
pCOLA-PlasA	pCOLADuet-1 carrying response promoter PlasA	[2]
pCOLA-PhcnABC	pCOLADuet-1 carrying response promoter PhcnABC	This study
pCOLA-PprgQ	pCOLADuet-1 carrying response promoter PprgQ	This study
pCOLA-PluxI-GFP	pCOLADuet-1 carrying GFP under response promoter PluxI	This study
pCOLA-PlasB-GFP	pCOLADuet-1 carrying GFP under response promoter PlasB	This study
pCOLA-PlasA-GFP	pCOLADuet-1 carrying GFP under response promoter PlasA	This study
pCOLA-PhcnABC-GFP	pCOLADuet-1 carrying GFP under	This study

	response promoter PhcnABC	
pCOLA-PprgQ-GFP	pCOLADuet-1 carrying GFP under response promoter PprgQ	This study
pACYC-Pbd-*agrB*-GSG-GFP	pACYCDuet-1 carrying Pbd-*agrB*, the three amino acid linker, and GFP	This study
pACYC-Pac-*agrC*-GSG-GFP	pACYCDuet-1 carrying Pac-*agrC*, the three amino acid linker, and GFP	This study
pACYC-Pr-*prgX*-GSG-GFP	pACYCDuet-1 carrying Pr-*prgX*, the three amino acid linker, and GFP	This study
pACYC-Pc-*ccfA*-GSG-GFP	pACYCDuet-1 carrying Pc-*ccfA*, the three amino acid linker, and GFP	This study
pACYC-Ptrc-*agrB*-GSG-GFP	pACYCDuet-1 carrying Ptrc-*agrB*, the three amino acid linker, and GFP	This study
pACYC-Ptrc-*agrC*-GSG-GFP	pACYCDuet-1 carrying Ptrc-*agrC*, the three amino acid linker, and GFP	This study
pACYC-Ptrc-*prgX*-GSG-GFP	pACYCDuet-1 carrying Ptrc-*prgX*, the three amino acid linker, and GFP	This study
pACYC-Ptrc-*ccfA*-GSG-GFP	pACYCDuet-1 carrying Ptrc-*ccfA*, the three amino acid linker, and GFP	This study
pACYC-Ptrc-GFP	pACYCDuet-1 carrying Ptrc-GFP	This study
pETD-Ptrc-*luxI*	pETDuet-1 carrying *luxI* under Ptrc promoter	This study

pETD-Ptrc-*lasI*	pETDuet-1 carrying *lasI* under Ptrc promoter	This study
pETD-Ptrc-*agrBD*	pETDuet-1 carrying *agrBD* under Ptrc promoter	This study
pETD-Ptrc-*ccfA*	pETDuet-1 carrying *ccfA* under Ptrc promoter	This study
pACYC-Ptrc-*luxR*	pACYCDuet-1 carrying *luxR* under Ptrc promoter	This study
pACYC-Ptrc-*lasR*	pACYCDuet-1 carrying *lasR* under Ptrc promoter	This study
pACYC-Ptrc-*agrAC*	pACYCDuet-1 carrying *agrAC* under Ptrc promoter	This study
pACYC-Ptrc-*prgX*	pACYCDuet-1 carrying *prgX* under Ptrc promoter	This study
pCOLA-PluxI Ii-GFP (i=1-12)	pCOLADuet-1 carrying GFP under engineered response promoter PluxI Ii	This study
pETD-PluxI Ii-*luxI* (i=9-11)	pETDuet-1 carrying *luxI* under engineered response promoter PluxI Ii	This study
pACYC-Pi-*prgX*-GSG-GFP (i=1-6)	pACYCDuet-1 carrying *prgX* under Pi promoter, the three amino acid linker, and GFP	This study
pCDFD-P1-*prgX*-GSG-GFP	pCDFDuet-1 carrying *prgX* under P1	This study

	promoter, the three amino acid linker, and GFP	
pETD-Ptrc-*ccfA*-Ptrc-*prgZ*	pETDuet-1 carrying *ccfA*, *prgZ* under Ptrc promoter, respectively	This study
pACYC-Pi-*luxI*-Pi-*luxR* (i=1-6)	pACYCDuet-1 carrying *luxI*, *luxR* under Pi promoter, respectively	This study
pACYC-P1-*prgX*-Pi-*ccfA*-Ptrc-*prgZ* (i=1-6)	pACYCDuet-1 carrying *prgX* with P1 promoter, *ccfA* with Pi promoter, *prgZ* with Ptrc promoter	This study
pCOLA-PprgQ-mKate2	pCOLADuet-1 carrying mKate2 under response promoter PprgQ	This study
pACYC-Pi-*luxI*-Pi-*luxR*-P1-*prgX*-Pj-*ccfA*-Ptrc-*prgZ* (i and j=1-6, respectively)	pACYCDuet-1 carrying *luxI* and *luxR* with Pi promoter, *prgX* with P1, *ccfA* with Pj, *prgZ* with Ptrc promoter	This study
pCOLA-PluxI I11-GFP-PprqQ-mKate2	pCOLADuet-1 carrying GFP, mKate2 under response promoter PluxI I11 and PprgQ, respectively	This study
pCOLA-PluxI I11-GFP(M)	pCOLADuet-1 carrying GFP(M) under response promoter PluxI I11	This study
pCOLA-PprgQ-*mazF*	pCOLADuet-1 carrying *mazF* under response promoter PprgQ	This study
pCOLA-PluxI I11-GFP(M)-PprgQ-*mazF*	pCOLADuet-1 carrying GFP(M),	This study

	mazF under response promoter PluxI I11 and PprgQ, respectively	
pACYC-P2-*luxI*(M)-P2-*luxR*(M)	pACYCDuet-1 carrying *luxI*(M) and *luxR*(M) under P2 promoter, respectively	This study
pACYC-P1-*prgX*(M)-P3-*ccfA*(M)-Ptrc-*prgZ*(M)	pACYCDuet-1 carrying *prgX*(M) with P1 promoter, *ccfA*(M) with P3 promoter, *prgZ*(M) with Ptrc promoter	This study
pACYC-P2-*luxI*(M)-P2-*luxR*(M)-P1-*prgX*-P3-*ccfA*-Ptrc-*prgZ*	pACYCDuet-1 carrying *luxI*(M) with P2 promoter, *luxR*(M) with P2 promoter, *prgX* with P1 promoter, *ccfA* with P3 promoter, *prgZ* with Ptrc promoter	This study
pACYC-P2-*luxI*-P2-*luxR*-P1-*prgX*(M)-P3-*ccfA*(M)-Ptrc-*prgZ*(M)	pACYCDuet-1 carrying *luxI* with P2 promoter, *luxR* with P2 promoter, *prgX*(M) with P1 promoter, *ccfA*(M) with P3 promoter, *prgZ*(M) with Ptrc promoter	This study
pACYC-P2-*luxI*(M)-P2-*luxR*(M)-P1-*prgX*(M)-P3-*ccfA*(M)-Ptrc-*prgZ*(M)	pACYCDuet-1 carrying *luxI*(M) with P2 promoter, *luxR*(M) with P2 promoter, *prgX*(M) with P1 promoter, *ccfA*(M) with P3 promoter, *prgZ*(M)	This study

	with Ptrc promoter	
pCOLA-PluxI 11-GFP(M)-PprqQ-*mazF*-PprgQ-β(M)	pCOLADuet-1 carrying GFP(M) with PluxI I11 promoter, *mazF* with PprgQ promoter, β(M) with PprgQ promoter	This study
pCOLA-PluxI 11-GFP(M)-PprqQ-*mazF*-PprgQ-β'(M)	pCOLADuet-1 carrying GFP(M) with PluxI I11 promoter, *mazF* with PprgQ promoter, β'(M) with PprgQ promoter	This study
pCOLA-PluxI 11-GFP(M)-PprqQ-mazF-PprgQ-α2(M)	pCOLADuet-1 carrying GFP(M) with PluxI I11 promoter, *mazF* with PprgQ promoter, α2(M) with PprgQ promoter	This study
pCOLA-PluxI I11-GFP(M)-PprqQ-*mazF*-PprgQ-β(M)-PprgQ-β'(M)	pCOLADuet-1 carrying GFP(M) with PluxI I11 promoter, *mazF* with PprgQ promoter, β(M) with PprgQ promoter, β'(M) with PprgQ promoter	This study
pCOLA-PluxI I11-GFP(M)-PprqQ-*mazF*-PprgQ-EF-Ts(M)	pCOLADuet-1 carrying GFP(M) with PluxI I11 promoter, *mazF* with PprgQ promoter, EF-Ts(M) with PprgQ promoter	This study
pCOLA-PluxII11-GFP(M)-PprgQ-*mazF*-PprgQ-S9(M)	pCOLADuet-1 carrying GFP(M) with PluxI I11 promoter, *mazF* with PprgQ promoter, EF-Ts(M) with PprgQ promoter	This study

pCOLA-PluxI 11-GFP(M)-PprqQ-*mazF*-PprgQ-S20(M)	pCOLADuet-1 carrying GFP(M) with PluxI I11 promoter, *mazF* with PprgQ promoter, S20(M) with PprgQ promoter	This study
pCOLA-PluxI I11-GFP(M)-PprqQ-*mazF*-PprgQ-L17(M)	pCOLADuet-1 carrying GFP(M) with PluxI I11 promoter, *mazF* with PprgQ promoter, L17(M) with PprgQ promoter	This study
pCOLA-PluxI I11-GFP(M)-PprgQ-*mazF*-PprgQ-EF-Ts(M)-PprgQ-S9(M)	pCOLADuet-1 carrying GFP(M) with PluxI I11 promoter, *mazF* with PprgQ promoter, EF-Ts(M) with PprgQ promoter, S9(M) with PprgQ promoter	This study
pCOLA-PluxI I11-GFP(M)-PprqQ-mazF-PprgQ-EF-Ts(M)-PprgQ-S20(M)	pCOLADuet-1 carrying GFP(M) with PluxI I11 promoter, *mazF* with PprgQ promoter, EF-Ts(M) with PprgQ promoter, S20(M) with PprgQ promoter	This study
pCOLA-PluxI I11-GFP(M)-PprqQ-*mazF*-PprgQ-EF-Ts(M)-PprgQ-L17(M)	pCOLADuet-1 carrying GFP(M) with PluxI I11 promoter, *mazF* with PprgQ promoter, EF-Ts(M) with PprgQ promoter, L17(M) with PprgQ promoter	This study

pCOLA-PluxI I11-GFP(M)-PprqQ-*mazF*-PprgQ-EF-Ts(M)-PprgQ-β(M)-PprgQ-β'(M)	pCOLADuet-1 carrying GFP(M) with PluxI I11 promoter, *mazF* with PprgQ promoter, EF-Ts(M) with PprgQ promoter, β(M) with PprgQ promoter, β'(M) with PprgQ promoter	This study
pCOLA-PluxI I11-*bktB*-PluxI I11-*fadB*-PluxI I11-*ter*- PluxI I11-*ydiI*	pCOLADuet-1 carrying *bktB*, *fadB*, *ter*, *ydiI* under PluxI I11 promoter, respectively	This study
pETD-PluxI I11-*bktB*-PluxI I11-*fadB*-PluxI I11-*ter*-PluxI I11-*ydiI*	pETDuet-1 carrying *bktB*, *fadB*, *ter*, *ydiI* under PluxI I11 promoter, respectively	This study
pETD-PluxI-*bktB*-PluxI-*fadB*-PluxI-*ter*-PluxI-*ydiI*	pETDuet-1 carrying *bktB*, *fadB*, *ter*, *ydiI* under PluxI promoter, respectively	This study
pETD-PluxI I11-*bktB*(M)-PluxI I11-*fadB*(M)-PluxI I11-*ter*(M)-PluxI I11-*ydiI*(M)	pETDuet-1 carrying *bktB*(M), *fadB*(M), *ter*(M), *ydiI*(M) under PluxI I11 promoter, respectively	This study
pCOLA-PprgQ-*mazF*	pCOLADuet-1 carrying *mazF* under PprgQ promoter	This study
pCOLA-PprqQ-*mazF*-PprgQ-β(M)	pCOLADuet-1 carrying *mazF*, β(M) under PprgQ promoter, respectively	This study
pCOLA-PprqQ-*mazF*-PprgQ-β'(M)	pCOLADuet-1 carrying *mazF*, β'(M)	This study

	under PprgQ promoter, respectively	
pCOLA-PprqQ-*mazF*-PprgQ-β(M)-PprgQ-β'(M)	pCOLADuet-1 carrying *mazF*, β(M), β'(M) under PprgQ promoter, respectively	This study
pCOLA-PprqQ-*mazF*-PprgQ-EF-Ts(M)	pCOLADuet-1 carrying *mazF*, EF-Ts(M) under PprgQ promoter, respectively	This study
pCOLA-PprqQ-*mazF*-PprgQ-S9(M)	pCOLADuet-1 carrying *mazF*, S9(M) under PprgQ promoter, respectively	This study
pCOLA-PprqQ-*mazF*-PprgQ-S20(M)	pCOLADuet-1 carrying *mazF*, S20(M) under PprgQ promoter, respectively	This study
pCOLA-PprqQ-*mazF*-PprgQ-L17(M)	pCOLADuet-1 carrying *mazF*, L17(M) under PprgQ promoter, respectively	This study
pCOLA- PprqQ-*mazF*-PprgQ-EF-Ts(M)-PprgQ-S9(M)	pCOLADuet-1 carrying *mazF*, EF-Ts(M), S9(M) under PprgQ promoter, respectively	This study
pCOLA-PprqQ-*mazF*-PprgQ-EF-Ts(M)-PprgQ-S20(M)	pCOLADuet-1 carrying *mazF*, EF-Ts(M), S20(M) under PprgQ promoter, respectively	This study
pCOLA-PprqQ-*mazF*-PprgQ-	pCOLADuet-1 carrying *mazF*,	This study

EF-Ts(M)-PprgQ-L17(M)	EF-Ts(M), L17(M) under PprgQ promoter, respectively	
pCOLA-PprqQ-*mazF*-PprgQ-EF-Ts(M)-PprgQ-β(M)-PprgQ-β'(M)	pCOLADuet-1 carrying *mazF*, EF-Ts(M), β(M), β'(M) under PprgQ promoter, respectively	This study

DNA sequences of modified genes

Gene sequence of native GFP (GFP(N)): Containing 27 MazF recognition sites

ATGGGTAAGGGAGAAGAACTTTTCACTGGAGTTGTCCCAATTCTTGTTGAATTAGATGGTGATGTTAATGGGCACAAATTTTCTGTCAGTGGAGAGGGTGAAGGTGATGCAACATACGGAAAACTTACCCTTAAATTTATTTGCACTACTGGAAAGCTTCCTGTTCCTTGGCCAACACTTGTCACTACTCTTACTTATGGTGTTCAATGCTTTTCAAGATACCCAGATCATATGAAGCGGCACGACTTCTTCAAGAGCGCCATGCCTGAGGGATACGTGCAGGAGAGGACCATCTTCTTCAAGGACGACGGGAACTACAAGACACGTGCTGAAGTCAAGTTTGAGGGAGACACCCTCGTCAACAGAATCGAGCTTAAGGGAATCGATTTCAAGGAGGACGGAAACATCCTCGGCCACAAGTTGGAATACAACTACAACTCCCACAACGTATACATCATGGCAGACAAACAAAAGAATGGAATCAAAGTTAACTTCAAAATTAGACACAACATTGAAGATGGAAGCGTTCAACTAGCAGACCATTATCAACAAAATACTCCAATTGGCGATGGCCCTGTCCTTTTACCAGACAACCATTACCTGTCCACACAATCTGCCCTTTCGAAAGATCCCAACGAAAAGAGAGACCACATGGTCCTTCTTGAGTTTGTAACAGCTGCTGGGATTACACATGGCATGGATGAACTATACAAATAA

Gene sequence of modified GFP (GFP(M)): Without MazF recognition sites

ATGGGTAAGGGAGAAGAACTTTTCACTGGAGTTGTCCCAATTCTTGTTGAATTAGATGGTGATGTTAATGGGCATAAATTTTCTGTCAGTGGAGAGGGTGAAGGTGATGCAACCTACGGAAAACTTACCCTTAAATTTATTTGCACTACTGGAAAGCTTCCTGTTCCTTGGCCAACCCTTGTCACTACTCTTACTTATGGTGT

TCAATGCTTTTCAAGATACCCAGATCATATGAAGCGGCACGACTTCTTCAAGAGCGCCATGCCTGAGGGATACGTGCAGGAGAGGACCATCTTCTTCAAGGACGACGGGAACTATAAGACCCGTGCTGAAGTCAAGTTTGAGGGAGATACCCTCGTCAAAAGAATCGAGCTTAAGGGAATCGATTTCAAGGAGGACGGAAATATCCTCGGCCATAAGTTGGAATATAACTATAACTCCCATAACGTATATATCATGGCAGATAAGCAAAAGAATGGAATCAAAGTTAACTTCAAAATTAGGCATAATATTGAAGATGGAAGCGTTCAACTAGCAGACCATTATCAGCAAAATACTCCAATTGGCGATGGCCCTGTCCTTTTACCAGATAACCATTACCTGTCCACCCAATCTGCCCTTTCGAAAGATCCCAACGAAAAGAGAGACCATATGGTCCTTCTTGAGTTTGTAACCGCTGCTGGGATTACGCATGGCATGGATGAACTATATAAATAA

Gene sequence of native LuxI (LuxI(N)): Containing 7 MazF recognition sites

ATGATAAAAAAATCGGACTTTTTGGGCATTCCATCAGAGGAGTATAGAGGTATTCTTAGTCTTCGTTATCAGGTATTTAAACGAAGACTGGAGTGGGACTTGGTAAGTGAGGATAATCTTGAATCAGATGAATATGATAACTCAAATGCAGAATATATTTATGCTTGTGATGATGCGGAAGAGGTAAATGGCTGTTGGCGTTTGTTACCTACAACGGGTGATTACATGTTAAAAACTGTTTTTCCTGAATTGCTCGGAGATCAAGTAGCCCCAAGAGATCCAAATATAGTCGAATTAAGCCGTTTTGCTGTGGGAAAAAATAGCTCAAAAATAAATAACTCTGCTAGTGAAATAACAATGAAATTGTTTCAAGCTATATATAAACACGCAGTTAGTCAAGGTATTACAGAATATGTAACAGTAACATCAATAGCAATAGAGCGATTTCTGAAA

CGTATTAAAGTTCCTTGTCATCGCATTGGTGATAAGGAGATTCATTTATTAGGTAATACTAGATCTGTTGTATTGTCTATGCCTATTAATGATCAGTTTAGAAAAGCTGTATCAAATTAA

Gene sequence of modified LuxI (LuxI(M)): Without MazF recognition sites

ATGATAAAAAAATCGGACTTTTTGGGCATTCCATCAGAGGAGTATAGAGGTATTCTTAGTCTTCGTTATCAGGTATTTAAACGAAGACTGGAGTGGGACTTGGTAAGTGAGGATAATCTTGAATCAGATGAATATGATAACTCAAATGCAGAATATATTTATGCTTGTGATGATGCGGAAGAGGTAAATGGCTGTTGGCGTTTGTTACCTACCACGGGTGATTATATGTTAAAAACTGTTTTCCTGAATTGCTCGGAGATCAAGTAGCCCCAAGAGATCCAAATATAGTCGAATTAAGCCGTTTTGCTGTGGGAAAAAATAGCTCAAAAATAAATAACTCTGCTAGTGAAATAACCATGAAATTGTTTCAAGCTATATATAAGCACGCAGTTAGTCAAGGTATTACCGAATATGTAACCGTAACCTCAATAGCAATAGAGCGATTTCTGAAACGTATTAAAGTTCCTTGTCATCGCATTGGTGATAAGGAGATTCATTTATTAGGTAATACTAGATCTGTTGTATTGTCTATGCCTATTAATGATCAGTTTAGAAAAGCTGTATCAAATTAA

Gene sequence of native LuxR (LuxR(N)): Containing 11 MazF recognition sites

ATGAACATTAAAAATATAAATGCTAATGAGAAGATAATTGATAAAATTAAAACTTGTAATAATAATAAAGATATTAATCAATGTTTATCTGAAATAGCAAAGATAATACATTGTGAATATTACCTATTCGCTATTATCTATCCTCACTCAATAATTAAACCTGATGTTTCAATTATAGATAATTACCCTGAAAAATGGCGTA

AATATTATGATGATGCCGGACTACTAGAATATGACCCTGTAGTCGATTACT
CTAAGTCCCATCATTCACCAATTAATTGGAACGTATTCGAAAAAAAAACA
ATAAAAAAAGAGTCTCCGAATGTAATAAAAGAAGCACAGGAATCGGGAC
TCATTACTGGATTTAGCTTTCCAATTCATACTGCAAGTAATGGTTTTGGAA
TGCTCAGTTTTGCTCATTCAGATAAAGATATTTATACTGACAGTTTATTTTT
ACACGCTAGTACAAATGTACCATTAATGCTTCCTTCTTTAGTCGATAATTA
TCAAAAAATAAATACGACACGTAAAAAGTCAGATTCTATTTTAACAAAAA
GAGAAAAAGAATGCTTAGCGTGGGCGAGTGAAGGAAAAAGTACATGGGA
TATTTCAAAAATACTTGGCTGCAGTGAGCGTACTGTCACTTTTCATTTAAC
CAATACTCAAATGAAACTCAATACAACTAACCGCTGCCAAAGTATTTCTA
AAGCAATTTTAACTGGCGCCATTAATTGTCCATACCTTAAAAATTAA

Gene sequence of modified LuxR (LuxR(M)): Without MazF recognition sites

ATGAATATTAAAAATATAAATGCTAATGAGAAGATAATTGATAAAATTAA
AACTTGTAATAATAATAAAGATATTAATCAATGTTTATCTGAAATAGCAA
AGATAATTCATTGTGAATATTACCTATTCGCTATTATCTATCCTCACTCAAT
AATTAAACCTGATGTTTCAATTATAGATAATTACCCTGAAAAATGGCGTA
AATATTATGATGATGCCGGACTACTAGAATATGACCCTGTAGTCGATTACT
CTAAGTCCCATCATTCACCAATTAATTGGAACGTATTCGAAAAAAAAACC
ATAAAAAAAGAGTCTCCGAATGTAATAAAAGAAGCGCAGGAATCGGGAC
TCATTACTGGATTTAGCTTTCCAATTCATACTGCAAGTAATGGTTTTGGAA
TGCTCAGTTTTGCTCATTCAGATAAAGATATTTATACTGATAGTTTATTTTT
GCACGCTAGTACCAATGTACCATTAATGCTTCCTTCTTTAGTCGATAATTA

TCAAAAAATAAATACGACCCGTAAAAAGTCAGATTCTATTTTAACCAAAA
GAGAAAAAGAATGCTTAGCGTGGGCGAGTGAAGGAAAAAGTACCTGGGA
TATTTCAAAAATACTTGGCTGCAGTGAGCGTACTGTCACTTTTCATTTAAC
CAATACTCAAATGAAACTCAATACCACTAACCGCTGCCAAAGTATTTCTA
AAGCAATTTTAACTGGCGCCATTAATTGTCCATACCTTAAAAATTAA

Gene sequence of native CcfA (CcfA(N)): Containing 13 MazF recognition sites

ATGGTTGTGAAGAAGTATAAGCGCTTATTATTAATGGCTGGGTTAGTGAC
CTTAGTCTTTGTTTTATCCGCCTGCGGAACCGCGCCCGTAAGTGAAAGTAG
TACAGGAATTTGGGATCGATACATTGTTTATTATTTTGCCCAAGCGATTAA
GTTCCTATCATTAGGCGGCAGTGTCGGAATTGGGATTATCCTATTTACGTT
AGTCATCCGGATTATTTTATTGCCGTTGATGCATTTCCAAACGAAGAGTAT
GCGTAAAACACAAGAGTTGCAACCGCAATTAAAGGCCTTGCAACAAAAAT
ATTCTTCAAAAGATCCTGAAACACAACGCTTATTCCGAGAAGAACAGCAA
CGTTTGTATGCAGAGAATAATGTGAATCCTTACATTGGCTGTTTACCACTG
TTGGTTCAACTACCAATCATGATGGCTTTGTATCAAGCCATTTCACGCGTG
CCTGAGTTAAAAGAAGGAACGTTCTTGTGGTTAAGCCTCGATAAGCCGGA
TCCGTATTTAATTTTACCGATTTTAGCGGCTGTCTTTACGTTTGCTAGTACG
TATCTTTCAAGTATGAGCCAACTAGAAACAAATGCCTCACTGAAAATTAT
GAATTACGTTATGCCAGCGATGATCTTCTTTATGGGGATTTCATTAGCCAG
CAGTTTATCTCTTTACTGGGTGGTCTCAAATGCCTTCCAAACAGGGCAAAC
ATTGTTATTAAACAACCCATTCAAAATCCGTAAAGAGCGGGAGGAAGCAG

CACGTCAAGCCAAAGCACGGGAGCGAGCGCTTGAACGAGCAAAAAGTCC
TAAGAAAAAAGGGAAGAAAAAATAA

Gene sequence of modified CcfA (CcfA(M)): Without MazF recognition sites

ATGGTTGTGAAGAAGTATAAGCGCTTATTATTAATGGCTGGGTTAGTGAC
CTTAGTCTTTGTTTTATCCGCCTGCGGAACCGCGCCCGTAAGTGAAAGTAG
TACCGGAATTTGGGATCGATATATTGTTTATTATTTTGCCCAAGCGATTAA
GTTCCTATCATTAGGCGGCAGTGTCGGAATTGGGATTATCCTATTTACGTT
AGTCATCCGGATTATTTTATTGCCGTTGATGCATTTCCAAACGAAGAGTAT
GCGTAAAACCCAAGAGTTGCAACCGCAATTAAAGGCCTTGCAGCAAAAAT
ATTCTTCAAAAGATCCTGAAACCCAACGCTTATTCCGAGAAGAGCAGCAA
CGTTTGTATGCAGAGAATAATGTGAATCCTTATATTGGCTGTTTACCACTG
TTGGTTCAACTACCAATCATGATGGCTTTGTATCAAGCCATTTCACGCGTG
CCTGAGTTAAAAGAAGGAACGTTCTTGTGGTTAAGCCTCGATAAGCCGGA
TCCGTATTTAATTTTACCGATTTTAGCGGCTGTCTTTACGTTTGCTAGTACG
TATCTTTCAAGTATGAGCCAACTAGAAACCAATGCCTCACTGAAAATTAT
GAATTACGTTATGCCAGCGATGATCTTCTTTATGGGGATTTCATTAGCCAG
CAGTTTATCTCTTTACTGGGTGGTCTCAAATGCCTTCCAAACCGGGCAAAC
CTTGTTATTAAATAACCCATTCAAAATCCGTAAAGAGCGGGAGGAAGCAG
CACGTCAAGCCAAAGCACGGGAGCGAGCGCTTGAACGAGCAAAAAGTCC
TAAGAAAAAAGGGAAGAAAAAATAA

Gene sequence of native PrgX (PrgX(N)): Containing 20 MazF recognition sites

ATGTTTAAGATAGGTTCTGTCCTGAAACAAATAAGGCAAGAGTTAAATTA
CCATCAGATTGATTTATATTCTGGAATAATGTCCAAATCTGTTTACATAAA
AGTTGAAGCTGACTCTCGACCGATTTCTGTAGAAGAGTTAAGCAAGTTCTC
AGAACGTTTAGGTGTAAATTTTTTTGAAATTTTAAATAGAGCTGGCATGAA
TACAAAAAGCGTAAATGAAACAGGAAAAGAAAAACTTCTTATTTCAAAA
ATATTTACAAACCCTGACTTATTTGACAAAAATTTTCAAAGGATTGAGCCA
AAGCGCTTAACTAGTCTTCAGTACTTTAGTATTTATCTTGGATATATTTCTA
TTGCACATCATTATAACATTGAAGTTCCTACTTTTAATAAAACAATTACTT
CAGATTTAAAACATTTATATGATAAAAGAACAACATTTTTTGGAATTGATT
ATGAAATTGTTTCAAATCTTTTAAATGTCTTACCGTATGAAGAAGTTTCAA
GTATCATTAAGCCTATGTATCCAATCGTGGATTCCTTTGGTAAAGATTACG
ATTTAACTATTCAGACTGTTCTAAAAAATGCTTTAACAATATCAATTATGA
ATAGAAATCTAAAAGAAGCGCAGTATTATATTAATCAATTTGAACACTTA
AAAACAATTAAAAATATTAGCATAAATGGATATTACGACTTAGAAATAAA
CTATTTAAAACAAATATATCAATTTTTAACTGACAAAAATATTGATTCTTA
TTTAAATGCGGTAAATATTATCAACATCTTTAAAATCATTGGAAAAGAAG
ATATTCACAGATCTTTAGTTGAGGAACTTACAAAAATATCTGCAAAAGAA
AAATTTACGCCCCCTAAAGAAGTAACCATGTATTATGAAAATTACGTTGC
AATAGAAAATAATCCTATTCCTGAAATAAAAGAGCAGTCATGA

Gene sequence of modified PrgX (PrgX(M)): Without MazF recognition sites

ATGTTTAAGATAGGTTCTGTCCTGAAGCAAATAAGGCAAGAGTTAAATTA
CCATCAGATTGATTTATATTCTGGAATAATGTCCAAATCTGTTTATATAAA

AGTTGAAGCTGACTCTCGACCGATTTCTGTAGAAGAGTTAAGCAAGTTCTCAGAACGTTTAGGTGTAAATTTTTTTGAAATTTTAAATAGAGCTGGCATGAATACCAAAAGCGTAAATGAAACCGGAAAAGAAAAACTTCTTATTTCAAAAATATTTACCAACCCTGACTTATTTGATAAAAATTTTCAAAGGATTGAGCCAAAGCGCTTAACTAGTCTTCAGTACTTTAGTATTTATCTTGGATATATTTCTATTGCGCATCATTATAATATTGAAGTTCCTACTTTTAATAAAACCATTACTTCAGATTTAAACCATTTATATGATAAAAGAACCACCTTTTTTGGAATTGATTATGAAATTGTTTCAAATCTTTTAAATGTCTTACCGTATGAAGAAGTTTCAAGTATCATTAAGCCTATGTATCCAATCGTGGATTCCTTTGGTAAAGATTACGATTTAACTATTCAGACTGTTCTAAAAAATGCTTTAACCATATCAATTATGAATAGAAATCTAAAAGAAGCGCAGTATTATATTAATCAATTTGAGCACTTAAAAACCATTAAAAATATTAGCATAAATGGATATTACGACTTAGAAATAAACTATTTAAAGCAAATATATCAATTTTTAACTGATAAAAATATTGATTCTTATTTAAATGCGGTAAATATTATCAATATCTTTAAAATCATTGGAAAAGAAGATATTCATAGATCTTTAGTTGAGGAACTTACCAAAATATCTGCAAAAGAAAAATTTACGCCCCCTAAAGAAGTAACCATGTATTATGAAAATTACGTTGCAATAGAAAATAATCCTATTCCTGAAATAAAAGAGCAGTCATGA

Gene sequence of native PrgZ (PrgZ(N)): Containing 31 MazF recognition sites

ATGAAGAAGTACAAGAAGTTTTGTTTTTTAGGTATTGGGTTATTACCTTTGGTATTAGCTAGTTGTGGGACAAATACTGCCACAAAAGATTCACAAGATGCAACAGAAAAAAAAGTAGAACAGGTAGCTACTTTGACTGCAGGGACACCT

810 GTCCAAAGTTTAGACCCAGCAACTGCTGTAGATCAAACGAGTATAACTTT

811 ATTAGCCAATGTGATGGAGGGGTTGTATCGATTAGATGAAAAAAATCAAC

812 CGCAACCAGCCATTGCAGCTGGTCAACCAAAAGTATCGAATAATGGCAAA

813 ACTTATACCATTGTGATTAGAGATGGCGCTAAGTGGTCTGATGGTACACA

814 AATAACTGCTAGTGATTTTGTGGCCGCGTGGCAAAGAGTTGTAGATCCTA

815 AAACAGCTTCTCCAAATGTGGAACTGTTTTCTGCTATAAAAAATGCCAAA

816 GAAATTGCTTCAGGAAAACAAGCAAAAGATACTTTAGCAGTGAAAAGTAT

817 TGGTGAGAAAACATTAGAAATTGAATTAGTTGAACCAACACCTTATTTTA

818 CTGATCTGTTATCCTTAACCGCTTACTATCCAGTACAGCAGAAAGCAATTA

819 AAGAGTATGGGAAAGACTATGGGGTTTCTCAAAAAGCAATTGTAACAAAT

820 GGAGCATTTAACTTAACAAACTTAGAGGGAGTAGGAACTTCTGATAAGTG

821 GACGATTTCTAAAAATAAAGAGTACTGGGATCAAAAAGATGTTTCTATGG

822 ATAAAATTAATTTTCAAGTCGTCAAAGAAATTAATACAGGAATAAATTTG

823 TATAATGATGGGCAATTAGATGAGGCGCCTTTAGCTGGTGAATATGCAAA

824 ACAATACAAGAAAGATAAAGAATATTCAACAACATTAATGGCTAATACAA

825 TGTTTTTAGAAATGAACCAAACTGGGGAAAATAAGCTTTTACAAAACAAA

826 AATGTCCGAAAAGCAATTAGCTATGCAATTGATCGGGAAAGTCTAGTTAA

827 AAAATTACTAGATAATGGTTCCGTTGCTTCTGTTGGCGTAGTACCAAAAGA

828 AATGGCCTTTAATCCGGTAAATAAAAAAGATTTTGCTAATGAAAAATTAG

829 TTGAATTTAACAAAAAACAAGCAGAAGAGTATTGGGATAAGGCTAAAAA

830 AGAAATCGATTTATCCAAAAATACTTCTTTAGATTTACTTGTAAGTGATGG

831 AGAGTTTGAAAAAAAGGCAGGAGAATTTCTGCAAGGACAGTTGCAAGAT

AGCTTAGAAGGATTGAAGGTTAATGTGACGCCAATTCCTGCAAATGTCTTT
ATGGAACGCCTAACAAAAAAGGATTTTACTTTGAGCCTAAGTGGATGGCA
AGCTGATTATGCAGACCCTATTAGCTTTTTAGCAAACTTTGAAACAAATAG
TCCAATGAATCATGGTGGATATTCAAATAAAAATTATGATGAATTGCTAA
AGGATTCTTCTTCTAAACGTTGGCAAGAATTGAAAAAAGCTGAAAAATTG
TTGATCAATGATATGGGGGTCGTTCCAATTTTTCAAGTTGGAACAGCAAA
ATTAGAAAAAAGTAAAATAAAAAATGTTTTAATGCATTCAATAGGAGCAA
AATATGACTACAAAAAAATGAGAATAGAGAAGTAA

Gene sequence of modified PrgZ (PrgZ(M)): Without MazF recognition sites

ATGAAGAAGTATAAGAAGTTTTGTTTTTTAGGTATTGGGTTATTACCTTTG
GTATTAGCTAGTTGTGGGACCAATACTGCCACCAAAGATTCGCAAGATGC
AACCGAAAAAAAAGTAGAGCAGGTAGCTACTTTGACTGCAGGGACCCCTG
TCCAAAGTTTAGACCCAGCAACTGCTGTAGATCAAACGAGTATAACTTTA
TTAGCCAATGTGATGGAGGGGTTGTATCGATTAGATGAAAAAAATCAACC
GCAACCAGCCATTGCAGCTGGTCAACCAAAAGTATCGAATAATGGCAAAA
CTTATACCATTGTGATTAGAGATGGCGCTAAGTGGTCTGATGGTACCCAA
ATAACTGCTAGTGATTTTGTGGCCGCGTGGCAAAGAGTTGTAGATCCTAA
AACCGCTTCTCCAAATGTGGAACTGTTTTCTGCTATAAAAAATGCCAAAG
AAATTGCTTCAGGAAAGCAAGCAAAAGATACTTTAGCAGTGAAAAGTATT
GGTGAGAAAACCTTAGAAATTGAATTAGTTGAACCAACCCCTTATTTTACT
GATCTGTTATCCTTAACCGCTTACTATCCAGTGCAGCAGAAAGCAATTAAA
GAGTATGGGAAAGACTATGGGGTTTCTCAAAAAGCAATTGTAACCAATGG

AGCATTTAACTTAACCAACTTAGAGGGAGTAGGAACTTCTGATAAGTGGA
CGATTTCTAAAAATAAAGAGTACTGGGATCAAAAAGATGTTTCTATGGAT
AAAATTAATTTTCAAGTCGTCAAAGAAATTAATACCGGAATAAATTTGTA
TAATGATGGGCAATTAGATGAGGCGCCTTTAGCTGGTGAATATGCAAAGC
AATATAAGAAAGATAAAGAATATTCAACCACCTTAATGGCTAATACCATG
TTTTTAGAAATGAACCAAACTGGGGAAAATAAGCTTTTGCAAAATAAAAA
TGTCCGAAAAGCAATTAGCTATGCAATTGATCGGGAAAGTCTAGTTAAAA
AATTACTAGATAATGGTTCCGTTGCTTCTGTTGGCGTAGTACCAAAAGAAA
TGGCCTTTAATCCGGTAAATAAAAAAGATTTTGCTAATGAAAAATTAGTT
GAATTTAATAAAAAGCAAGCAGAAGAGTATTGGGATAAGGCTAAAAAAG
AAATCGATTTATCCAAAAATACTTCTTTAGATTTACTTGTAAGTGATGGAG
AGTTTGAAAAAAAGGCAGGAGAATTTCTGCAAGGGCAGTTGCAAGATAG
CTTAGAAGGATTGAAGGTTAATGTGACGCCAATTCCTGCAAATGTCTTTAT
GGAACGCCTAACCAAAAAGGATTTTACTTTGAGCCTAAGTGGATGGCAAG
CTGATTATGCAGACCCTATTAGCTTTTTAGCAAACTTTGAAACCAATAGTC
CAATGAATCATGGTGGATATTCAAATAAAAATTATGATGAATTGCTAAAG
GATTCTTCTTCTAAACGTTGGCAAGAATTGAAAAAAGCTGAAAAATTGTT
GATCAATGATATGGGGGTCGTTCCAATTTTTCAAGTTGGAACCGCAAAATT
AGAAAAAAGTAAAATAAAAAATGTTTTAATGCATTCAATAGGAGCAAAAT
ATGACTATAAAAAAATGAGAATAGAGAAGTAA

Gene sequence of native MazF (MazF(N)): Containing 9 MazF recognition sites

ATGGTAAGCCGATACGTACCCGATATGGGCGATCTGATTTGGGTTGATTTT
GACCCGACAAAAGGTAGCGAGCAAGCTGGACATCGTCCAGCTGTTGTCCT
GAGTCCTTTCATGTACAACAACAAAACAGGTATGTGTCTGTGTGTTCCTTG
TACAACGCAATCAAAAGGATATCCGTTCGAAGTTGTTTTATCCGGTCAGG
AACGTGATGGCGTAGCGTTAGCTGATCAGGTAAAAAGTATCGCCTGGCGG
GCAAGAGGAGCAACGAAGAAAGGAACAGTTGCCCCAGAGGAATTACAAC
TCATTAAAGCCAAAATTAACGTACTGATTGGGTAG

Gene sequence of modified MazF (MazF(M)): Without MazF recognition sites

ATGGTAAGCCGATACGTACCCGATATGGGCGATCTGATTTGGGTTGATTTT
GACCCGACCAAAGGTAGCGAGCAAGCTGGCCATCGTCCAGCTGTTGTCCT
GAGTCCTTTCATGTATAATAATAAAACCGGTATGTGTCTGTGTGTTCCTTG
TACCACGCAATCAAAAGGATATCCGTTCGAAGTTGTTTTATCCGGTCAGG
AACGTGATGGCGTAGCGTTAGCTGATCAGGTAAAAAGTATCGCCTGGCGG
GCAAGAGGAGCAACGAAGAAAGGAACCGTTGCCCCAGAGGAATTGCAAC
TCATTAAAGCCAAAATTAACGTACTGATTGGGTAG

Gene sequence of native α_2 (α_2(N)): Containing 10 MazF recognition sites

ATGCAGGGTTCTGTGACAGAGTTTCTAAAACCGCGCCTGGTTGATATCGA
GCAAGTGAGTTCGACGCACGCCAAGGTGACCCTTGAGCCTTTAGAGCGTG
GCTTTGGCCATACTCTGGGTAACGCACTGCGCCGTATTCTGCTCTCATCGA
TGCCGGGTTGCGCGGTGACCGAGGTTGAGATTGATGGTGTACTACATGAG
TACAGCACCAAAGAAGGCGTTCAGGAAGATATCCTGGAAATCCTGCTCAA

CCTGAAAGGGCTGGCGGTGAGAGTTCAGGGCAAAGATGAAGTTATTCTTA
CCTTGAATAAATCTGGCATTGGCCCTGTGACTGCAGCCGATATCACCCACG
ACGGTGATGTCGAAATCGTCAAGCCGCAGCACGTGATCTGCCACCTGACC
GATGAGAACGCGTCTATTAGCATGCGTATCAAAGTTCAGCGCGGTCGTGG
TTATGTGCCGGCTTCTACCCGAATTCATTCGGAAGAAGATGAGCGCCCAA
TCGGCCGTCTGCTGGTCGACGCATGCTACAGCCCTGTGGAGCGTATTGCCT
ACAATGTTGAAGCAGCGCGTGTAGAACAGCGTACCGACCTGGACAAGCTG
GTCATCGAAATGGAAACCAACGGCACAATCGATCCTGAAGAGGCGATTCG
TCGTGCGGCAACCATTCTGGCTGAACAACTGGAAGCTTTCGTTGACTTACG
TGATGTACGTCAGCCTGAAGTGAAAGAAGAGAAACCAGAGTTCGATCCGA
TCCTGCTGCGCCCTGTTGACGATCTGGAATTGACTGTCCGCTCTGCTAACT
GCCTTAAAGCAGAAGCTATCCACTATATCGGTGATCTGGTACAGCGTACC
GAGGTTGAGCTCCTTAAAACGCCTAACCTTGGTAAAAAATCTCTTACTGA
GATTAAAGACGTGCTGGCTTCCCGTGGACTGTCTCTGGGCATGCGCCTGG
AAAACTGGCCACCGGCAAGCATCGCTGACGAGTAA

Gene sequence of modified α_2 (α_2(M)): Without MazF recognition sites

ATGCAGGGTTCTGTGACCGAGTTTCTAAAACCGCGCCTGGTTGATATCGA
GCAAGTGAGTTCGACGCACGCCAAGGTGACCCTTGAGCCTTTAGAGCGTG
GCTTTGGCCATACTCTGGGTAACGCACTGCGCCGTATTCTGCTCTCATCGA
TGCCGGGTTGCGCGGTGACCGAGGTTGAGATTGATGGTGTACTGCATGAG
TATAGCACCAAAGAAGGCGTTCAGGAAGATATCCTGGAAATCCTGCTCAA
CCTGAAAGGGCTGGCGGTGAGAGTTCAGGGCAAAGATGAAGTTATTCTTA

CCTTGAATAAATCTGGCATTGGCCCTGTGACTGCAGCCGATATCACCCACG
ACGGTGATGTCGAAATCGTCAAGCCGCAGCACGTGATCTGCCACCTGACC
GATGAGAACGCGTCTATTAGCATGCGTATCAAAGTTCAGCGCGGTCGTGG
TTATGTGCCGGCTTCTACCCGAATTCATTCGGAAGAAGATGAGCGCCCAA
TCGGCCGTCTGCTGGTCGACGCATGCTATAGCCCTGTGGAGCGTATTGCCT
ATAATGTTGAAGCAGCGCGTGTAGAGCAGCGTACCGACCTGGATAAGCTG
GTCATCGAAATGGAAACCAACGGCACCATCGATCCTGAAGAGGCGATTCG
TCGTGCGGCAACCATTCTGGCTGAGCAACTGGAAGCTTTCGTTGACTTACG
TGATGTACGTCAGCCTGAAGTGAAAGAAGAGAAACCAGAGTTCGATCCGA
TCCTGCTGCGCCCTGTTGACGATCTGGAATTGACTGTCCGCTCTGCTAACT
GCCTTAAAGCAGAAGCTATCCACTATATCGGTGATCTGGTGCAGCGTACC
GAGGTTGAGCTCCTTAAAACGCCTAACCTTGGTAAAAAATCTCTTACTGA
GATTAAAGACGTGCTGGCTTCCCGTGGACTGTCTCTGGGCATGCGCCTGG
AAAACTGGCCACCGGCAAGCATCGCTGACGAGTAA

Gene sequence of native β (β(N)): Containing 58 MazF recognition sites

ATGGTTTACTCCTATACCGAGAAAAAACGTATTCGTAAGGATTTTGGTAA
ACGTCCACAAGTTCTGGATGTACCTTATCTCCTTTCTATCCAGCTTGACTC
GTTTCAGAAATTTATCGAGCAAGATCCTGAAGGGCAGTATGGTCTGGAAG
CTGCTTTCCGTTCCGTATTCCCGATTCAGAGCTACAGCGGTAATTCCGAGC
TGCAATACGTCAGCTACCGCCTTGGCGAACCGGTGTTTGACGTCCAGGAA
TGTCAAATCCGTGGCGTGACCTATTCCGCACCGCTGCGCGTTAAACTGCGT

942 CTGGTGATCTATGAGCGCGAAGCGCCGGAAGGCACCGTAAAAGACATTAA

943 AGAACAAGAAGTCTACATGGGCGAAATTCCGCTCATGACAGACAACGGTA

944 CCTTTGTTATCAACGGTACTGAGCGTGTTATCGTTTCCCAGCTGCACCGTA

945 GTCCGGGCGTCTTCTTTGACTCCGACAAAGGTAAAACCCACTCTTCGGGTA

946 AAGTGCTGTATAACGCGCGTATCATCCCTTACCGTGGTTCCTGGCTGGACT

947 TCGAATTCGATCCGAAGGACAACCTGTTCGTACGTATCGACCGTCGCCGT

948 AAACTGCCTGCGACCATCATTCTGCGCGCCCTGAACTACACCACAGAGCA

949 GATCCTCGACCTGTTCTTTGAAAAAGTTATCTTTGAAATCCGTGATAACAA

950 GCTGCAGATGGAACTGGTGCCGGAACGCCTGCGTGGTGAAACCGCATCTT

951 TTGACATCGAAGCTAACGGTAAAGTGTACGTAGAAAAAGGCCGCCGTATC

952 ACTGCGCGCCACATTCGCCAGCTGGAAAAAGACGACGTCAAACTGATCGA

953 AGTCCCGGTTGAGTACATCGCAGGTAAAGTGGTTGCTAAAGACTATATTG

954 ATGAGTCTACCGGCGAGCTGATCTGCGCAGCGAACATGGAGCTGAGCCTG

955 GATCTGCTGGCTAAGCTGAGCCAGTCTGGTCACAAGCGTATCGAAACGCT

956 GTTCACCAACGATCTGGATCACGGCCCATATATCTCTGAAACCTTACGTGT

957 CGACCCAACTAACGACCGTCTGAGCGCACTGGTAGAAATCTACCGCATGA

958 TGCGCCCTGGCGAGCCGCCGACTCGTGAAGCAGCTGAAAGCCTGTTCGAG

959 AACCTGTTCTTCTCCGAAGACCGTTATGACTTGTCTGCGGTTGGTCGTATG

960 AAGTTCAACCGTTCTCTGCTGCGCGAAGAAATCGAAGGTTCCGGTATCCT

961 GAGCAAAGACGACATCATTGATGTTATGAAAAAGCTCATCGATATCCGTA

962 ACGGTAAAGGCGAAGTCGATGATATCGACCACCTCGGCAACCGTCGTATC

963 CGTTCCGTTGGCGAAATGGCGGAAAACCAGTTCCGCGTTGGCCTGGTACG

964 TGTAGAGCGTGCGGTGAAAGAGCGTCTGTCTCTGGGCGATCTGGATACCC

965 TGATGCCACAGGATATGATCAACGCCAAGCCGATTTCCGCAGCAGTGAAA

966 GAGTTCTTCGGTTCCAGCCAGCTGTCTCAGTTTATGGACCAGAACAACCCG

967 CTGTCTGAGATTACGCACAAACGTCGTATCTCCGCACTCGGCCCAGGCGG

968 TCTGACCCGTGAACGTGCAGGCTTCGAAGTTCGAGACGTACACCCGACTC

969 ACTACGGTCGCGTATGTCCAATCGAAACCCCTGAAGGTCCGAACATCGGT

970 CTGATCAACTCTCTGTCCGTGTACGCACAGACTAACGAATACGGCTTCCTT

971 GAGACTCCGTATCGTAAAGTGACCGACGGTGTTGTAACTGACGAAATTCA

972 CTACCTGTCTGCTATCGAAGAAGGCAACTACGTTATCGCCCAGGCGAACT

973 CCAACTTGGATGAAGAAGGCCACTTCGTAGAAGACCTGGTAACTTGCCGT

974 AGCAAAGGCGAATCCAGCTTGTTCAGCCGCGACCAGGTTGACTACATGGA

975 CGTATCCACCCAGCAGGTGGTATCCGTCGGTGCGTCCCTGATCCCGTTCCT

976 GGAACACGATGACGCCAACCGTGCATTGATGGGTGCGAACATGCAACGTC

977 AGGCCGTTCCGACTCTGCGCGCTGATAAGCCGCTGGTTGGTACTGGTATG

978 GAACGTGCTGTTGCCGTTGACTCCGGTGTAACTGCGGTAGCTAAACGTGG

979 TGGTGTCGTTCAGTACGTGGATGCTTCCCGTATCGTTATCAAAGTTAACGA

980 AGACGAGATGTATCCGGGTGAAGCAGGTATCGACATCTACAACCTGACCA

981 AATACACCCGTTCTAACCAGAACACCTGTATCAACCAGATGCCGTGTGTG

982 TCTCTGGGTGAACCGGTTGAACGTGGCGACGTGCTGGCAGACGGTCCGTC

983 CACCGACCTCGGTGAACTGGCGCTTGGTCAGAACATGCGCGTAGCGTTCA

984 TGCCGTGGAATGGTTACAACTTCGAAGACTCCATCCTCGTATCCGAGCGTG

985 TTGTTCAGGAAGACCGTTTCACCACCATCCACATTCAGGAACTGGCGTGTG

986 TGTCCCGTGACACCAAGCTGGGTCCGGAAGAGATCACCGCTGACATCCCG

987 AACGTGGGTGAAGCTGCGCTCTCCAAACTGGATGAATCCGGTATCGTTTA

988 CATTGGTGCGGAAGTGACCGGTGGCGACATTCTGGTTGGTAAGGTAACGC

989 CGAAAGGTGAAACTCAGCTGACCCCAGAAGAAAAACTGCTGCGTGCGATC

990 TTCGGTGAGAAAGCCTCTGACGTTAAAGACTCTTCTCTGCGCGTACCAAAC

991 GGTGTATCCGGTACGGTTATCGACGTTCAGGTCTTTACTCGCGATGGCGTA

992 GAAAAAGACAAACGTGCGCTGGAAATCGAAGAAATGCAGCTCAAACAGG

993 CGAAGAAAGACCTGTCTGAAGAACTGCAGATCCTCGAAGCGGGTCTGTTC

994 AGCCGTATCCGTGCTGTGCTGGTAGCCGGTGGCGTTGAAGCTGAGAAGCT

995 CGACAAACTGCCGCGCGATCGCTGGCTGGAGCTGGGCCTGACAGACGAAG

996 AGAAACAAAATCAGCTGGAACAGCTGGCTGAGCAGTATGACGAACTGAA

997 ACACGAGTTCGAGAAGAAACTCGAAGCGAAACGCCGCAAAATCACCCAG

998 GGCGACGATCTGGCACCGGGCGTGCTGAAGATTGTTAAGGTATATCTGGC

999 GGTTAAACGCCGTATCCAGCCTGGTGACAAGATGGCAGGTCGTCACGGTA

1000 ACAAGGGTGTAATTTCTAAGATCAACCCGATCGAAGATATGCCTTACGAT

1001 GAAAACGGTACGCCGGTAGACATCGTACTGAACCCGCTGGGCGTACCGTC

1002 TCGTATGAACATCGGTCAGATCCTCGAAACCCACCTGGGTATGGCTGCGA

1003 AAGGTATCGGCGACAAGATCAACGCCATGCTGAAACAGCAGCAAGAAGT

1004 CGCGAAACTGCGCGAATTCATCCAGCGTGCGTACGATCTGGGCGCTGACG

1005 TTCGTCAGAAAGTTGACCTGAGTACCTTCAGCGATGAAGAAGTTATGCGT

1006 CTGGCTGAAAACCTGCGCAAAGGTATGCCAATCGCAACGCCGGTGTTCGA

1007 CGGTGCGAAAGAAGCAGAAATTAAAGAGCTGCTGAAACTTGGCGACCTG

CCGACTTCCGGTCAGATCCGCCTGTACGATGGTCGCACTGGTGAACAGTTCGAGCGTCCGGTAACCGTTGGTTACATGTACATGCTGAAACTGAACCACCTGGTCGACGACAAGATGCACGCGCGTTCCACCGGTTCTTACAGCCTGGTTACTCAGCAGCCGCTGGGTGGTAAGGCACAGTTCGGTGGTCAGCGTTTCGGGGAGATGGAAGTGTGGGCGCTGGAAGCATACGGCGCAGCATACACCCTGCAGGAAATGCTCACCGTTAAGTCTGATGACGTGAACGGTCGTACCAAGATGTATAAAAACATCGTGGACGGCAACCATCAGATGGAGCCGGGCATGCCAGAATCCTTCAACGTATTGTTGAAAGAGATTCGTTCGCTGGGTATCAACATCGAACTGGAAGACGAGTAA

Gene sequence of modified β (β(M)): Without MazF recognition sites

ATGGTTTACTCCTATACCGAGAAAAAACGTATTCGTAAGGATTTTGGTAAACGTCCGCAAGTTCTGGATGTACCTTATCTCCTTTCTATCCAGCTTGACTCGTTTCAGAAATTTATCGAGCAAGATCCTGAAGGGCAGTATGGTCTGGAAGCTGCTTTCCGTTCCGTATTCCCGATTCAGAGCTATAGCGGTAATTCCGAGCTGCAATACGTCAGCTACCGCCTTGGCGAACCGGTGTTTGACGTCCAGGAATGTCAAATCCGTGGCGTGACCTATTCCGCACCGCTGCGCGTTAAACTGCGTCTGGTGATCTATGAGCGCGAAGCGCCGGAAGGCACCGTAAAAGATATTAAAGAGCAAGAAGTCTATATGGGCGAAATTCCGCTCATGACCGATAACGGTACCTTTGTTATCAACGGTACTGAGCGTGTTATCGTTTCCCAGCTGCACCGTAGTCCGGGCGTCTTCTTTGACTCCGATAAAGGTAAAACCCACTCTTCGGGTAAAGTGCTGTATAACGCGCGTATCATCCCTTACCGTGGTTCCTGGCTGGACTTCGAATTCGATCCGAAGGATAACCTGTTCGTACGTATCGACCGTCGCCGTA

1030 AACTGCCTGCGACCATCATTCTGCGCGCCCTGAACTATACCACCGAGCAG

1031 ATCCTCGACCTGTTCTTTGAAAAAGTTATCTTTGAAATCCGTGATAATAAG

1032 CTGCAGATGGAACTGGTGCCGGAACGCCTGCGTGGTGAAACCGCATCTTT

1033 TGATATCGAAGCTAACGGTAAAGTGTACGTAGAAAAAGGCCGCCGTATCA

1034 CTGCGCGCCATATTCGCCAGCTGGAAAAAGACGACGTCAAACTGATCGAA

1035 GTCCCGGTTGAGTATATCGCAGGTAAAGTGGTTGCTAAAGACTATATTGA

1036 TGAGTCTACCGGCGAGCTGATCTGCGCAGCGAATATGGAGCTGAGCCTGG

1037 ATCTGCTGGCTAAGCTGAGCCAGTCTGGTCATAAGCGTATCGAAACGCTG

1038 TTCACCAACGATCTGGATCACGGCCCATATATCTCTGAAACCTTACGTGTC

1039 GACCCAACTAACGACCGTCTGAGCGCACTGGTAGAAATCTACCGCATGAT

1040 GCGCCCTGGCGAGCCGCCGACTCGTGAAGCAGCTGAAAGCCTGTTCGAGA

1041 ACCTGTTCTTCTCCGAAGACCGTTATGACTTGTCTGCGGTTGGTCGTATGA

1042 AGTTCAACCGTTCTCTGCTGCGCGAAGAAATCGAAGGTTCCGGTATCCTG

1043 AGCAAAGACGATATCATTGATGTTATGAAAAAGCTCATCGATATCCGTAA

1044 CGGTAAAGGCGAAGTCGATGATATCGACCACCTCGGCAACCGTCGTATCC

1045 GTTCCGTTGGCGAAATGGCGGAAAACCAGTTCCGCGTTGGCCTGGTACGT

1046 GTAGAGCGTGCGGTGAAAGAGCGTCTGTCTCTGGGCGATCTGGATACCCT

1047 GATGCCGCAGGATATGATCAACGCCAAGCCGATTTCCGCAGCAGTGAAAG

1048 AGTTCTTCGGTTCCAGCCAGCTGTCTCAGTTTATGGACCAGAATAACCCGC

1049 TGTCTGAGATTACGCATAAACGTCGTATCTCCGCACTCGGCCCAGGCGGTC

1050 TGACCCGTGAACGTGCAGGCTTCGAAGTTCGAGACGTGCACCCGACTCAC

1051 TACGGTCGCGTATGTCCAATCGAAACCCCTGAAGGTCCGAATATCGGTCT

1052 GATCAACTCTCTGTCCGTGTACGCGCAGACTAACGAATACGGCTTCCTTGA

1053 GACTCCGTATCGTAAAGTGACCGACGGTGTTGTAACTGACGAAATTCACT

1054 ACCTGTCTGCTATCGAAGAAGGCAACTACGTTATCGCCCAGGCGAACTCC

1055 AACTTGGATGAAGAAGGCCACTTCGTAGAAGACCTGGTAACTTGCCGTAG

1056 CAAAGGCGAATCCAGCTTGTTCAGCCGCGACCAGGTTGACTATATGGACG

1057 TATCCACCCAGCAGGTGGTATCCGTCGGTGCGTCCCTGATCCCGTTCCTGG

1058 AGCACGATGACGCCAACCGTGCATTGATGGGTGCGAATATGCAACGTCAG

1059 GCCGTTCCGACTCTGCGCGCTGATAAGCCGCTGGTTGGTACTGGTATGGA

1060 ACGTGCTGTTGCCGTTGACTCCGGTGTAACTGCGGTAGCTAAACGTGGTG

1061 GTGTCGTTCAGTACGTGGATGCTTCCCGTATCGTTATCAAAGTTAACGAAG

1062 ACGAGATGTATCCGGGTGAAGCAGGTATCGATATCTATAACCTGACCAAA

1063 TATACCCGTTCTAACCAGAATACCTGTATCAACCAGATGCCGTGTGTGTCT

1064 CTGGGTGAACCGGTTGAACGTGGCGACGTGCTGGCAGACGGTCCGTCCAC

1065 CGACCTCGGTGAACTGGCGCTTGGTCAGAATATGCGCGTAGCGTTCATGC

1066 CGTGGAATGGTTATAACTTCGAAGACTCCATCCTCGTATCCGAGCGTGTTG

1067 TTCAGGAAGACCGTTTCACCACCATCCATATTCAGGAACTGGCGTGTGTGT

1068 CCCGTGATACCAAGCTGGGTCCGGAAGAGATCACCGCTGATATCCCGAAC

1069 GTGGGTGAAGCTGCGCTCTCCAAACTGGATGAATCCGGTATCGTTTATATT

1070 GGTGCGGAAGTGACCGGTGGCGATATTCTGGTTGGTAAGGTAACGCCGAA

1071 AGGTGAAACTCAGCTGACCCCAGAAGAAAAACTGCTGCGTGCGATCTTCG

1072 GTGAGAAAGCCTCTGACGTTAAAGACTCTTCTCTGCGCGTACCAAACGGT

1073 GTATCCGGTACGGTTATCGACGTTCAGGTCTTTACTCGCGATGGCGTAGAA

1074 AAAGATAAACGTGCGCTGGAAATCGAAGAAATGCAGCTCAAGCAGGCGA

1075 AGAAAGACCTGTCTGAAGAACTGCAGATCCTCGAAGCGGGTCTGTTCAGC

1076 CGTATCCGTGCTGTGCTGGTAGCCGGTGGCGTTGAAGCTGAGAAGCTCGA

1077 TAAACTGCCGCGCGATCGCTGGCTGGAGCTGGGCCTGACCGACGAAGAGA

1078 AGCAAAATCAGCTGGAGCAGCTGGCTGAGCAGTATGACGAACTGAAGCA

1079 CGAGTTCGAGAAGAAACTCGAAGCGAAACGCCGCAAAATCACCCAGGGC

1080 GACGATCTGGCACCGGGCGTGCTGAAGATTGTTAAGGTATATCTGGCGGT

1081 TAAACGCCGTATCCAGCCTGGTGATAAGATGGCAGGTCGTCACGGTAATA

1082 AGGGTGTAATTTCTAAGATCAACCCGATCGAAGATATGCCTTACGATGAA

1083 AACGGTACGCCGGTAGATATCGTACTGAACCCGCTGGGCGTACCGTCTCG

1084 TATGAATATCGGTCAGATCCTCGAAACCCACCTGGGTATGGCTGCGAAAG

1085 GTATCGGCGATAAGATCAACGCCATGCTGAAGCAGCAGCAAGAAGTCGC

1086 GAAACTGCGCGAATTCATCCAGCGTGCGTACGATCTGGGCGCTGACGTTC

1087 GTCAGAAAGTTGACCTGAGTACCTTCAGCGATGAAGAAGTTATGCGTCTG

1088 GCTGAAAACCTGCGCAAAGGTATGCCAATCGCAACGCCGGTGTTCGACGG

1089 TGCGAAAGAAGCAGAAATTAAAGAGCTGCTGAAACTTGGCGACCTGCCG

1090 ACTTCCGGTCAGATCCGCCTGTACGATGGTCGCACTGGTGAGCAGTTCGA

1091 GCGTCCGGTAACCGTTGGTTATATGTATATGCTGAAACTGAACCACCTGGT

1092 CGACGATAAGATGCACGCGCGTTCCACCGGTTCTTATAGCCTGGTTACTCA

1093 GCAGCCGCTGGGTGGTAAGGCGCAGTTCGGTGGTCAGCGTTTCGGGGAGA

1094 TGGAAGTGTGGGCGCTGGAAGCATACGGCGCAGCATATACCCTGCAGGAA

1095 ATGCTCACCGTTAAGTCTGATGACGTGAACGGTCGTACCAAGATGTATAA

AAATATCGTGGACGGCAACCATCAGATGGAGCCGGGCATGCCAGAATCCTTCAACGTATTGTTGAAAGAGATTCGTTCGCTGGGTATCAATATCGAACTGGAAGACGAGTAA

Gene sequence of native β' (β'(N)): Containing 49 MazF recognition sites

GTGAAAGATTTATTAAAGTTTCTGAAAGCGCAGACTAAAACCGAAGAGTT
TGATGCGATCAAAATTGCTCTGGCTTCGCCAGACATGATCCGTTCATGGTC
TTTCGGTGAAGTTAAAAAGCCGGAAACCATCAACTACCGTACGTTCAAAC
CAGAACGTGACGGCCTTTTCTGCGCCCGTATCTTTGGGCCGGTAAAAGATT
ACGAGTGCCTGTGCGGTAAGTACAAGCGCCTGAAACACCGTGGCGTCATC
TGTGAGAAGTGCGGCGTTGAAGTGACCCAGACTAAAGTACGCCGTGAGCG
TATGGGCCACATCGAACTGGCTTCCCCGACTGCGCACATCTGGTTCCTGAA
ATCGCTGCCGTCCCGTATCGGTCTGCTGCTCGATATGCCGCTGCGCGATAT
CGAACGCGTACTGTACTTTGAATCCTATGTGGTTATCGAAGGCGGTATGAC
CAACCTGGAACGTCAGCAGATCCTGACTGAAGAGCAGTATCTGGACGCGC
TGGAAGAGTTCGGTGACGAATTCGACGCGAAGATGGGGGCGGAAGCAAT
CCAGGCTCTGCTGAAGAGCATGGATCTGGAGCAAGAGTGCGAACAGCTGC
GTGAAGAGCTGAACGAAACCAACTCCGAAACCAAGCGTAAAAAGCTGAC
CAAGCGTATCAAACTGCTGGAAGCGTTCGTTCAGTCTGGTAACAAACCAG
AGTGGATGATCCTGACCGTTCTGCCGGTACTGCCGCCAGATCTGCGTCCGC
TGGTTCCGCTGGATGGTGGTCGTTTCGCGACTTCTGACCTGAACGATCTGT
ATCGTCGCGTCATTAACCGTAACAACCGTCTGAAACGTCTGCTGGATCTGG
CTGCGCCGGACATCATCGTACGTAACGAAAAACGTATGCTGCAGGAAGCG
GTAGACGCCCTGCTGGATAACGGTCGTCGCGGTCGTGCGATCACCGGTTC
TAACAAGCGTCCTCTGAAATCTTTGGCCGACATGATCAAAGGTAAACAGG

1122 GTCGTTTCCGTCAGAACCTGCTCGGTAAGCGTGTTGACTACTCCGGTCGTT

1123 CTGTAATCACCGTAGGTCCATACCTGCGTCTGCATCAGTGCGGTCTGCCGA

1124 AGAAAATGGCACTGGAGCTGTTCAAACCGTTCATCTACGGCAAGCTGGAA

1125 CTGCGTGGTCTTGCTACCACCATTAAAGCTGCGAAGAAAATGGTTGAGCG

1126 CGAAGAAGCTGTCGTTTGGGATATCCTGGACGAAGTTATCCGCGAACACC

1127 CGGTACTGCTGAACCGTGCACCGACTCTGCACCGTCTGGGTATCCAGGCA

1128 TTTGAACCGGTACTGATCGAAGGTAAAGCTATCCAGCTGCACCCGCTGGT

1129 TTGTGCGGCATATAACGCCGACTTCGATGGTGACCAGATGGCTGTTCACGT

1130 ACCGCTGACGCTGGAAGCCCAGCTGGAAGCGCGTGCGCTGATGATGTCTA

1131 CCAACAACATCCTGTCCCCGGCGAACGGCGAACCAATCATCGTTCCGTCT

1132 CAGGACGTTGTACTGGGTCTGTACTACATGACCCGTGACTGTGTTAACGCC

1133 AAAGGCGAAGGCATGGTGCTGACTGGCCCGAAAGAAGCAGAACGTCTGT

1134 ATCGCTCTGGTCTGGCTTCTCTGCATGCGCGCGTTAAAGTGCGTATCACCG

1135 AGTATGAAAAAGATGCTAACGGTGAATTAGTAGCGAAAACCAGCCTGAA

1136 AGACACGACTGTTGGCCGTGCCATTCTGTGGATGATTGTACCGAAAGGTC

1137 TGCCTTACTCCATCGTCAACCAGGCGCTGGGTAAAAAAGCAATCTCCAAA

1138 ATGCTGAACACCTGCTACCGCATTCTCGGTCTGAAACCGACCGTTATTTTT

1139 GCGGACCAGATCATGTACACCGGCTTCGCCTATGCAGCGCGTTCTGGTGC

1140 ATCTGTTGGTATCGATGACATGGTCATCCCGGAGAAGAAACACGAAATCA

1141 TCTCCGAGGCAGAAGCAGAAGTTGCTGAAATTCAGGAGCAGTTCCAGTCT

1142 GGTCTGGTAACTGCGGGCGAACGCTACAACAAAGTTATCGATATCTGGGC

1143 TGCGGCGAACGATCGTGTATCCAAAGCGATGATGGATAACCTGCAAACTG

1144 AAACCGTGATTAACCGTGACGGTCAGGAAGAGAAGCAGGTTTCCTTCAAC

1145 AGCATCTACATGATGGCCGACTCCGGTGCGCGTGGTTCTGCGGCACAGAT

1146 TCGTCAGCTTGCTGGTATGCGTGGTCTGATGGCGAAGCCGGATGGCTCCAT

1147 CATCGAAACGCCAATCACCGCGAACTTCCGTGAAGGTCTGAACGTACTCC

1148 AGTACTTCATCTCCACCCACGGTGCTCGTAAAGGTCTGGCGGATACCGCA

1149 CTGAAAACTGCGAACTCCGGTTACCTGACTCGTCGTCTGGTTGACGTGGCG

1150 CAGGACCTGGTGGTTACCGAAGACGATTGTGGTACCCATGAAGGTATCAT

1151 GATGACTCCGGTTATCGAGGGTGGTGACGTTAAAGAGCCGCTGCGCGATC

1152 GCGTACTGGGTCGTGTAACTGCTGAAGACGTTCTGAAGCCGGGTACTGCT

1153 GATATCCTCGTTCCGCGCAACACGCTGCTGCACGAACAGTGGTGTGACCT

1154 GCTGGAAGAGAACTCTGTCGACGCGGTTAAAGTACGTTCTGTTGTATCTTG

1155 TGACACCGACTTTGGTGTATGTGCGCACTGCTACGGTCGTGACCTGGCGCG

1156 TGGCCACATCATCAACAAGGGTGAAGCAATCGGTGTTATCGCGGCACAGT

1157 CCATCGGTGAACCGGGTACACAGCTGACCATGCGTACGTTCCACATCGGT

1158 GGTGCGGCATCTCGTGCGGCTGCTGAATCCAGCATCCAAGTGAAAAACAA

1159 AGGTAGCATCAAGCTCAGCAACGTGAAGTCGGTTGTGAACTCCAGCGGTA

1160 AACTGGTTATCACTTCCCGTAATACTGAACTGAAACTGATCGACGAATTCG

1161 GTCGTACTAAAGAAAGCTACAAAGTACCTTACGGTGCGGTACTGGCGAAA

1162 GGCGATGGCGAACAGGTTGCTGGCGGCGAAACCGTTGCAAACTGGGACCC

1163 GCACACCATGCCGGTTATCACCGAAGTAAGCGGTTTTGTACGCTTTACTGA

1164 CATGATCGACGGCCAGACCATTACGCGTCAGACCGACGAACTGACCGGTC

1165 TGTCTTCGCTGGTGGTTCTGGATTCCGCAGAACGTACCGCAGGTGGTAAA

GATCTGCGTCCGGCACTGAAAATCGTTGATGCTCAGGGTAACGACGTTCT
GATCCCAGGTACCGATATGCCAGCGCAGTACTTCCTGCCGGGTAAAGCGA
TTGTTCAGCTGGAAGATGGCGTACAGATCAGCTCTGGTGACACCCTGGCG
CGTATTCCGCAGGAATCCGGCGGTACCAAGGACATCACCGGTGGTCTGCC
GCGCGTTGCGGACCTGTTCGAAGCACGTCGTCCGAAAGAGCCGGCAATCC
TGGCTGAAATCAGCGGTATCGTTTCCTTCGGTAAAGAAACCAAAGGTAAA
CGTCGTCTGGTTATCACCCCGGTAGACGGTAGCGATCCGTACGAAGAGAT
GATTCCGAAATGGCGTCAGCTCAACGTGTTCGAAGGTGAACGTGTAGAAC
GTGGTGACGTAATTTCCGACGGTCCGGAAGCGCCGCACGACATTCTGCGT
CTGCGTGGTGTTCATGCTGTTACTCGTTACATCGTTAACGAAGTACAGGAC
GTATACCGTCTGCAGGGCGTTAAGATTAACGATAAACACATCGAAGTTAT
CGTTCGTCAGATGCTGCGTAAAGCTACCATCGTTAACGCGGGTAGCTCCG
ACTTCCTGGAAGGCGAACAGGTTGAATACTCTCGCGTCAAGATCGCAAAC
CGCGAACTGGAAGCGAACGGCAAAGTGGGTGCAACTTACTCCCGCGATCT
GCTGGGTATCACCAAAGCGTCTCTGGCAACCGAGTCCTTCATCTCCGCGGC
ATCGTTCCAGGAGACCACTCGCGTGCTGACCGAAGCAGCCGTTGCGGGCA
AACGCGACGAACTGCGCGGCCTGAAAGAGAACGTTATCGTGGGTCGTCTG
ATCCCGGCAGGTACCGGTTACGCGTACCACCAGGATCGTATGCGTCGCCG
TGCTGCGGGTGAAGCTCCGGCTGCACCGCAGGTGACTGCAGAAGACGCAT
CTGCCAGCCTGGCAGAACTGCTGAACGCAGGTCTGGGCGGTTCTGATAAC
GAGTAA

Gene sequence of modified β' (β'(M)): Without MazF recognition sites

1188 GTGAAAGATTTATTAAAGTTTCTGAAAGCGCAGACTAAAACCGAAGAGTT

1189 TGATGCGATCAAAATTGCTCTGGCTTCGCCAGATATGATCCGTTCATGGTC

1190 TTTCGGTGAAGTTAAAAAGCCGGAAACCATCAACTACCGTACGTTCAAAC

1191 CAGAACGTGACGGCCTTTTCTGCGCCCGTATCTTTGGGCCGGTAAAAGATT

1192 ACGAGTGCCTGTGCGGTAAGTATAAGCGCCTGAAGCACCGTGGCGTCATC

1193 TGTGAGAAGTGCGGCGTTGAAGTGACCCAGACTAAAGTACGCCGTGAGCG

1194 TATGGGCCATATCGAACTGGCTTCCCCGACTGCGCATATCTGGTTCCTGAA

1195 ATCGCTGCCGTCCCGTATCGGTCTGCTGCTCGATATGCCGCTGCGCGATAT

1196 CGAACGCGTACTGTACTTTGAATCCTATGTGGTTATCGAAGGCGGTATGAC

1197 CAACCTGGAACGTCAGCAGATCCTGACTGAAGAGCAGTATCTGGACGCGC

1198 TGGAAGAGTTCGGTGACGAATTCGACGCGAAGATGGGGGCGGAAGCAAT

1199 CCAGGCTCTGCTGAAGAGCATGGATCTGGAGCAAGAGTGCGAGCAGCTGC

1200 GTGAAGAGCTGAACGAAACCAACTCCGAAACCAAGCGTAAAAAGCTGAC

1201 CAAGCGTATCAAACTGCTGGAAGCGTTCGTTCAGTCTGGTAATAAACCAG

1202 AGTGGATGATCCTGACCGTTCTGCCGGTACTGCCGCCAGATCTGCGTCCGC

1203 TGGTTCCGCTGGATGGTGGTCGTTTCGCGACTTCTGACCTGAACGATCTGT

1204 ATCGTCGCGTCATTAACCGTAATAACCGTCTGAAACGTCTGCTGGATCTGG

1205 CTGCGCCGGATATCATCGTACGTAACGAAAAACGTATGCTGCAGGAAGCG

1206 GTAGACGCCCTGCTGGATAACGGTCGTCGCGGTCGTGCGATCACCGGTTC

1207 TAATAAGCGTCCTCTGAAATCTTTGGCCGATATGATCAAAGGTAAGCAGG

1208 GTCGTTTCCGTCAGAACCTGCTCGGTAAGCGTGTTGACTACTCCGGTCGTT

1209 CTGTAATCACCGTAGGTCCATACCTGCGTCTGCATCAGTGCGGTCTGCCGA

1210 AGAAAATGGCACTGGAGCTGTTCAAACCGTTCATCTACGGCAAGCTGGAA

1211 CTGCGTGGTCTTGCTACCACCATTAAAGCTGCGAAGAAAATGGTTGAGCG

1212 CGAAGAAGCTGTCGTTTGGGATATCCTGGACGAAGTTATCCGCGAGCACC

1213 CGGTACTGCTGAACCGTGCACCGACTCTGCACCGTCTGGGTATCCAGGCA

1214 TTTGAACCGGTACTGATCGAAGGTAAAGCTATCCAGCTGCACCCGCTGGT

1215 TTGTGCGGCATATAACGCCGACTTCGATGGTGACCAGATGGCTGTTCACGT

1216 ACCGCTGACGCTGGAAGCCCAGCTGGAAGCGCGTGCGCTGATGATGTCTA

1217 CCAATAATATCCTGTCCCCGGCGAACGGCGAACCAATCATCGTTCCGTCTC

1218 AGGACGTTGTACTGGGTCTGTACTATATGACCCGTGACTGTGTTAACGCCA

1219 AAGGCGAAGGCATGGTGCTGACTGGCCCGAAAGAAGCAGAACGTCTGTA

1220 TCGCTCTGGTCTGGCTTCTCTGCATGCGCGCGTTAAAGTGCGTATCACCGA

1221 GTATGAAAAAGATGCTAACGGTGAATTAGTAGCGAAAACCAGCCTGAAA

1222 GATACGACTGTTGGCCGTGCCATTCTGTGGATGATTGTACCGAAAGGTCTG

1223 CCTTACTCCATCGTCAACCAGGCGCTGGGTAAAAAAGCAATCTCCAAAAT

1224 GCTGAATACCTGCTACCGCATTCTCGGTCTGAAACCGACCGTTATTTTTGC

1225 GGACCAGATCATGTATACCGGCTTCGCCTATGCAGCGCGTTCTGGTGCATC

1226 TGTTGGTATCGATGATATGGTCATCCCGGAGAAGAAGCACGAAATCATCT

1227 CCGAGGCAGAAGCAGAAGTTGCTGAAATTCAGGAGCAGTTCCAGTCTGGT

1228 CTGGTAACTGCGGGCGAACGCTATAATAAAGTTATCGATATCTGGGCTGC

1229 GGCGAACGATCGTGTATCCAAAGCGATGATGGATAACCTGCAAACTGAAA

1230 CCGTGATTAACCGTGACGGTCAGGAAGAGAAGCAGGTTTCCTTCAATAGC

1231 ATCTATATGATGGCCGACTCCGGTGCGCGTGGTTCTGCGGCGCAGATTCGT

1232 CAGCTTGCTGGTATGCGTGGTCTGATGGCGAAGCCGGATGGCTCCATCAT

1233 CGAAACGCCAATCACCGCGAACTTCCGTGAAGGTCTGAACGTACTCCAGT

1234 ACTTCATCTCCACCCACGGTGCTCGTAAAGGTCTGGCGGATACCGCACTG

1235 AAAACTGCGAACTCCGGTTACCTGACTCGTCGTCTGGTTGACGTGGCGCA

1236 GGACCTGGTGGTTACCGAAGACGATTGTGGTACCCATGAAGGTATCATGA

1237 TGACTCCGGTTATCGAGGGTGGTGACGTTAAAGAGCCGCTGCGCGATCGC

1238 GTACTGGGTCGTGTAACTGCTGAAGACGTTCTGAAGCCGGGTACTGCTGA

1239 TATCCTCGTTCCGCGCAATACGCTGCTGCACGAGCAGTGGTGTGACCTGCT

1240 GGAAGAGAACTCTGTCGACGCGGTTAAAGTACGTTCTGTTGTATCTTGTGA

1241 TACCGACTTTGGTGTATGTGCGCACTGCTACGGTCGTGACCTGGCGCGTGG

1242 CCATATCATCAATAAGGGTGAAGCAATCGGTGTTATCGCGGCGCAGTCCA

1243 TCGGTGAACCGGGTACCCAGCTGACCATGCGTACGTTCCATATCGGTGGT

1244 GCGGCATCTCGTGCGGCTGCTGAATCCAGCATCCAAGTGAAAAATAAAGG

1245 TAGCATCAAGCTCAGCAACGTGAAGTCGGTTGTGAACTCCAGCGGTAAAC

1246 TGGTTATCACTTCCCGTAATACTGAACTGAAACTGATCGACGAATTCGGTC

1247 GTACTAAAGAAAGCTATAAAGTACCTTACGGTGCGGTACTGGCGAAAGGC

1248 GATGGCGAGCAGGTTGCTGGCGGCGAAACCGTTGCAAACTGGGACCCGCA

1249 TACCATGCCGGTTATCACCGAAGTAAGCGGTTTTGTACGCTTTACTGATAT

1250 GATCGACGGCCAGACCATTACGCGTCAGACCGACGAACTGACCGGTCTGT

1251 CTTCGCTGGTGGTTCTGGATTCCGCAGAACGTACCGCAGGTGGTAAAGAT

1252 CTGCGTCCGGCACTGAAAATCGTTGATGCTCAGGGTAACGACGTTCTGAT

1253 CCCAGGTACCGATATGCCAGCGCAGTACTTCCTGCCGGGTAAAGCGATTG

TTCAGCTGGAAGATGGCGTGCAGATCAGCTCTGGTGATACCCTGGCGCGTATTCCGCAGGAATCCGGCGGTACCAAGGATATCACCGGTGGTCTGCCGCGCGTTGCGGACCTGTTCGAAGCACGTCGTCCGAAAGAGCCGGCAATCCTGGCTGAAATCAGCGGTATCGTTTCCTTCGGTAAAGAAACCAAAGGTAAACGTCGTCTGGTTATCACCCCGGTAGACGGTAGCGATCCGTACGAAGAGATGATTCCGAAATGGCGTCAGCTCAACGTGTTCGAAGGTGAACGTGTAGAACGTGGTGACGTAATTTCCGACGGTCCGGAAGCGCCGCACGATATTCTGCGTCTGCGTGGTGTTCATGCTGTTACTCGTTATATCGTTAACGAAGTGCAGGACGTATACCGTCTGCAGGGCGTTAAGATTAACGATAAGCATATCGAAGTTATCGTTCGTCAGATGCTGCGTAAAGCTACCATCGTTAACGCGGGTAGCTCCGACTTCCTGGAAGGCGAGCAGGTTGAATACTCTCGCGTCAAGATCGCAAACCGCGAACTGGAAGCGAACGGCAAAGTGGGTGCAACTTACTCCCGCGATCTGCTGGGTATCACCAAAGCGTCTCTGGCAACCGAGTCCTTCATCTCCGCGGCATCGTTCCAGGAGACCACTCGCGTGCTGACCGAAGCAGCCGTTGCGGGCAAACGCGACGAACTGCGCGGCCTGAAAGAGAACGTTATCGTGGGTCGTCTGATCCCGGCAGGTACCGGTTACGCGTACCACCAGGATCGTATGCGTCGCCGTGCTGCGGGTGAAGCTCCGGCTGCACCGCAGGTGACTGCAGAAGACGCATCTGCCAGCCTGGCAGAACTGCTGAACGCAGGTCTGGGCGGTTCTGATAACGAGTAA

Gene sequence of native EF-Ts (EF-Ts(N)): Containing 9 MazF recognition sites

ATGGCTGAAATTACCGCATCCCTGGTAAAAGAGCTGCGTGAGCGTACTGG

CGCAGGCATGATGGATTGCAAAAAAGCACTGACTGAAGCTAACGGCGACATCGAGCTGGCAATCGAAAACATGCGTAAGTCCGGTGCTATTAAAGCAGCGAAAAAAGCAGGCAACGTTGCTGCTGACGGCGTGATCAAAACCAAAATCGACGGCAACTACGGCATCATTCTGGAAGTTAACTGCCAGACTGACTTCGTTGCAAAAGACGCTGGTTTCCAGGCGTTCGCAGACAAAGTTCTGGACGCAGCTGTTGCTGGCAAAATCACTGACGTTGAAGTTCTGAAAGCACAGTTCGAAGAAGAACGTGTTGCGCTGGTAGCGAAAATTGGTGAAAACATCAACATTCGCCGCGTTGCTGCGCTGGAAGGCGACGTTCTGGGTTCTTATCAGCACGGTGCGCGTATCGGCGTTCTGGTTGCTGCTAAAGGCGCTGACGAAGAGCTGGTTAAACACATCGCTATGCACGTTGCTGCAAGCAAGCCAGAATTCATCAAACCGGAAGACGTATCCGCTGAAGTGGTAGAAAAAGAATACCAGGTACAGCTGGATATCGCGATGCAGTCTGGTAAGCCGAAAGAAATCGCAGAGAAAATGGTTGAAGGCCGCATGAAGAAATTCACCGGCGAAGTTTCTCTGACCGGTCAGCCGTTCGTTATGGAACCAAGCAAAACTGTTGGTCAGCTGCTGAAAGAGCATAACGCTGAAGTGACTGGCTTCATCCGCTTCGAAGTGGGTGAAGGCATCGAGAAAGTTGAGACTGACTTTGCAGCAGAAGTTGCTGCGATGTCCAAGCAGTCTTAA

Gene sequence of modified EF-Ts (EF-Ts(M)): Without MazF recognition sites

ATGGCTGAAATTACCGCATCCCTGGTAAAAGAGCTGCGTGAGCGTACTGGCGCAGGCATGATGGATTGCAAAAAAGCACTGACTGAAGCTAACGGCGATATCGAGCTGGCAATCGAAAATATGCGTAAGTCCGGTGCTATTAAAGCAGCGAAAAAAGCAGGCAACGTTGCTGCTGACGGCGTGATCAAAACCAAAATC

GACGGCAACTACGGCATCATTCTGGAAGTTAACTGCCAGACTGACTTCGTTGCAAAAGACGCTGGTTTCCAGGCGTTCGCAGATAAAGTTCTGGACGCAGCTGTTGCTGGCAAAATCACTGACGTTGAAGTTCTGAAAGCGCAGTTCGAAGAAGAACGTGTTGCGCTGGTAGCGAAAATTGGTGAAAATATCAATATTCGCCGCGTTGCTGCGCTGGAAGGCGACGTTCTGGGTTCTTATCAGCACGGTGCGCGTATCGGCGTTCTGGTTGCTGCTAAAGGCGCTGACGAAGAGCTGGTTAAGCATATCGCTATGCACGTTGCTGCAAGCAAGCCAGAATTCATCAAACCGGAAGACGTATCCGCTGAAGTGGTAGAAAAAGAATACCAGGTGCAGCTGGATATCGCGATGCAGTCTGGTAAGCCGAAAGAAATCGCAGAGAAAATGGTTGAAGGCCGCATGAAGAAATTCACCGGCGAAGTTTCTCTGACCGGTCAGCCGTTCGTTATGGAACCAAGCAAAACTGTTGGTCAGCTGCTGAAAGAGCATAACGCTGAAGTGACTGGCTTCATCCGCTTCGAAGTGGGTGAAGGCATCGAGAAAGTTGAGACTGACTTTGCAGCAGAAGTTGCTGCGATGTCCAAGCAGTCTTAA

Gene sequence of native L17 (L17(N)): Containing 4 MazF recognition sites

ATGCGCCATCGTAAGAGTGGTCGTCAACTGAACCGCAACAGCAGCCATCGCCAGGCTATGTTCCGCAATATGGCAGGTTCACTGGTTCGTCATGAAATCATCAAGACGACTCTGCCTAAAGCGAAAGAGCTGCGCCGCGTAGTTGAGCCGCTGATTACTCTTGCCAAGACTGATAGCGTTGCTAATCGTCGTCTGGCATTCGCCCGTACTCGTGATAACGAGATCGTGGCAAAACTGTTTAACGAACTGGGCCCGCGTTTCGCGAGCCGTGCCGGTGGTTACACTCGTATTCTGAAGTGTGGC

TTCCGTGCAGGCGACAACGCGCCGATGGCTTACATCGAGCTGGTTGATCG
TTCAGAGAAAGCAGAAGCTGCTGCAGAGTAA

Gene sequence of modified L17 (L17(M)): Without MazF recognition sites

ATGCGCCATCGTAAGAGTGGTCGTCAACTGAACCGCAATAGCAGCCATCG
CCAGGCTATGTTCCGCAATATGGCAGGTTCACTGGTTCGTCATGAAATCAT
CAAGACGACTCTGCCTAAAGCGAAAGAGCTGCGCCGCGTAGTTGAGCCGC
TGATTACTCTTGCCAAGACTGATAGCGTTGCTAATCGTCGTCTGGCATTCG
CCCGTACTCGTGATAACGAGATCGTGGCAAAACTGTTTAACGAACTGGGC
CCGCGTTTCGCGAGCCGTGCCGGTGGTTATACTCGTATTCTGAAGTGTGGC
TTCCGTGCAGGCGATAACGCGCCGATGGCTTATATCGAGCTGGTTGATCGT
TCAGAGAAAGCAGAAGCTGCTGCAGAGTAA

Gene sequence of native S9 (S9(N)): Containing 3 MazF recognition sites

ATGGCTGAAAATCAATACTACGGCACTGGTCGCCGCAAAAGTTCCGCAGC
TCGCGTTTTCATCAAACCGGGCAACGGTAAAATCGTAATCAACCAACGTT
CTCTGGAACAGTACTTCGGTCGTGAAACTGCCCGCATGGTAGTTCGTCAGC
CGCTGGAACTGGTCGACATGGTTGAGAAACTGGACCTGTACATCACCGTT
AAAGGTGGTGGTATCTCTGGTCAGGCTGGTGCGATCCGTCACGGTATCAC
CCGCGCTCTGATGGAATACGACGAGTCCCTGCGTTCTGAACTGCGTAAAG
CTGGCTTCGTTACTCGTGACGCTCGTCAGGTTGAACGTAAGAAAGTCGGTC
TGCGTAAAGCACGTCGTCGTCCGCAGTTCTCCAAACGTTAA

Gene sequence of modified S9 (S9(M)): Without MazF recognition sites

ATGGCTGAAAATCAATACTACGGCACTGGTCGCCGCAAAAGTTCCGCAGC
TCGCGTTTTCATCAAACCGGGCAACGGTAAAATCGTAATCAACCAACGTT
CTCTGGAGCAGTACTTCGGTCGTGAAACTGCCCGCATGGTAGTTCGTCAGC
CGCTGGAACTGGTCGATATGGTTGAGAAACTGGACCTGTATATCACCGTT
AAAGGTGGTGGTATCTCTGGTCAGGCTGGTGCGATCCGTCACGGTATCAC
CCGCGCTCTGATGGAATACGACGAGTCCCTGCGTTCTGAACTGCGTAAAG
CTGGCTTCGTTACTCGTGACGCTCGTCAGGTTGAACGTAAGAAAGTCGGTC
TGCGTAAAGCACGTCGTCGTCCGCAGTTCTCCAAACGTTAA

Gene sequence of native S20 (S20(N)): Containing 7 MazF recognition sites

ATGGCTAATATCAAATCAGCTAAGAAGCGCGCCATTCAGTCTGAAAAGGC
TCGTAAGCACAACGCAAGCCGTCGCTCTATGATGCGTACTTTCATCAAGA
AAGTATACGCAGCTATCGAAGCTGGCGACAAAGCTGCTGCACAGAAAGC
ATTTAACGAAATGCAACCGATCGTGGACCGTCAGGCTGCTAAAGGTCTGA
TCCACAAAAACAAAGCTGCACGTCATAAGGCTAACCTGACTGCACAGATC
AACAAACTGGCTTAA

Gene sequence of modified S20 (S20(M)): Without MazF recognition sites

ATGGCTAATATCAAATCAGCTAAGAAGCGCGCCATTCAGTCTGAAAAGGC
TCGTAAGCATAACGCAAGCCGTCGCTCTATGATGCGTACTTTCATCAAGA
AAGTATACGCAGCTATCGAAGCTGGCGATAAAGCTGCTGCGCAGAAAGCA
TTTAACGAAATGCAACCGATCGTGGACCGTCAGGCTGCTAAAGGTCTGAT
CCATAAAAATAAAGCTGCACGTCATAAGGCTAACCTGACTGCGCAGATCA

ATAAACTGGCTTAA

Gene sequence of native BktB (BktB(N)): Containing 8 MazF recognition sites

ATGACCCGTGAAGTTGTTGTTGTTTCTGGTGTTCGTACCGCTATCGGTACT
TTCGGTGGTTCTCTGAAAGACGTTGCTCCGGCTGAACTGGGTGCTCTGGTT
GTTCGTGAAGCTCTGGCTCGTGCTCAGGTTTCTGGTGACGACGTTGGCCAC
GTTGTCTTCGGCAACGTTATCCAGACCGAACCGCGTGACATGTACCTGGGT
CGTGTTGCTGCTGTTAACGGTGGTGTTACCATCAACGCTCCGGCTCTGACC
GTTAACCGTCTGTGCGGTTCTGGTCTGCAGGCTATCGTTTCTGCTGCTCAG
ACCATCCTGCTGGGTGACACCGACGTTGCTATCGGTGGTGGTGCTGAATCT
ATGTCTCGTGCTCCGTACCTGGCTCCGGCTGCTCGTTGGGGTGCTCGTATG
GGTGACGCTGGTCTGGTTGACATGATGCTGGGTGCTCTGCACGACCCGTTC
CACCGTATCCACATGGGTGTTACCGCTGAAAACGTTGCTAAAGAATACGA
CATCTCTCGTGCTCAGCAGGACGAAGCTGCTCTGGAATCTCACCGTCGTGC
TTCTGCTGCTATCAAAGCTGGTTACTTCAAAGACCAGATCGTTCCAGTTGT
AAGCAAAGGTCGTAAAGGTGACGTTACCTTCGACACCGACGAACACGTTC
GTCACGACGCTACCATCGACGACATGACCAAACTGCGTCCGGTTTTCGTTA
AAGAAAACGGTACTGTTACCGCTGGTAACGCTTCTGGTCTGAACGACGCT
GCTGCTGCTGTTGTTATGATGGAACGTGCTGAAGCTGAACGTCGTGGTCTG
AAACCGCTGGCTCGTCTGGTTTCTTACGGTCACGCTGGTGTTGACCCGAAA
GCTATGGGTATCGGTCCGGTTCCGGCTACCAAAATCGCTCTGGAACGTGCT
GGTCTGCAGGTTTCTGACCTGGACGTTATCGAAGCGAATGAAGCGTTCGC

TGCTCAGGCGTGCGCTGTTACCAAAGCTCTGGGTCTGGACCCGGCTAAAG
TTAACCCGAACGGTTCTGGTATCTCTCTGGGTCACCCGATCGGTGCTACCG
GTGCTCTGATCACCGTTAAAGCTCTGCACGAACTGAACCGTGTTCAGGGTC
GTTACGCTCTGGTTACCATGTGCATCGGTGGTGGTCAGGGTATCGCTGCTA
TCTTCGAACGTATCTAA

Gene sequence of modified BktB (BktB(M)): Without MazF recognition sites

ATGACCCGTGAAGTTGTTGTTGTTTCTGGTGTTCGTACCGCTATCGGTACT
TTCGGTGGTTCTCTGAAAGACGTTGCTCCGGCTGAACTGGGTGCTCTGGTT
GTTCGTGAAGCTCTGGCTCGTGCTCAGGTTTCTGGTGACGACGTTGGCCAC
GTTGTCTTCGGCAACGTTATCCAGACCGAACCGCGTGATATGTACCTGGGT
CGTGTTGCTGCTGTTAACGGTGGTGTTACCATCAACGCTCCGGCTCTGACC
GTTAACCGTCTGTGCGGTTCTGGTCTGCAGGCTATCGTTTCTGCTGCTCAG
ACCATCCTGCTGGGTGATACCGACGTTGCTATCGGTGGTGGTGCTGAATCT
ATGTCTCGTGCTCCGTACCTGGCTCCGGCTGCTCGTTGGGGTGCTCGTATG
GGTGACGCTGGTCTGGTTGATATGATGCTGGGTGCTCTGCACGACCCGTTC
CACCGTATCCATATGGGTGTTACCGCTGAAAACGTTGCTAAAGAATACGA
TATCTCTCGTGCTCAGCAGGACGAAGCTGCTCTGGAATCTCACCGTCGTGC
TTCTGCTGCTATCAAAGCTGGTTACTTCAAAGACCAGATCGTTCCAGTTGT
AAGCAAAGGTCGTAAAGGTGACGTTACCTTCGATACCGACGAGCACGTTC
GTCACGACGCTACCATCGACGATATGACCAAACTGCGTCCGGTTTTCGTTA
AAGAAAACGGTACTGTTACCGCTGGTAACGCTTCTGGTCTGAACGACGCT
GCTGCTGCTGTTGTTATGATGGAACGTGCTGAAGCTGAACGTCGTGGTCTG

AAACCGCTGGCTCGTCTGGTTTCTTACGGTCACGCTGGTGTTGACCCGAAA
GCTATGGGTATCGGTCCGGTTCCGGCTACCAAAATCGCTCTGGAACGTGCT
GGTCTGCAGGTTTCTGACCTGGACGTTATCGAAGCGAATGAAGCGTTCGC
TGCTCAGGCGTGCGCTGTTACCAAAGCTCTGGGTCTGGACCCGGCTAAAG
TTAACCCGAACGGTTCTGGTATCTCTCTGGGTCACCCGATCGGTGCTACCG
GTGCTCTGATCACCGTTAAAGCTCTGCACGAACTGAACCGTGTTCAGGGTC
GTTACGCTCTGGTTACCATGTGCATCGGTGGTGGTCAGGGTATCGCTGCTA
TCTTCGAACGTATCTAA

Gene sequence of native FadB (FadB(N)): Containing 22 MazF recognition sites

ATGCTTTACAAAGGCGACACCCTGTACCTTGACTGGCTGGAAGATGGCAT
TGCCGAACTGGTATTTGATGCCCCAGGTTCAGTTAATAAACTCGACACTGC
GACCGTCGCCAGCCTCGGCGAGGCCATCGGCGTGCTGGAACAGCAATCAG
ATCTAAAAGGGCTGCTGCTGCGTTCGAACAAAGCAGCCTTTATCGTCGGT
GCTGATATCACCGAATTTTTGTCCCTGTTCCTCGTTCCTGAAGAACAGTTA
AGTCAGTGGCTGCACTTTGCCAATAGCGTGTTTAATCGCCTGGAAGATCTG
CCGGTGCCGACCATTGCTGCCGTCAATGGCTATGCGCTGGGCGGTGGCTG
CGAATGCGTGCTGGCGACCGATTATCGTCTGGCGACGCCGGATCTGCGCA
TCGGTCTGCCGGAAACCAAACTGGGCATCATGCCTGGCTTTGGCGGTTCTG
TACGTATGCCACGTATGCTGGGCGCTGACAGTGCGCTGGAAATCATTGCC
GCCGGTAAAGATGTCGGCGCGGATCAGGCGCTGAAAATCGGTCTGGTGGA
TGGCGTAGTCAAAGCAGAAAAACTGGTTGAAGGCGCAAAGGCGGTTTTAC

1430 GCCAGGCCATTAACGGCGACCTCGACTGGAAAGCAAAACGTCAGCCGAA

1431 GCTGGAACCACTAAAACTGAGCAAGATTGAAGCCACCATGAGCTTCACCA

1432 TCGCTAAAGGGATGGTCGCACAAACAGCGGGGAAACATTATCCGGCCCCC

1433 ATCACCGCAGTAAAAACCATTGAAGCTGCGGCCCGTTTTGGTCGTGAAGA

1434 AGCCTTAAACCTGGAAAACAAAAGTTTTGTCCCGCTGGCGCATACCAACG

1435 AAGCCCGCGCACTGGTCGGCATTTTCCTTAACGATCAATATGTAAAAGGC

1436 AAAGCGAAGAAACTCACCAAAGACGTTGAAACCCCGAAACAGGCCGCGG

1437 TGCTGGGTGCAGGCATTATGGGCGGCGGCATCGCTTACCAGTCTGCGTGG

1438 AAAGGCGTGCCGGTTGTCATGAAAGATATCAACGACAAGTCGTTAACCCT

1439 CGGCATGACCGAAGCCGCGAAACTGCTGAACAAGCAGCTTGAGCGCGGC

1440 AAGATCGATGGTCTGAAACTGGCTGGCGTGATCTCCACAATCCACCCAAC

1441 GCTCGACTACGCCGGATTTGACCGCGTGGATATTGTGGTAGAAGCGGTTG

1442 TTGAAAACCCGAAAGTGAAAAAAGCCGTACTGGCAGAAACCGAACAAAA

1443 AGTACGCCAGGATACCGTGCTGGCGTCTAACACTTCAACCATTCCTATCAG

1444 CGAACTGGCCAACGCGCTGGAACGCCCGGAAAACTTCTGCGGGATGCACT

1445 TCTTTAACCCGGTCCACCGAATGCCGTTGGTAGAAATTATTCGCGGCGAG

1446 AAAAGCTCCGACGAAACCATCGCGAAAGTTGTCGCCTGGGCGAGCAAGAT

1447 GGGCAAGACGCCGATTGTGGTTAACGACTGCCCCGGCTTCTTTGTTAACCG

1448 CGTGCTGTTCCCGTATTTCGCCGGTTTCAGCCAGCTGCTGCGCGACGGCGC

1449 GGATTTCCGCAAGATCGACAAAGTGATGGAAAAACAGTTTGGCTGGCCGA

1450 TGGGCCCGGCATATCTGCTGGACGTTGTGGGCATTGATACCGCGCATCAC

1451 GCTCAGGCTGTCATGGCAGCAGGCTTCCCGCAGCGGATGCAGAAAGATTA

CCGCGATGCCATCGACGCGCTGTTTGATGCCAACCGCTTTGGTCAGAAGAACGGCCTCGGTTTCTGGCGTTATAAAGAAGACAGCAAAGGTAAGCCGAAGAAAGAAGAAGACGCCGCCGTTGAAGACCTGCTGGCAGAAGTGAGCCAGCCGAAGCGCGATTTCAGCGAAGAAGAGATTATCGCCCGCATGATGATCCCGATGGTCAACGAAGTGGTGCGCTGTCTGGAGGAAGGCATTATCGCCACTCCGGCGGAAGCGGATATGGCGCTGGTCTACGGCCTGGGCTTCCCTCCGTTCCACGGCGGCGCGTTCCGCTGGCTGGACACCCTCGGTAGCGCAAAATACCTCGATATGGCACAGCAATATCAGCACCTCGGCCCGCTGTATGAAGTGCCGGAAGGTCTGCGTAATAAAGCGCGTCATAACGAACCGTACTATCCTCCGGTTGAGCCAGCCCGTCCGGTTGGCGACCTGAAAACGGCTTAA

Gene sequence of modified FadB (FadB(M)): Without MazF recognition sites

ATGCTTTATAAAGGCGATACCCTGTACCTTGACTGGCTGGAAGATGGCATTGCCGAACTGGTATTTGATGCCCCAGGTTCAGTTAATAAACTCGATACTGCGACCGTCGCCAGCCTCGGCGAGGCCATCGGCGTGCTGGAGCAGCAATCAGATCTAAAAGGGCTGCTGCTGCGTTCGAATAAAGCAGCCTTTATCGTCGGTGCTGATATCACCGAATTTTTGTCCCTGTTCCTCGTTCCTGAAGAGCAGTTAAGTCAGTGGCTGCACTTTGCCAATAGCGTGTTTAATCGCCTGGAAGATCTGCCGGTGCCGACCATTGCTGCCGTCAATGGCTATGCGCTGGGCGGTGGCTGCGAATGCGTGCTGGCGACCGATTATCGTCTGGCGACGCCGGATCTGCGCATCGGTCTGCCGGAAACCAAACTGGGCATCATGCCTGGCTTTGGCGGTTCTGTACGTATGCCACGTATGCTGGGCGCTGATAGTGCGCTGGAAATCATTGCCGCCGGTAAAGATGTCGGCGCGGATCAGGCGCTGAAAATCGGTCTGGTGGAT

1474 GGCGTAGTCAAAGCAGAAAAACTGGTTGAAGGCGCAAAGGCGGTTTTAC

1475 GCCAGGCCATTAACGGCGACCTCGACTGGAAAGCAAAACGTCAGCCGAA

1476 GCTGGAACCACTAAAACTGAGCAAGATTGAAGCCACCATGAGCTTCACCA

1477 TCGCTAAAGGGATGGTCGCGCAAACCGCGGGGAAGCATTATCCGGCCCCC

1478 ATCACCGCAGTAAAAACCATTGAAGCTGCGGCCCGTTTTGGTCGTGAAGA

1479 AGCCTTAAACCTGGAAAATAAAAGTTTTGTCCCGCTGGCGCATACCAACG

1480 AAGCCCGCGCACTGGTCGGCATTTTCCTTAACGATCAATATGTAAAAGGC

1481 AAAGCGAAGAAACTCACCAAAGACGTTGAAACCCCGAAGCAGGCCGCGG

1482 TGCTGGGTGCAGGCATTATGGGCGGCGGCATCGCTTACCAGTCTGCGTGG

1483 AAAGGCGTGCCGGTTGTCATGAAAGATATCAACGATAAGTCGTTAACCCT

1484 CGGCATGACCGAAGCCGCGAAACTGCTGAATAAGCAGCTTGAGCGCGGC

1485 AAGATCGATGGTCTGAAACTGGCTGGCGTGATCTCCACCATCCACCCAAC

1486 GCTCGACTACGCCGGATTTGACCGCGTGGATATTGTGGTAGAAGCGGTTG

1487 TTGAAAACCCGAAAGTGAAAAAAGCCGTACTGGCAGAAACCGAGCAAAA

1488 AGTACGCCAGGATACCGTGCTGGCGTCTAATACTTCAACCATTCCTATCAG

1489 CGAACTGGCCAACGCGCTGGAACGCCCGGAAAACTTCTGCGGGATGCACT

1490 TCTTTAACCCGGTCCACCGAATGCCGTTGGTAGAAATTATTCGCGGCGAG

1491 AAAAGCTCCGACGAAACCATCGCGAAAGTTGTCGCCTGGGCGAGCAAGAT

1492 GGGCAAGACGCCGATTGTGGTTAACGACTGCCCCGGCTTCTTTGTTAACCG

1493 CGTGCTGTTCCCGTATTTCGCCGGTTTCAGCCAGCTGCTGCGCGACGGCGC

1494 GGATTTCCGCAAGATCGATAAAGTGATGGAAAAGCAGTTTGGCTGGCCGA

1495 TGGGCCCGGCATATCTGCTGGACGTTGTGGGCATTGATACCGCGCATCAC

GCTCAGGCTGTCATGGCAGCAGGCTTCCCGCAGCGGATGCAGAAAGATTA
CCGCGATGCCATCGACGCGCTGTTTGATGCCAACCGCTTTGGTCAGAAGA
ACGGCCTCGGTTTCTGGCGTTATAAAGAAGATAGCAAAGGTAAGCCGAAG
AAAGAAGAAGACGCCGCCGTTGAAGACCTGCTGGCAGAAGTGAGCCAGC
CGAAGCGCGATTTCAGCGAAGAAGAGATTATCGCCCGCATGATGATCCCG
ATGGTCAACGAAGTGGTGCGCTGTCTGGAGGAAGGCATTATCGCCACTCC
GGCGGAAGCGGATATGGCGCTGGTCTACGGCCTGGGCTTCCCTCCGTTCC
ACGGCGGCGCGTTCCGCTGGCTGGATACCCTCGGTAGCGCAAAATACCTC
GATATGGCGCAGCAATATCAGCACCTCGGCCCGCTGTATGAAGTGCCGGA
AGGTCTGCGTAATAAAGCGCGTCATAACGAACCGTACTATCCTCCGGTTG
AGCCAGCCCGTCCGGTTGGCGACCTGAAAACGGCTTAA

Gene sequence of native Ter (Ter(N)): Containing 11 MazF recognition sites

ATGTTCACCACCACCGCTAAAGTTATCCAGCCGAAAATCCGTGGTTTCATC
TGCACCACCACCCACCCGATCGGTTGCGAAAAACGTGTTCAGGAAGAAAT
CGCTTACGCTCGTGCTCACCCGCCGACCTCTCCGGGTCCGAAACGTGTTCT
GGTTATCGGTTGCTCTACCGGTTACGGTCTGTCTACCCGTATCACCGCTGC
TTTCGGTTACCAGGCTGCTACTCTCGGTGTCTTCCTGGCGGGTCCGCCGAC
CAAAGGTCGTCCGGCTGCTGCTGGTTGGTACAATACTGTTGCGTTCGAAA
AAGCTGCGCTGGAAGCTGGTCTGTACGCTCGTTCTCTGAATGGCGACGCG
TTCGACTCTACCACTAAAGCTCGTACCGTTGAAGCTATCAAACGTGACCTG
GGTACTGTTGACCTGGTTGTTTACTCTATCGCTGCTCCGAAACGTACCGAC

CCGGCTACCGGTGTTCTGCACAAAGCGTGCCTGAAACCGATCGGTGCTACCTACACCAACCGTACCGTTAACACCGACAAAGCTGAAGTTACCGACGTTTCTATCGAACCGGCTTCTCCGGAAGAAATCGCTGACACCGTTAAAGTTATGGGTGGTGAAGACTGGGAACTGTGGATACAGGCTCTGTCTGAAGCTGGTGTTCTGGCTGAAGGTGCTAAAACCGTTGCTTACTCTTACATCGGTCCGGAAATGACTTGGCCGGTATACTGGAGCGGTACTATCGGTGAAGCTAAAAAAGACGTTGAAAAAGCTGCTAAACGTATCACCCAGCAGTACGGTTGCCCGGCTTACCCGGTTGTTGCTAAAGCTCTGGTTACCCAGGCTTCTTCTGCTATCCCGGTTGTTCCGCTGTACATCTGCCTGCTGTACCGTGTTATGAAAGAAAAAGGTACTCACGAAGGTTGCATCGAACAGATGGTTCGTCTGCTGACCACCAAACTGTACCCGGAAAACGGTGCTCCGATCGTTGACGAAGCTGGTCGTGTTCGTGTTGACGACTGGGAAATGGCTGAAGACGTTCAGCAGGCTGTTAAAGACCTCTGGTCGCAGGTAAGCACCGCTAACCTGAAAGACATCTCTGACTTCGCTGGTTACCAGACCGAATTTCTGCGTCTGTTCGGTTTCGGTATCGACGGTGTTGACTACGACCAGCCGGTTGACGTTGAAGCTGACCTGCCGTCTGCTGCTCAGCAGTAA

Gene sequence of modified Ter (Ter(M)): Without MazF recognition sites

ATGTTCACCACCACCGCTAAAGTTATCCAGCCGAAAATCCGTGGTTTCATCTGCACCACCACCCACCCGATCGGTTGCGAAAAACGTGTTCAGGAAGAAATCGCTTACGCTCGTGCTCACCCGCCGACCTCTCCGGGTCCGAAACGTGTTCTGGTTATCGGTTGCTCTACCGGTTACGGTCTGTCTACCCGTATCACCGCTGCTTTCGGTTACCAGGCTGCTACTCTCGGTGTCTTCCTGGCGGGTCCGCCGAC

CAAAGGTCGTCCGGCTGCTGCTGGTTGGTATAATACTGTTGCGTTCGAAAA
AGCTGCGCTGGAAGCTGGTCTGTACGCTCGTTCTCTGAATGGCGACGCGTT
CGACTCTACCACTAAAGCTCGTACCGTTGAAGCTATCAAACGTGACCTGG
GTACTGTTGACCTGGTTGTTTACTCTATCGCTGCTCCGAAACGTACCGACC
CGGCTACCGGTGTTCTGCATAAAGCGTGCCTGAAACCGATCGGTGCTACC
TATACCAACCGTACCGTTAATACCGATAAAGCTGAAGTTACCGACGTTTCT
ATCGAACCGGCTTCTCCGGAAGAAATCGCTGATACCGTTAAAGTTATGGG
TGGTGAAGACTGGGAACTGTGGATTCAGGCTCTGTCTGAAGCTGGTGTTCT
GGCTGAAGGTGCTAAAACCGTTGCTTACTCTTATATCGGTCCGGAAATGA
CTTGGCCGGTATACTGGAGCGGTACTATCGGTGAAGCTAAAAAAGACGTT
GAAAAAGCTGCTAAACGTATCACCCAGCAGTACGGTTGCCCGGCTTACCC
GGTTGTTGCTAAAGCTCTGGTTACCCAGGCTTCTTCTGCTATCCCGGTTGTT
CCGCTGTATATCTGCCTGCTGTACCGTGTTATGAAAGAAAAAGGTACTCAC
GAAGGTTGCATCGAGCAGATGGTTCGTCTGCTGACCACCAAACTGTACCC
GGAAAACGGTGCTCCGATCGTTGACGAAGCTGGTCGTGTTCGTGTTGACG
ACTGGGAAATGGCTGAAGACGTTCAGCAGGCTGTTAAAGACCTCTGGTCG
CAGGTAAGCACCGCTAACCTGAAAGATATCTCTGACTTCGCTGGTTACCA
GACCGAATTTCTGCGTCTGTTCGGTTTCGGTATCGACGGTGTTGACTACGA
CCAGCCGGTTGACGTTGAAGCTGACCTGCCGTCTGCTGCTCAGCAGTAA

Gene sequence of native YdiI (YdiI(N)): Containing 5 MazF recognition sites

ATGATATGGAAACGGAAAATCACCCTGGAAGCACTGAATGCTATGGGTGA

AGGAAACATGGTGGGGTTCCTGGATATTCGCTTTGAACATATTGGTGATGACACCCTTGAAGCGACAATGCCAGTAGACTCGCGGACAAAGCAGCCTTTCGGGTTGCTGCATGGAGGAGCATCCGTGGTACTGGCCGAAAGTATCGGTTCCGTTGCCGGTTATTTATGTACCGAAGGTGAGCAAAAAGTGGTTGGTCTGGAAATCAATGCTAACCACGTCCGCTCGGCACGAGAAGGGCGGGTGCGCGGCGTATGCAAACCGTTGCATCTCGGTTCGCGTCACCAGGTCTGGCAGATTGAAATCTTCGATGAGAAAGGGCGTTTGTGCTGTTCGTCACGATTGACGACCGCCATTTTGTGA

Gene sequence of modified YdiI (YdiI(M)): Without MazF recognition sites

ATGATATGGAAACGGAAAATCACCCTGGAAGCACTGAATGCTATGGGTGAAGGAAATATGGTGGGGTTCCTGGATATTCGCTTTGAGCATATTGGTGATGATACCCTTGAAGCGACCATGCCAGTAGACTCGCGGACCAAGCAGCCTTTCGGGTTGCTGCATGGAGGAGCATCCGTGGTACTGGCCGAAAGTATCGGTTCCGTTGCCGGTTATTTATGTACCGAAGGTGAGCAAAAAGTGGTTGGTCTGGAAATCAATGCTAACCACGTCCGCTCGGCACGAGAAGGGCGGGTGCGCGGCGTATGCAAACCGTTGCATCTCGGTTCGCGTCACCAGGTCTGGCAGATTGAAATCTTCGATGAGAAAGGGCGTTTGTGCTGTTCGTCACGATTGACGACCGCCATTTTGTGA

DNA sequences of various promoters used in this study

DNA sequences of PluxI

TTTTTGTTCACCTAGCTTATTGTTATGTTTTTTGCGTGTTATATAACACCAATTTGGAGGTTTGGTGATATCGCTTCCAATTAATTCGATCTGGGTCACATTTATGCATCTTGGTGGAAACGTGGTGTTAACATTGCAGCTGTAGGATGGTACAGGTTTCCGTAATGCATCAGTTTGTTATGATCATGTAAAATAAGAGAGGTTGCATGGCTGTA

DNA sequences of PlasI

TGCTCTGATCTTTTCGGACGTTTCTTCGAGCCTAGCAAGGGTCCGGGTTCACCGAAATCTATCTCATTTGCTAGTTATAAAATTATGAAATTTGCATAAATTCTTCAGCTTCCTATTTGGAGGAAGTGAAG

DNA sequences of Pbd (same as Pac)

CTCATCAACTATTTTCCATCACATCTCTGTGATCTAGTTATATTAAAACATGCTAAAAGCATTTATTTCCAATTTTTCTTAACTAGTCGTTTTTTATTCTTAACTGTAAATTTTTTTATGTTAAAATATTAAATACAAATTACATTTAACAGTTAAGTATTTATTTCCTACAGTTAGGCAATATA

DNA sequences of Pc

AACGCGGTCACAAGGAATGCTGTGAAGCGGAAAATCCGCGCGAGTTTATTTCAATTAAAAGACCGTATCTCTCCAGAAATTGATTTTATCGTGATTGCACG

TCCAGGATTGGAAAAGTTATCGTCTGAAGAAGTGAAAGCTAATTTAACACATGTGTTAAATTTAGCTAAAATATTAGATGTAAGAGAGGGAATTGAGTA

DNA sequences of PluxR

TACAGCCATGCAACCTCTCTTATTTTACATGATCATAACAAACTGATGCATTACGGAAACCTGTACCATCCTACAGCTGCAATGTTAACACCACGTTTCCACCAAGATGCATAAATGTGACCCAGATCGAATTAATTGGAAGCGATATCACCAAACCTCCAAATTGGTGTTATATAACACGCAAAAAACATAACAATAAGCTAGGTGAACAAAAA

DNA sequences of PlasR

GTGGGACTGAAATGTGCCTTTCCGGCACAACGCCAACTCTATAGAGTGGGCTGACTGGACATCTTCAGGGGTCGTCGGGCACGGGCGCATGCGCCTCGGCAGGAAGCCGGGATTCTCGGACTGCCGTACAACGTGCCGGATATCGGGTGCCGAATCCATATTTGGCTGATTGGTTAATAGTTTAAGAAGAACGTAGCGCT

DNA sequences of Pr

TTACACCCCTCCTATAAAAACATCTTAACATCTAAGTATTATATTTTCAACTAAAATATGTATTTTTTTTTAAAAAAAAGGGATATAGCTTAATAAACACATCTTCGAGTGTCGTATTCAATACCTAAAATTAAAATATTAACAAATTTTTGTAGTTTATCGAATTTTAGGTCTACTTTTTGATACTTTACATATTAGGAGGAAGAAA

DNA sequences of PprgQ

TTTCTTCCTCCTAATATGTAAAGTATCAAAAAGTAGACCTAAAATTCGATA
AACTACAAAAATTTGTTAATATTTTAATTTTAGGTATTGAATACGACACTC
GAAGATGTGTTTATTAAGCTATATCCCTTTTTTTTAAAAAAAAATACATAT
TTTAGTTGAAAATATAATACTTAGATGTTAAGATGTTTTTATAGGAGGGGT
GTAA

DNA sequences of PlasB

GCGCGTCCCGGAGCTGGGGGCAACCTAGCTGCCACCTGCTTTTCTGCTAGC
TATTCCAGCGAAAACATACAGATTTCCGGCGAAATCAAGGCTACCTGCCA
GTTCTGGCAGGTTTGGCCGCGGGTTCTTTTTGGTACACGAAAGCACCGTCG
AAAACGGGACCGAGCCAGGGGAGTGCAGTTCCTTCTACCCGAAGGACTGA
TACGGCTGTTCCGATCAGCCCACAAGGCGGCGGTAAGCGTCGGCCGAGTA
CTTCGGCCTGAAAAAACCGAGAACTGAACAAG

DNA sequences of PlasA

CTCCGACGAGGAACGCTGAGCGCGCAGACTGTATCGAAGTATTTCCGACG
CCTGGCGTTCTGTGATCGATTCGGCTCGGTTGGAGTGCGCTGCCGCACGCT
TTACAGCCGGACGGAGCGGGGCACCGGATCTACTGCAAAAGCTGATAGTT
GTCTTCCCGGGTGACGGCGTTGCACGGTCCTGCGGCGTGCAACCCGGCCT
GGGCGAAATACCGGCGGAGCGGACCAGGCTATCCCGTCGGAGGCGATGT
CGCCGGGCTGCTGGCTTTCAAGGTTTCCCTTCGATGACCAGGAGCTACCC

DNA sequences of PhcnABC

GTCGGACATGACGGAACGACGGGTGGTGGCGATCTGCGGCGGCAGCGCC
GGGGTCGGGCGAGCGATACACCGGTGACGGCCAGAGGGCGTCGCCGCCA
AGGCGGCGCAGGTGGTCCAGGCGCGCGACCAGGCCGGGCAGGTCACCGT
CGCTGTCGGTGAACGAACGCGGGTAGACCTGGTAGATCACCGCGCGTTTC
CACCAGGGCGGCTGGAAGGATTGCAGCATGGGGCCTCCACGGGTGAGCC
GGCTTTGCTGCTTGGCACCCTGGGGTAGGCGCGGGTTCGACTTTTCCGCGC
GAACGCCTGCACCCCACCTACCAGAATTGGCAGGGGAAGATACCCACCTG
TCATGGATCAATGTCCGCGCCCGGCCCGACTCCTAGTGTCGCCGGCTCCCG
ACTCTCTCTCACGGATGAAAGGG

DNA sequences of Ptrc

TTGACAATTAATCATCCGGCTCGTATAATGTGTGGTCACACAGG

DNA sequences of P1

AAAAAGAGTATTGACTTCGCATCTTTTTGTACCTATAATGTGTGGA

DNA sequences of P2

AAAAAATTTATTTGCTTATTAATTCATCCGGCTCGTATAATGTGTGGA

DNA sequences of P3

TTGACAATTAATCATCCGGCTCGTAATTTATGTGGA

DNA sequences of P4

AAAAAATTTATTTGCTTTCGCATCTTTTTGTACCTATAATGTGTGGA

DNA sequences of P5

TTGCCTCTTAATCATCGGCTCGTATAATGTGTGGA

DNA sequences of P6

TTGACAATTAATCATCCGGCTCTTAGTGTTTGTGGA

References

1 Zhang, Y. *et al.* Using unnatural protein fusions to engineer resveratrol biosynthesis in yeast and mammalian cells. *J. Am. Chem. Soc.* **128**, 13030-13031 (2006).

2 Egland, K. A. & Greenberg, E. P. Quorum sensing in Vibrio fischeri: Analysis of the LuxR DNA binding region by alanine-scanning mutagenesis. *J. Bacteriol.* **183**, 382-386, doi:10.1128/jb.183.1.382-386.2001 (2001).

3 Shepherd, N., Dennis, P. & Bremer, H. Cytoplasmic RNA Polymerase inEscherichia coli. *J. Bacteriol.* **183**, 2527-2534 (2001).
